# How Important Are Dimers for Interpreting the Chiroptical Properties of Carboxylic Acids? A Case Study with [5]-Ladderanoic Acid

**DOI:** 10.1021/acs.jpca.5c04282

**Published:** 2025-10-17

**Authors:** Andrew R. Puente, Prasad L. Polavarapu

**Affiliations:** Department of Chemistry, Vanderbilt University, Nashville, Tennessee 37235, United States

## Abstract

Chiroptical spectroscopy is sensitive to the formation of intermolecular interactions for chiral molecules. Experimental Vibrational Circular Dichroism (VCD), Vibrational Raman Optical Activity (ROA), and Optical Rotatory Dispersion (ORD) data of (−)-[5]-ladderanoic acid in chloroform have been analyzed using theoretical predictions for both monomeric and dimeric structures of (*R*)-[5]-ladderanoic acid to better understand their utility for the interpretation of experimental data. B3LYP, B3PW91, and M06-2X functionals, with and without dispersion corrections, and the 6-31+G­(2d,p) basis set were used for theoretical predictions. It is found that dimer contributions are important to better reproduce the experimental vibrational absorption and associated VCD spectra, and a monomer:dimer ratio of 30:70 is indicated at the B3LYP level. However, no significant improvement is evident from dimer contributions to reproduce the experimental Raman and associated ROA spectra, and a monomer:dimer ratio of 100:0 is indicated at the B3LYP level. Boltzmann population-weighted specific rotations are predicted to be negative for both monomeric and dimeric conformations of (*R*)-[5]-ladderanoic acid, and quantitative agreement with the experimental ORD of (−)-[5]-ladderanoic acid is obtained with a 70:30 mixture of monomers and dimers.

## Graphical abstract

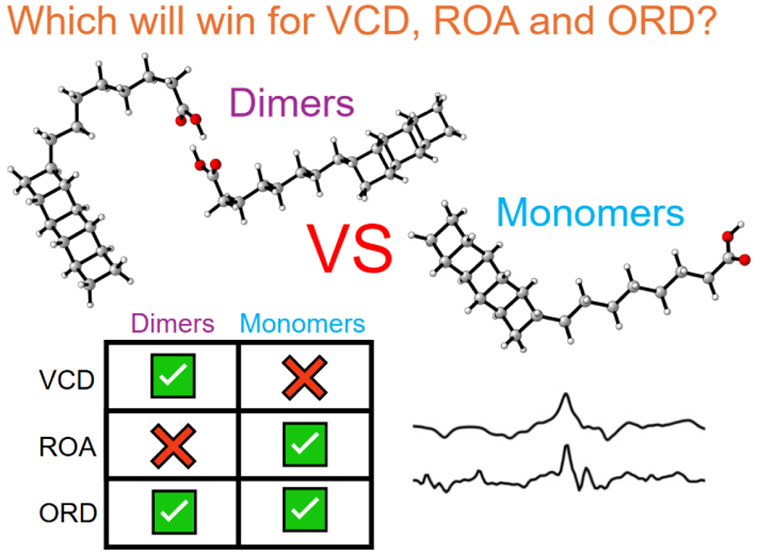

## Introduction

Vibrational Optical Activity (VOA) spectroscopies rely on the differential interaction between left- and right-circularly polarized light with the vibrational modes of chiral molecules.^1–3^ Vibrational Circular Dichroism (VCD) is the differential absorption of infrared circularly polarized light and is widely regarded for its sensitivity to conformational flexibility and the formation of intermolecular interactions.^4–8^ The Raman counterpart, Vibrational Raman Optical Activity (ROA), measures the differential Raman scattering intensities of chiral molecules. VCD and ROA are both inherently weak compared to their achiral counterparts, Vibrational Absorption (VA) and Raman spectroscopies, which results in long collection times and large amounts of sample needed. However, owing to their rich spectral features, VOA spectroscopies serve as powerful tools that can assign the Absolute Configuration (AC) of chiral compounds.^5,9,10^ In particular, ROA spectroscopy is advantageous for its ability to study the solution-phase behavior of chiral biomolecules in water due to the low Raman activity of H_2_O.^11–14^ A thorough characterization of the low-energy conformers in solution is needed to interpret the experimental spectral features in both methods.^15,16^ Accounting for intra- and intermolecular interactions^7,17–23^ and tackling the large conformational ensembles of flexible molecules are active areas of research in VOA spectroscopy owing to the computational challenges these factors introduce into their routine applications.

Chemical compounds containing carboxylic groups are known to form dimers in nonpolar solutions,^24,25^ which can complicate their conformational analysis. There are several strategies for dealing with the formation of dimers, such as esterification, forming sodium salt derivatives^26^ or adding 7-azaindole to replace homodimers with heterodimers.^27^ In hydrogen-bonding (H-bonding) solvents, there is competition between the formation of dimers and solute–solvent complexes due to the solvent’s preference for H-bonding.^24^ Specifically focusing on chiroptical studies of carboxylic acid dimers, VCD has been the main focus of such studies, with very few studies using ROA spectroscopy.^28–35^ Each of these studies demonstrated the sensitivity of VCD to the formation of H-bonding complexes, which sometimes required the modeling of both dimer and monomer contributions to the VCD spectrum to obtain good agreement with the experiment. VCD has also been used to investigate the formation of higher-order aggregates, such as the supramolecular tetramer of (*S*)-2,2’-dimethyl-biphenyl-6,6’-dicarboxylic acid in CDCl_3_.^36^

While outside the scope of this study, Matrix-Isolation VCD (MI-VCD) measurements^37,38^ can be utilized to eliminate solute–solvent interactions from the liquid phase and focus on monomeric species or higher-order aggregates by varying the deposition temperature between low (∼10 K) and high (∼24–30 K), respectively. This technique has been previously used to study three chiral, carboxylic group-containing compounds in rare gas matrices: tetrahydro-2-furoic acid,^39^ lactic acid,^33^ and acetyl-*N*-methyl-l-alanine^40^ (the latter being a methyl ester). In the case of lactic acid, MI-VA and VCD spectra at 24 K were assigned to the trimeric and tetrameric species, and these spectra were found to be similar to those of 0.1 and 0.2 M solutions in CDCl_3_.^33^

Kaminsky and coworkers utilized ROA and VCD spectroscopy to probe the H-bonding intermolecular interactions formed in dimeric structures of lactic and malic acid in H_2_O and D_2_O.^41^ In this work, the authors reported that increasing the concentration of lactic acid in the 1–7 M range and of malic acid in the 1–9 M range had little to no effect on the experimental Raman and ROA spectra. It was also stated that dimers were not the most prevalent species in water and that the ROA was best reproduced from a mixture of contributions from monomer and dimer species. This is likely due to a competing preference between homochiral dimers and solute–solvent complexes. To the best of our knowledge, no ROA studies have been reported that investigated the formation of carboxylic acid dimers in non-H-bonding solvents, such as chloroform (CHCl_3_). Even though CHCl_3_ can function as a H-bond donor to ketones,^42,43^ it is considered as non-H-bonding solvent for carboxylic acids.

The experimental VOA and Optical Rotatory Dispersion (ORD) of naturally occurring [3]- and [5]-ladderanoic acids in chloroform solvent have been reported in 2018 by Raghavan et al.^44^ These molecules possess a rigid ladderane structure combined with a long alkyl side chain terminating with a carboxylic acid (see Figure 1). Crystal structures show dimeric structures, and the propensity of these molecules to form dimers at the solution concentrations used for the VCD and ROA measurements is a distinct possibility. Still, previous calculations were limited to monomer structures due to the computational challenges associated with modeling dimer complexes for large, flexible molecules such as these. From interpretations of the experimental VOA and ORD spectra, using theoretical predictions for monomeric structures, the following observations were made:

The signs of the predicted specific rotations for monomers matched those of the experiment, which led to the assignment of AC of ladderanoic acids.
Correlation between experimental and calculated ROA spectra of monomers gave satisfactory evidence of the AC of ladderanoic acids. The agreement between the experimental and calculated Raman spectra was good, suggesting that correlations in the ROA spectra were trustworthy.
Correlation between experimental and calculated VCD spectra of monomers did not give insight into the AC of ladderanoic acids. Some agreement was obtained for a few VCD bands, but poor correlation between experimental and calculated VA spectra suggested that this agreement was tenuous.

**Figure 1:**
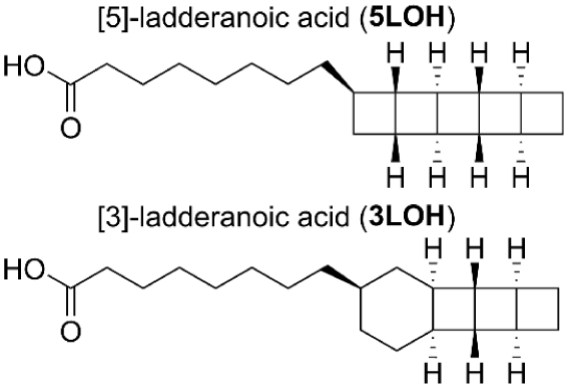
Structures of (*R*)-ladderanoic acids studied previously.

Given that spectral simulations of VCD and ROA rely on the same underlying vibrational frequency calculations, it is puzzling why such good agreement was obtained for ROA and not for VCD. Since the treatment of dimers was not included, the leading hypothesis behind this discrepancy was that ROA is less sensitive to the formation of dimeric structures than VCD. Molecular modeling of the dimer structures introduces additional computational challenges, namely, that (a) the increase in the size of the system drastically increases the cost of the density functional theory (DFT) calculations and (b) it is difficult to properly sample the dimer complexes to obtain a unique set of dimer geometries because each monomer within a dimer can adopt any one of the many monomer conformations. If ROA is truly less sensitive to the formation of dimer complexes, then that will certainly be a distinct advantage for ROA spectroscopy in terms of computational cost.

Harris et al. derivatized the carboxylic acid group in [5]-ladderanoic acid (5LOH) and [3]-ladderanoic acid (3LOH) to that of a methyl ester (−COOCH_3_), which should have inhibited the intermolecular interactions associated with the formation of homochiral dimers.^45^ In the case of these esters, the agreement between predicted spectra, with Gibbs energy-derived populations, and experimental spectra was significantly improved for VA, but it remained poor for VCD, and the predicted signs of specific rotations matched the experiment. This observation suggested that these unusual compounds, with seven CH_2_ groups between the −COOR group and the stereocenter, may be more difficult to model using VCD than anticipated.^45^

It is still unclear what effect dimer modeling will have on the overall VCD and ROA spectra of5LOHand3LOH, given that the −COOH group is located a great distance away from the chiral center. To properly test the utility of modeling dimers for these compounds, we decided to focus on both monomer and dimer contributions to the VCD and ROA spectra of 5LOH, despite the significant computational cost associated with quantum chemical calculations of dimers of this size. This molecule was chosen over 3LOH due to the increased flexibility of 3LOH, which can further complicate the already difficult conformational analysis of dimeric species.

## Methods

### Computational Methods

Extensive conformational searches were undertaken for both monomeric and dimeric (*R*)-5LOH species to obtain representative conformational ensembles of each. Herein, we refer to the dimer species as d5LOH (dimers of 5LOH). The monomer conformational search was performed, independent of previous work,^44^ using Pcmodel 10.0 (GMMX search algorithm)^46^ with a 20 kcal/mol search limit, which located 9133 conformers. The dimer conformational search was performed using the *Quantum Cluster Growth* (QCG)^47,48^ protocol in CREST 2.12,^49,50^ which located 1695 dimer geometries. For the dimer conformational search, the semiempirical GFN2-xTB^51^ method was used with the analytical linearized Poisson–Boltzmann implicit solvation model.^52^

To avoid DFT optimizations of all QCG conformers, geometries from the CREST conformational searches were ranked using single-point energy calculations at the DFT/6-31+G­(2d,p)/PCM level, which is an approach recommended for handling large, flexible molecules.^53,54^ Then, the QCG geometries within the lowest 2.5 kcal/mol based on single-point energies at the DFT/6-31+G­(2d,p)/PCM level were fully optimized at the same level. DFT calculations for dimers were performed using the B3LYP,^55,56^ B3PW91,^55,57^ and M06-2X^58^ functionals with and without Grimme’s empirical dispersion corrections, D3B­(J)^59^ for B3LYP/B3PW91 and D3 ^60^ for M06-2X, with the 6-31+G­(2d,p) basis set and a polarizable continuum model (PCM) representing the chloroform solvent. VCD and ROA calculations were performed at lowest energy optimized geometries, within 2.0 kcal/mol. ROA calculations were performed for an incident laser wavelength of 532 nm. This same process was repeated for the monomer ensemble, except at select levels of theory (see the text). This resulted in 170, 60, and 49 monomer geometries for the B3LYP, B3LYP-D3B­(J), and M06-2X-D3 ensembles, respectively. For dimers, this was 19, 15, and 9 geometries for the B3LYP, B3PW91, and M06-2X ensembles without dispersion, and the ensembles with dispersion were 20, 7, and 5 geometries.

Calculated spectra were simulated using the sum of electronic and zero-point energies (ZPEs) to avoid issues regarding the accurate entropic contributions of low-frequency vibrational modes to thermal Gibbs energies for calculations of dimers.^61^ For consistency, the sum of electronic and ZPEs was also used for simulations of monomer spectra. Spectral simulations were carried out using the in-house developed CDSpecTech program^62^ with Lorentzian band shapes and 10 cm^–1^ half-width.

The comparison between experimental and predicted spectra is assessed using the quantitative similarity measures, *Sim*VA, *Sim*VCD, *Sim*Raman, and *Sim*ROA, respectively, for VA, VCD, Raman, and ROA spectra using the CDSpecTech program. All quantitative similarities are reported for the 1500–1000 cm^–1^ region for VCD and VA spectra and 1800–800 cm^–1^ region for Raman and ROA spectra. Due to interfering solvent Raman bands from CHCl_3_, the 1300–1200 cm^–1^ region was excluded from the calculation of *Sim*Raman and *Sim*ROA. Details regarding this similarity analysis have been reported previously.^63^ For a visual presentation of the spectra, the Raman spectral intensities of monomers and dimers were each scaled down uniformly to match the experimental Raman intensities. However, the magnitudes of the simulated ROA intensities of dimers were scaled down by an additional factor of 0.40 due to their large intensities when compared to the experiment. For generating the spectra of a mixture of monomers and dimers, the native monomer and dimer spectra (each Boltzmann-weighted) were combined in different selected proportions (vide infra) for the B3LYP, B3LYP-GD3B­(J), and M06-2X-GD3 levels of theory. Molecular visualizations were made with CYLview.^64^

### Experimental Methods

Experimental details regarding the measurements of 5LOH have been reported previously^44^ but are summarized below. The experimental VA and VCD spectra were measured in CDCl_3_ with a Biotools ChiralIR spectrometer. The concentration used for these measurements was 24 mg/mL. Measurements were made in BaF_2_ cells with fixed path lengths of 500, 200, and 100 μm. The experimental Raman and ROA spectra were recorded using scattered circular polarization backscattering geometry for the CHCl_3_ solution with a Biotools ChiralRAMAN spectrometer. The concentration was estimated to be >33 mg/mL. A 500 mW laser with a wavelength of 532 nm was used over a 20-h data collection.

## Results and Discussion

The utility of empirical dispersion corrections is a debated topic in the field of chiroptical spectroscopy, with many citing that their inclusion had a negligible effect on the simulated spectra or worsened overall agreement for VCD spectra.^53,65,66^ In the context of modeling solute–solvent interactions with explicit solvent molecules, of which dispersive interactions would be expected to play a significant role, dispersion corrections have been shown to overestimate the favorability of certain solute–solvent binding topologies.^24^ However, there has not been much focus on the role of these corrections in ROA spectroscopy. Dispersion corrections were considered during the QCG search for dimer geometries, as these are incorporated into the semiempirical GFN2-xTB method.^51^ Additionally, dispersion corrections may play a significant role in the modeling of large dimer species, as considered here. For these reasons, we elected to undertake independent calculations with and without Grimme’s empirical dispersion corrections for each of the DFT functionals we have chosen.^59,60^ These corrections indeed have a profound effect on the distribution of low-energy conformers for the B3LYP and B3PW91 functionals (Figure 2). There is a much less significant effect on the conformational ensembles with the M06-2X functional, with the lowest-energy geometry (left M06-2X geometry in Figure 2) remaining the same with and without dispersion. The other M06-2X geometry with a degenerate energy (right M06-2X geometry in Figure 2) is equivalent to the low-energy B3PW91-D3B­(J) geometry.

**Figure 2:**
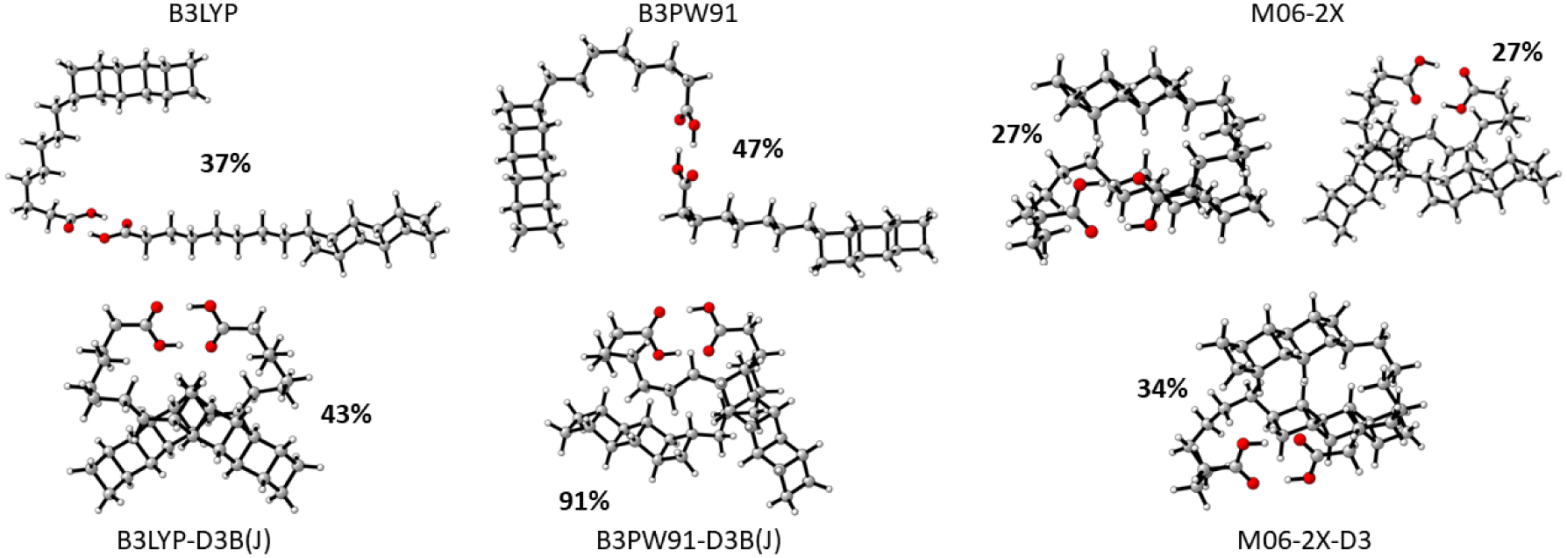
Lowest-energy geometries for 5LOH dimers at each of the six DFT/6-31+G­(2d,p)/PCM levels of theory. The Boltzmann populations based on ZPEs are indicated next to each geometry.

The geometries and energies of the lowest-energy conformers at each level used in this study are presented in the Supporting Information. The dispersion interactions influence both relative energies and structures. While relative energies influence the conformer ensemble, changes in structures can influence the spectral properties. To evaluate these two effects, the low-energy optimized conformers at the B3LYP level were reoptimized at B3LYP-D3B­(J) and M06-2X-D3 levels. Their relative energies, summarized in Table S3, indicate that these different DFT levels indeed provide drastically different conformational preferences. The lowest-energy structure, C139, at the B3LYP level was then reoptimized at B3LYP-D3B­(J) and M06-2X-D3 levels, and their VOA spectra were calculated. These spectra are compared in Figure S4. The similarity overlap values between predicted spectra at the B3LYP and B3LYP-D3B­(J) levels indicate that there are some differences in the predicted spectral properties, but *Sim* values suggest an acceptable correlation between the two. The similarity overlap values between predicted spectra at B3LYP and M06-2X-D3 levels, however, are quite poor.

### Vibrational Absorption and Circular Dichroism

The experimental VA spectrum has six main features: three higher-wavenumber bands at 1462, 1434, and 1411 cm^–1^ as well as a broad group of bands at 1284, 1246, and 1222 cm^–1^ (see Figure 3). The experimental VCD spectrum has a predominant (+)-band at 1238 cm^–1^. Visualization of the normal modes of low energy conformers responsible for this intense band at 1238 cm^–1^ shows that the vibration is −CH_2_ and −CH wagging and rocking along the entire monomer, with additional strong contributions from the −COOH bending. In contrast, these vibrations appear to be separated for the dimer geometries, with the −COOH···HOOC– double H-bond stretching appearing below or at 1000 cm^–1^ at most levels of theory.

**Figure 3:**
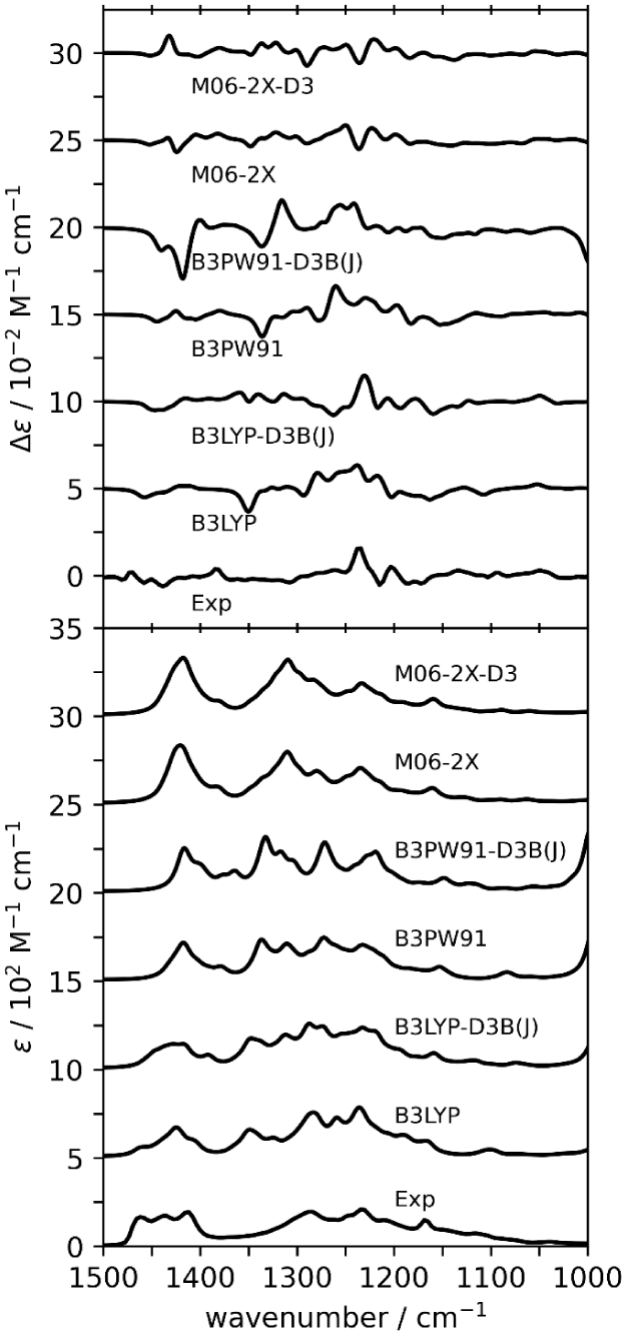
Comparison of experimental and simulated VCD (top) and VA (bottom) spectra for (*R*)-**5LOH** dimers. Scale factors are determined based on maximum *Sim*VA (see Table 1).

The comparison of the experimental and calculated dimer spectra at the DFT/6-31+G­(2d,p)/PCM­(CH_3_Cl) level is presented in Figure 3. Due to the low number of features present in the experimental VCD spectra, simulated spectra are frequency-scaled to give the highest *Sim*VA. These similarities and the scale factors used for Figure 3 are presented in Table 1. The simulated VA spectra at the chosen levels of theory reproduce the experimental VA spectra well, with B3LYP, B3LYP-D3B­(J), M06-2X, and M06-2X-D3 all having the highest *Sim*VA. However, the M06-2X and M06-2X-D3 levels fail to visually reproduce the three high-wavenumber bands in the experimental VA, instead presenting them as one intense band. For VCD, the agreement between experimental and simulated spectra decreases with the inclusion of empirical dispersion corrections. As seen in Table 1, the *Sim*VCD value for dimers decreases from 0.43 to 0.16 when going from B3LYP to B3LYP-D3B­(J); from 0.36 to 0.21 when going from B3PW91 to B3PW91-D3B­(J); and from 0.05 to −0.12 when going from M06-2X to M06-2X-D3 (note that the sign reversal at M06-2X-D3 indicates increased correlation for wrong AC).

**Table 1:** Quantitative Similarities between the Experimental and Simulated VA and VCD Spectra of 5LOH Monomers and Dimers at Different Levels of Theory in the 1500–1000 cm^–1^ Region^a^,^b^,^c^

	Monomers			Dimers		
DFT Functional	σ	*Sim*VA	*Sim*VCD	σ	*Sim*VA	*Sim*VCD
B3LYP	0.976 (0.980)	0.38 (0.39)	0.67 (0.61)	0.972 (0.980)	0.89 (0.86)	0.43 (0.34)
B3LYP-D3B(J)	0.977 (0.977)	0.42 (0.42)	0.54 (0.54)	0.965 (0.977)	0.88 (0.83)	0.16 (0.12)
B3PW91	-	-	-	0.957 (0.970)	0.82 (0.75)	0.36 (0.28)
B3PW91-D3B(J)	-	-	-	0.951 (0.961)	0.75 (0.72)	0.21 (0.20)
M06-2X	-	-	-	0.955 (0.968)	0.84 (0.79)	0.05 (0.20)
M06-2X-D3	0.967 (0.968)	0.51 (0.53)	0.51 (0.50)	0.954 (0.968)	0.83 (0.76)	–0.12 (0.13)

a The similarities for monomers are given based on the scale factor (σ) that gives the maximum *Sim*VCD value.

b The similarities for dimers are given based on σ that gives the maximum *Sim*VA value.

c Values in parentheses are with a universal scale factor as discussed in the section Analysis Using a Universal Scale Factor.

Previous calculations utilizing only geometries of 5LOH monomers were unable to obtain good agreement with experimental VA spectra, indicating the inability of monomeric species to reproduce the VA spectra.^44^ Spectral simulations using the currently optimized monomer geometries also fail to reproduce the features presented in the VA spectra (Figure 4). The *Sim*VA values normally obtained for routine cases are around ∼0.7 or higher, but they are much smaller for the spectra obtained here for monomer structures (Table 1). For this reason, we used *Sim*VCD to determine the optimal scale factors. It should be noted that even though *Sim*VCD values here (see Table 1) came out to be larger than the reliable threshold value of 0.40, the accompanying *Sim*VA values are poor. This makes the agreement between experimental data and monomer predictions tenuous. The failure of *Sim*VA values is due to the lack of simulated VA bands in the 1300 to 1220 cm^–1^ region, which instead appear as an intense band at ∼1100 cm^–1^. The visualization of normal modes of low energy conformers responsible for the bands in the 1240 to 1200 cm^–1^ region suggests that the VCD bands in this region are a result of very delocalized −CH_2_ and −CH wagging and rocking.

**Figure 4:**
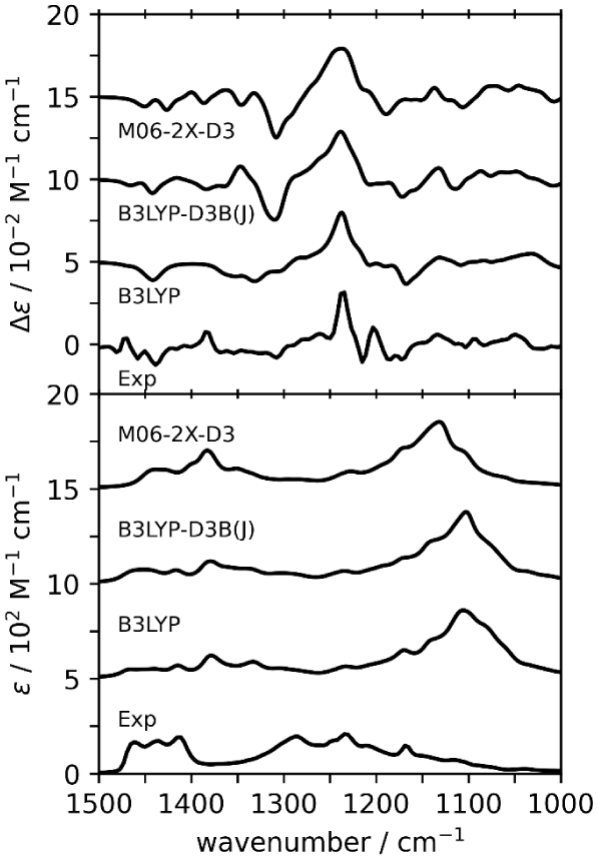
Comparison of experimental and simulated VCD (top) and VA (bottom) spectra for (*R*)-**5LOH** monomers. Scale factors are determined based on maximum *Sim*VCD (see Table 1).

Based on the *Sim*VA and *Sim*VCD values presented in Table 1, the B3LYP/6-31+G­(2d,p)/PCM level prediction for dimer structures best reproduces the experimental spectra, with a *Sim*VA of 0.89 and *Sim*VCD of +0.43. This satisfies the 0.4 threshold deemed necessary for a confident AC assignment.^63^ This high similarity is likely a result of the multitude of (+)-bands in the 1270–1205 cm^–1^ region in the simulated spectra. The B3LYP-D3B­(J) level also appears to be a good reproduction of the VA and VCD spectra, except that a quantitative *Sim*VCD value of 0.16 suggests otherwise.

Thus, it appears that “monomer-alone predicted” spectra of (*R*)-5LOH do not represent well the experimental VA and VCD spectra of (−)-5LOH satisfactorily, but “dimer-alone predicted” VCD spectra of (*R*)-5LOH at the B3LYP/6-31+G­(2d,p)/PCM level do provide better similarity to experimental VA and VCD spectra of (−)-5LOH. The agreement in the VA spectra is largely due to the separation of C–H rocking and wagging with the vibrations of the −COOH group, which appear as separate bands in the spectral simulations of dimers but as the same band in spectra of monomers.

### Vibrational Raman and Raman Optical Activity

Compared with the VCD spectra, the experimental ROA spectra are rich in features that can be interpreted with simulated spectra. The comparison between experimental and simulated Raman and ROA spectra of dimers is presented in Figure 5. Each of the levels of theory used in this study reproduces the experimental Raman spectra very well. Quantitative similarities in the 1800–800 cm^–1^ region for dimers, which are presented in Table 2, do not suggest that one level of theory performs significantly better than the others. The *Sim*ROA values are generally quite low when one considers how many experimental Raman bands are reproduced in the simulated spectra. The highest *Sim*ROA value of 0.32 is given by the M06-2X-D3 level.

**Figure 5:**
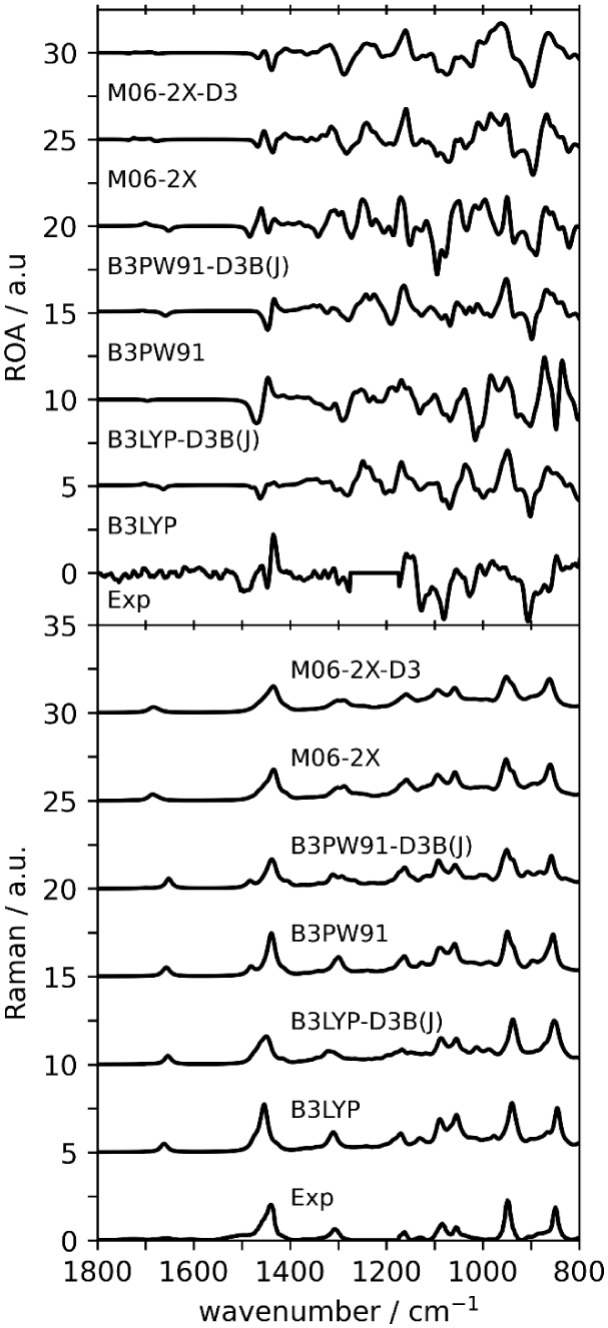
Comparison of experimental and simulated ROA (top) and Raman (bottom) spectra for (*R*)-**5LOH** dimers. Simulated ROA intensities are scaled by 0.40 for visualization purposes. Scale factors are determined based on maximum *Sim*Raman (see Table 2). The 1300–1200 cm^–1^ region of the experimental ROA spectrum is blocked off due to solvent interference.

**Table 2:** Quantitative Similarities between the Experimental and Simulated Raman and ROA Spectra of 5LOH Dimers at Different Levels of Theory^a^,^b^

	1800–800 cm^–1^			1200–800 cm^–1^		
DFT Functional	σ	*Sim*Raman	*Sim*ROA	σ	*Sim*Raman	*Sim*ROA
B3LYP	0.987 (0.980)	0.68 (0.64)	0.12 (0.07)	0.993 (0.980)	0.73 (0.57)	0.17 (0.09)
B3LYP-D3B(J)	0.982 (0.977)	0.66 (0.63)	0.21 (0.19)	0.988 (0.977)	0.71 (0.62)	0.23 (0.18)
B3PW91	0.982 (0.970)	0.74 (0.53)	0.07 (−0.01)	0.979 (0.970	0.74 (0.62)	0.08 (0.04)
B3PW91-D3B(J)	0.98 (0.961)	0.67 (0.43)	0.23 (0.26)	0.975 (0.961)	0.69 (0.52)	0.29 (0.31)
M06-2X	0.974 (0.968)	0.64 (0.61)	0.29 (0.29)	0.969 (0.968)	0.68 (0.68)	0.37 (0.37)
M06-2X-D3	0.974 (0.968)	0.64 (0.61)	0.32 (0.31)	0.968 (0.968)	0.68 (0.68)	0.41 (0.41)

a The similarities are given based on the scale factor (σ) that gives the maximum *Sim*Raman value in the denoted region.

b Values in parentheses are with a universal scale factor as discussed in the section Analysis Using a Universal Scale Factor.

In the ∼1800–1300 cm^–1^ region, there are three experimental ROA bands at (−)-1494, (−)-1447, and (+)-1433 cm^–1^. These bands are not well reproduced by the levels of theory chosen in this study. The B3LYP-D3B­(J) and B3PW91 simulated spectra for dimers do show a (−,+)-bisignate ROA feature in this region, which appears to correlate well with the 1447 and 1433 cm^–1^ bands.

Given the solvent interference in the 1300–1200 cm^–1^ region and the poor reproduction of the three higher wavenumber bands by all levels of theory, it is worth considering the quantitative similarities in the lower ∼1200–800 cm^–1^ region as opposed to the entire spectrum. These similarities are also presented in Table 2. Focusing on this lower 1200–800 cm^–1^ region, the *Sim*ROA for the M06-2X and M06-2X-D3 functionals both increase by 0.08 and 0.09, respectively (compared to those in 1800–800 cm^–1^ region). This results in the M06-2X-D3 spectral similarities satisfying the +0.4 threshold for *Sim*ROA. The *Sim*Raman values also increase for all levels of theory, indicating that correlations in the Raman and ROA spectra are trustworthy.

To investigate the performance of monomers for the interpretation of ROA spectra, we performed DFT calculations on the monomer ensemble with the B3LYP, B3LYP-D3B­(J), and M06-2X-D3 functionals (Figure 6). B3LYP and B3LYP-D3B­(J) were chosen based on their overall performance for the VA spectra of dimers, while M06-2X-D3 was chosen for its performance for the Raman and ROA spectra of dimers. In the 1800–800 cm^–1^ region, the *Sim*ROA for monomers (see Table 3) increases for B3LYP and B3LYP-D3B­(J) compared to the dimer spectral similarities (Table 2) at the same levels, but quantitative similarities for monomers are still not high enough to claim good agreement. When the lower 1200–800 cm^–1^ region is considered, the *Sim*ROA increases for monomers to +0.40 (from 0.17 for dimers) at the B3LYP and to +0.34 (from 0.23 for dimers) at the B3LYP-D3B­(J) level. At the M06-2X-D3 level, *Sim*ROA remains about the same for monomers (0.40) and dimers (0.41). This level of better agreement across multiple levels of theory is not seen for the dimer calculations, of which only the M06-2X-D3 level was above the +0.4 threshold for a confident AC assignment. Therefore, the “monomer-alone predicted” spectra of (*R*)-5LOH appear to give better similarity to the experimental ROA spectra of (−)-5LOH.

**Figure 6:**
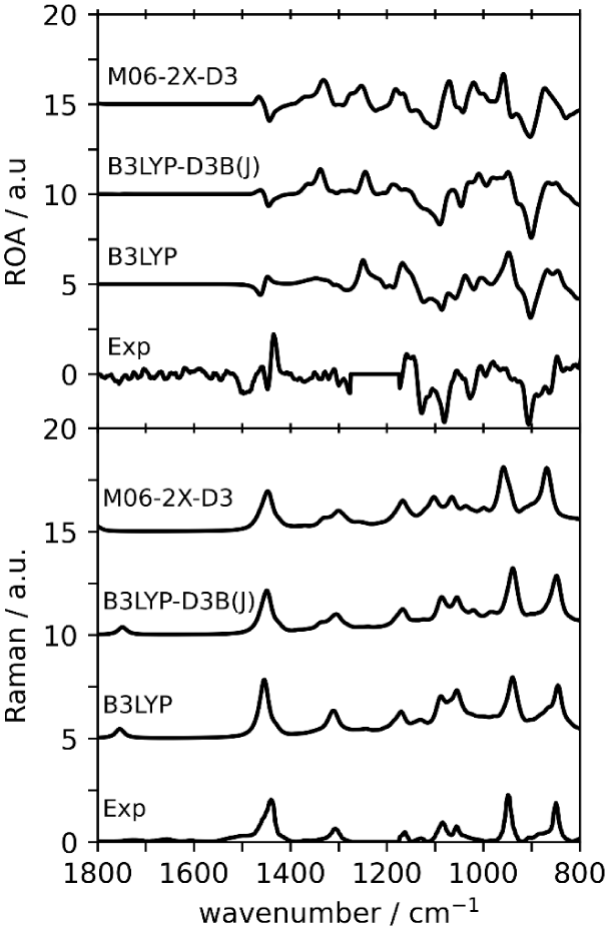
Comparison of experimental and simulated ROA (top) and Raman (bottom) spectra for (*R*)-**5LOH** monomers. Scale factors are determined based on maximum *Sim*Raman in the 1800 to 800 cm^–1^ region (see Table 3). The 1300–1200 cm^–1^ region of the experimental ROA spectrum is blocked off due to solvent interference.

**Table 3:** Quantitative Similarities between the Experimental and Simulated Raman and ROA Spectra of **5LOH** Monomers at Different Levels of Theory^a^,^b^

	1800–800 cm^–1^			1200–800 cm^–1^		
DFT Functional	σ	*Sim*Raman	*Sim*ROA	σ	*Sim*Raman	*Sim*ROA
B3LYP	0.987 (0.980)	0.66 (0.62)	0.37 (0.36)	0.993 (0.980)	0.73 (0.57)	0.4 (0.4)
B3LYP-D3B(J)	0.984 (0.977)	0.69 (0.63)	0.28 (0.32)	0.988 (0.977)	0.75 (0.63)	0.34 (0.48)
M06-2X-D3	0.973 (0.968)	0.63 (0.60)	0.19 (0.27)	0.968 (0.968)	0.71 (0.71)	0.4 (0.4)

a The similarities are given based on the scale factor (σ) that gives the maximum *Sim*Raman value in the denoted region.

b Values in parentheses are with a universal scale factor as discussed in the section Analysis Using a Universal Scale Factor.

### Monomer:Dimer Ratio Analysis

Thus far, we have separately investigated the performance of monomers and dimers in reproducing the experimental VA/VCD and Raman/ROA spectra. To explore if the agreement of the “monomer alone” and “dimer alone” simulated spectra with the experiment can be improved, we combined simulated spectra of monomers and dimers in ratios from 100% monomer to 0% monomer, incrementing by 10% (see Figure S1). The combined theoretical spectra were then swept against the experimental spectra to determine the maximum similarities. For combined VA and VCD spectra, the scale factor used corresponds to maximum *Sim*VCD value, as reported in Table S1 (note: *Sim*VCD was used because monomer VA spectra do not yield a maximum *Sim*VA overlap in the normal range of frequency values). The monomer:dimer ratios that gave the highest *Sim*VA, for each level of theory, are extracted from Table S1 and summarized in Table 4. The combined spectra for monomer:dimer ratios given in Table 4 are presented in Figure 7.

**Figure 7:**
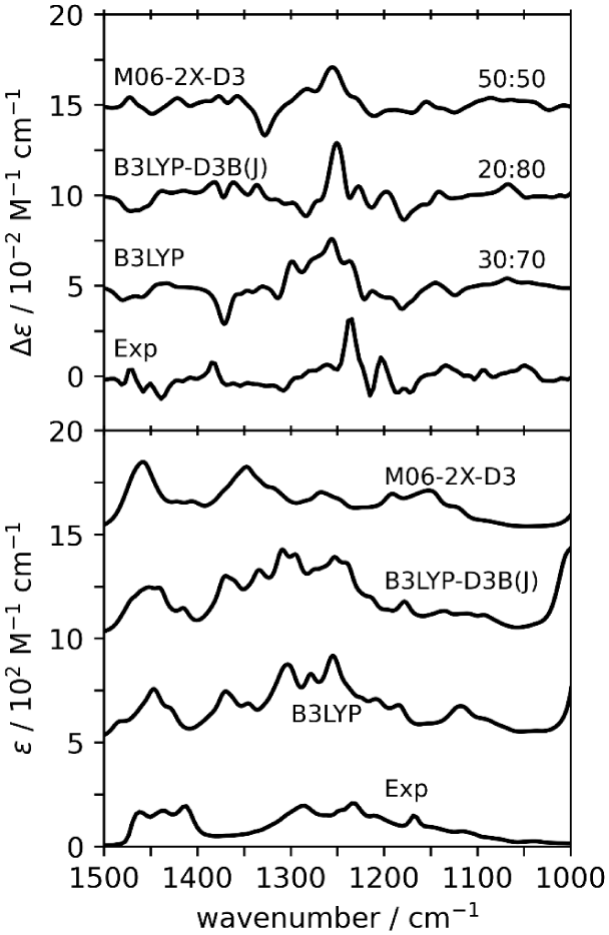
Comparison of experimental and simulated VCD (top) and VA (bottom) for combined spectra of monomer and dimer spectra. The quantitative similarities are presented in Table 4 and S1.

**Table 4:** Monomer:Dimer Ratio That Yields the Highest *Sim*VA Value at Different Levels of Theory^a^,^b^

	1500–1000 cm^–1^			
DFT Functional	Monomer to dimer ratio	σ	*Sim*VA	*Sim*VCD
B3LYP	30:70	0.969 (0.980)	0.89 (0.88)	0.46 (0.37)
B3LYP-D3B(J)	20:80	0.971 (0.977)	0.89 (0.85)	0.28 (0.18)
M06-2X-D3	50:50	0.967 (0.968)	0.82 (0.82)	0.29 (0.29)

a Details of variations of similarity values for different monomer/dimer ratios at each level of theory are given in Table S1.

b Values in parentheses are with a universal scale factor as discussed in the section Analysis Using a Universal Scale Factor.

For the VA and VCD spectra, the combined monomer:dimer spectra improve the *Sim*VCD with minimal changes to the *Sim*VA compared to dimer-alone spectra. When compared to the monomer-alone spectra, the *Sim*VA is significantly improved, but the maximum *Sim*VCD seen for monomer:dimer mixtures is lower than that of monomer-alone. However, the low *Sim*VA associated with the high similarity seen for monomer-alone VCD spectra makes it untrustworthy. The monomer:dimer ratios presented in Table 4 appear to be a good compromise between the untrustworthy *Sim*VCD of monomer-alone spectra and the high *Sim*VA agreement when dimer-alone spectra are used. Of these ratio spectra, the one with a monomer:dimer ratio of 30:70 gives, at the B3LYP level, both the highest *Sim*VA and *Sim*VCD overlap. This seems to indicate that dimer contributions are necessary in order to better reproduce the experimental VA and VCD spectra together. At B3LYP-D3B­(J) and M06-2X-D3 levels, the monomer:dimer compositions with highest *Sim*VA do not have large enough *Sim*VCD to provide a reliable conclusion of the composition.

For combined monomer:dimer Raman/ROA spectra (see Figure S2), both the 1800–800 and 1200–800 cm^–1^ regions were used separately to find the frequency scale factor that gave the highest *Sim*Raman (see Table S2). The monomer:dimer ratios that gave the highest *Sim*ROA, for each level of theory, are extracted from Table S2 and summarized in Table 5. Notably, B3LYP geometries give an optimal monomer:dimer ratio of 100:0 for the 1800–800 cm^–1^ region and 100:0 for the 1200–800 cm^–1^ region, indicating that dimer contributions do not improve overall agreement with experimental ROA spectra in this case. On the contrary, monomer contributions to spectra at the M06-2X-D3 level in the 1800–800 cm^–1^ region do not improve overall quantitative similarity with the experimental spectra. However, B3LYP-D3B­(J) in both regions and M06-2X-D3 in the 1200–800 cm^–1^ region indicate that a monomer:dimer ratio of approximately 80:20 or 70:30 gives a noticeable improvement to the *Sim*ROA without a compromise in the *Sim*Raman values. The combined spectra for monomer:dimer ratios given in Table 5 are presented in Figure 8.

**Table 5:** Monomer:Dimer Ratio That Yields the Highest *Sim*ROA Value at Different Levels of Theory^a^,^b^

	1800–800 cm^–1^				1200–800 cm^–1^			
DFT Functional	Monomer to dimer ratio	σ	*Sim*Raman	*Sim*ROA	Monomer to dimer ratio	σ	*Sim*Raman	*Sim*ROA
B3LYP	100:0	0.987 (0.980)	0.66 (0.62)	0.37 (0.36)	100:0	0.993 (0.980)	0.73 (0.57)	0.40 (0.40)
B3LYP-D3B(J)	70:30	0.983 (0.977)	0.68 (0.63)	0.36 (0.36)	80:20	0.988 (0.977)	0.74 (0.63)	0.39 (0.46)
M06-2X-D3	0:100	0.974 (0.968)	0.64 (0.61)	0.32 (0.31)	70:30	0.968 (0.968)	0.7 (0.7)	0.47 (0.47)

a Details on variations of similarity values for different monomer–dimer ratios at each level of theory are given in Table S2.

b Values in parentheses are with a universal scale factor as discussed in the section Analysis Using a Universal Scale Factor.

**Figure 8:**
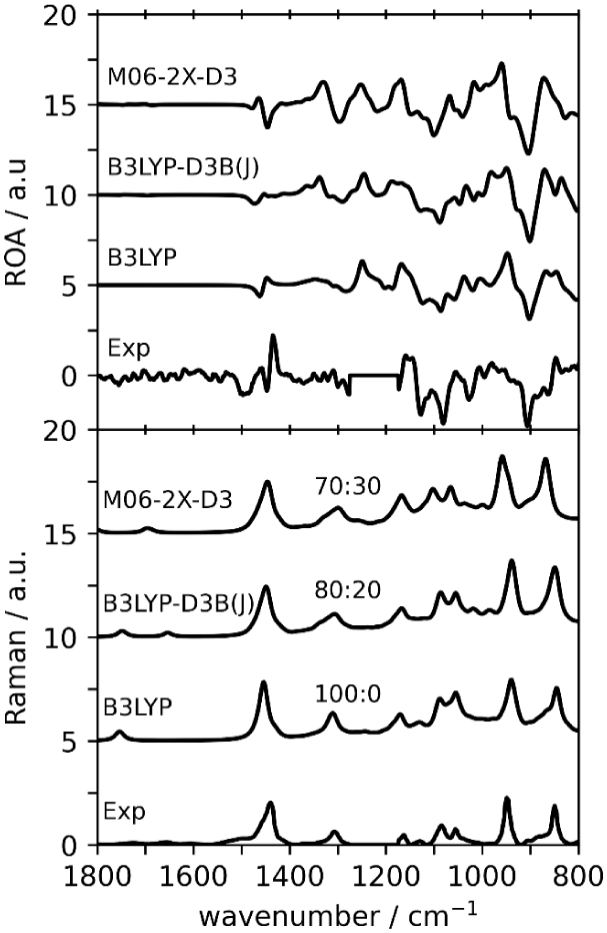
Comparison of experimental and simulated ROA (top) and Raman (bottom) spectra for combined monomer and dimer spectra. The quantitative similarities are presented in Table 5 and S2. The 1300–1200 cm^–1^ region of the experimental ROA spectrum is blocked off due to solvent interference.

The difficulty in ascertaining a reliable monomer–dimer ratio, in the absence of an experimentally determined ratio, is reminiscent of previously reported difficulties in determining conformer populations using VCD and ROA spectra.^67^ Ideally, the monomer:dimer ratio would be experimentally determined by a concentration-dependent VA study, which was not performed in this case. However, we note that the experimental VA spectra taken at a lower path length show two bands in the C=O stretching region at 1740 and 1709 cm^–1^ (see Figure S3). Comparison with the simulated C=O stretching bands of monomer and dimer spectra suggests that the 1740 cm^–1^ band indicates the presence of the monomer and the 1709 cm^–1^ band indicates the presence of the dimer. The ratio of these two band intensities at the B3LYP level independently suggests a monomer:dimer ratio of 30:70. This ratio is the same as that obtained from the maximum *Sim*VA obtained for B3LYP spectra (see Table 4).

Nevertheless, the use of monomer and dimer VOA spectra to compare against the experiment appears to reveal a pattern. There is consistency in our observations that dimer contributions are not essential for achieving good agreement with experimental Raman and ROA spectra, while the VA and VCD results clearly indicate that dimers are essential for satisfactory agreement. Although not pointed out before, we noticed an analogous dichotomy in the literature with the VCD and ROA of lactic acid in water. Xu and coworkers concluded that (H_2_O)_6_ complexes with lactic acid dimers were the dominant contributors to VCD spectra in water.^32^ Kaminsky et al. stated that since Raman and ROA spectra of lactic acid (and also of malic acid) in water are apparently concentration-independent, experimental Raman and ROA data in water are not useful for inferring the relative concentrations of monomers and dimers.^41^ In the case of lactic acid in water, dimers appeared to be necessary to reproduce the experimental VCD spectra,^32^ but dimers appeared not necessary for reproducing the ROA spectra in water.^41^

All of these observations suggest that more VOA studies of carboxylic acids in water and non-H-bonding solvents will be worthy of future investigations.

### Analysis Using a Universal Scale Factor

In the analysis of 5LOH monomers and dimers using *Sim* indices, the VA and Raman spectra are treated as separate, and different frequency scale factors are utilized for each spectroscopic method. One might say that the scale factors for VA and Raman should be the same if they account for mechanical anharmonicity corrections. Although this opinion is correct, the implication of the scale factor in similarity overlap analysis goes beyond mechanical anharmonicity corrections. In similarity overlap analysis, the similarity indices reach the maximum value when the intensities of the most intense bands and their positions match well in experimental and predicted spectra. The most intense bands may or may not have mechanical anharmonic corrections, but they can have theoretical errors in their predicted band positions and associated intensities. As a result, the scale factor used in similarity overlap analysis indirectly accounts for *all theoretical errors* involved in predicting the spectra. This attribute makes the scale factor in similarity overlap analysis dependent on the spectroscopic method used and the usable spectroscopic region therein.

Nevertheless, it will be informative to investigate the role of a common frequency scale factor in these complementary methods. To address this point, the scale factors that give the maximum *Sim*VA, *Sim*VCD, *Sim*Raman, and *Sim*ROA are found for dimers and averaged to obtain a universal scale factor (USF). All of the 1800–800 cm^–1^ region was utilized for Raman and ROA spectra to include the most number of bands. The same was done for monomers, with the exception of *Sim*VA owing to poor overlap with the experimental spectra, and similar scaling factors were obtained. For consistency and to simplify monomer:dimer analysis using a USF, the USF obtained for dimers was used in the current analysis. These data are included in the same tables that reported the data with variable scale factors (see Tables 1–5, S1 and S2).

As can be seen in Table 1, the *Sim*VA and *Sim*VCD values for monomers and dimers did not change significantly when USF is used. There is some increase in the *Sim*VCD value at the M06-2X and M06-2X-D3 levels, but that value still remained below the reliable threshold. It can be seen in Table 2 that *Sim*Raman and *Sim*ROA values for dimers generally decrease when USF is used in both the 1800–800 and 1200–800 cm^–1^ regions. The same is true for *Sim*Raman and *Sim*ROA values for monomers (Table 3). One exception to this observation is that at the B3LYP-D3B­(J) level, the *Sim*ROA value increased with USF from 0.34 to 0.48.

In monomer–dimer analysis (Table S1), a decrease is seen in *Sim*VCD values with USF, while *Sim*VA values essentially remained the same. As a result, the threshold for the reliability of conclusions from *Sim*VCD has to be lowered from 0.40 if USF were to be used. In the case of Raman and ROA (Table S2), *Sim*Raman and *Sim*ROA values generally decreased when USF is used in both the 1800–800 and 1200–800 cm^–1^ regions. An exception is that at the B3LYP-D3B­(J) level, the *Sim*ROA value for the 1200–800 cm^–1^ region has increased to the above threshold value of 0.40 for monomer:dimer compositions ranging from 100:0 to 70:30; however, it should be noted that the accompanying *Sim*Raman values decreased with the use of USF.

In summary, a general observation from all these data in Tables 1–5 S1 and S2 is that the use of USF does not change the conclusions reached in the previous section with varied scale factors. However, it should be kept in mind that when USF is used, the reliability threshold of 0.40 may have to be lowered for *Sim*VCD and *Sim*ROA values.

### Optical Rotatory Dispersion

Boltzmann-averaged ORD calculations, with populations derived from electronic energies combined with ZPEs, using the 6-31+G (2d,p) basis set for monomers and dimers are presented in Figure 9. Both monomers and dimers have negative ORD curves for (*R*)-5LOH, which match the experimental sign for (−)-5LOH. However, ORD curves of dimers are of a larger magnitude than those observed in the experiment. Meanwhile, ORD curves of monomers are of a larger magnitude at B3LYP and a smaller magnitude at B3LYP-D3 (J) and M06-2X-D3 levels compared to the experiment. When monomer and dimer ORD curves for M06-2X-D3 are combined in a 70:30 ratio, which was obtained from ROA quantitative similarity analysis (Table 5), the ORD magnitudes are in near quantitative agreement with the experiment.

**Figure 9:**
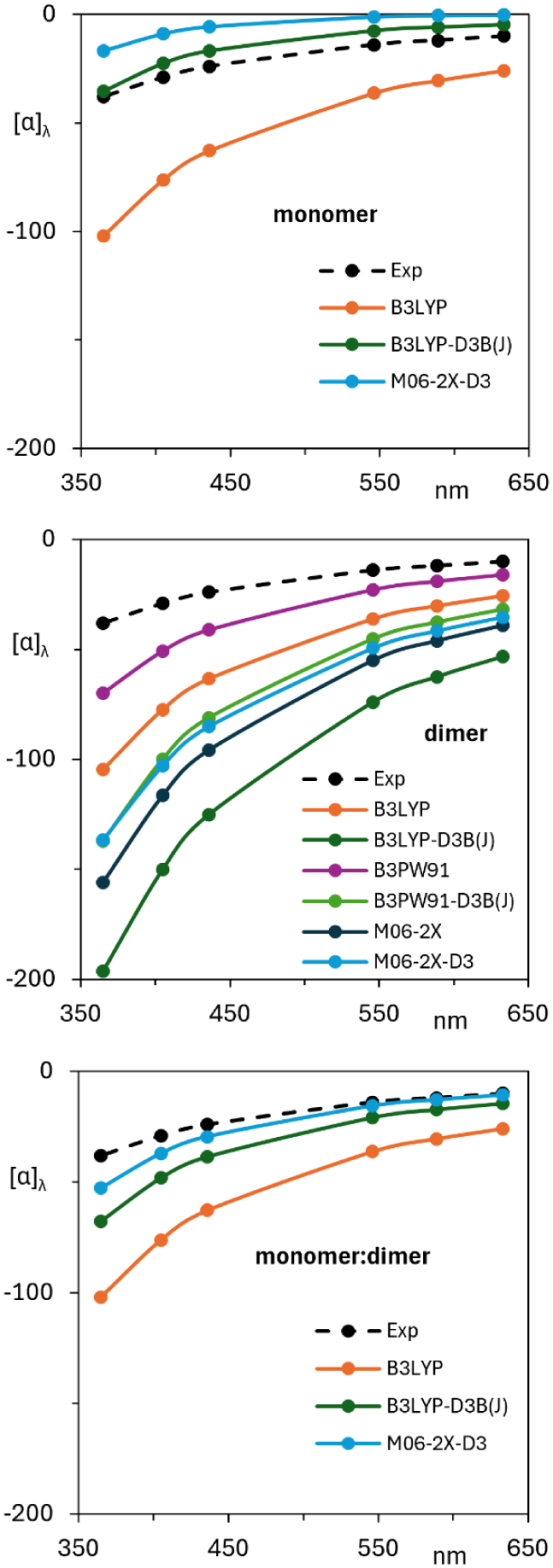
Comparison of experimental and simulated ORD of monomers (top), dimers (middle), and combined monomer and dimer spectra (bottom). The ratios for combined spectra are 100:0 for B3LYP, 80:20 for B3LYP-D3B­(J), and 70:30 for M06-2X-D3.

## Conclusions

Herein, we investigated the utility of modeling monomers and dimers for a large, flexible ladderanoic acid (*R*)-5LOH. Calculations using monomers alone gave very poor agreement with experimental VA spectra. Calculations using dimers alone greatly improved the quantitative similarities for the VA spectra, while also achieving a satisfactory *Sim*VCD, at the B3LYP/6-31+G­(2d,p)/PCM level. Combining monomer and dimer spectra achieves a good balance between the *Sim*VA agreement of dimer-alone spectra and the *Sim*VCD agreement of monomer-alone spectra, with a higher contribution of dimers (30:70 with B3LYP spectra) being necessary to achieve good agreement in both the *Sim*VA and *Sim*VCD values. This observation suggests that the modeling of dimers is crucial to the interpretation of VA and VCD spectra of (*R*)-5LOH.

Monomers alone perform very well in terms of quantitative similarity with the experimental Raman spectra and satisfactorily with the experimental ROA spectra, especially in the 1200–800 cm^–1^ region, at B3LYP and M06-2X-D3 levels. Dimers alone also perform well in terms of quantitative similarity with the experimental Raman spectra but fail to achieve satisfactory quantitative similarity with experimental ROA spectra at all levels, except at M06-2X-D3 in the 1200–800 cm^–1^ region. Combining monomer and dimer spectra, when compared to monomer-alone spectra, quantitative similarity with experimental Raman spectra did not change significantly, but that with experimental ROA spectra decreased at the B3LYP level and improved at M06-2X-D3 and B3LYP-D3B­(J) levels, in both 1800–800 and 1200–800 cm^–1^ regions. Based on quantitative similarity values in the 1200–800 cm^–1^ region, the suggested monomer:dimer composition varied from 100:0 at B3LYP to 80:20 at B3LYP-D3B­(J) and 70:30 at M06-2X-D3 levels of theory.

ORD curves of monomers and dimers both have the same sign as that of the experiment, indicating that either monomers or dimers can be used for assigning the AC via the sign of ORD alone. Quantitative agreement with experimental ORD was obtained if monomer and dimer ORD contributions are combined in a 70:30 ratio at the M06-2X-D3/6-31+G­(2d,p) level, but it is difficult to trust this composition without additional independent evidence.

The inclusion of dispersion corrections to dimer calculations with the B3LYP, B3PW91, and M06-2X functionals increases the *Sim*ROA in both the 1800–800 and the 1200–800 cm^–1^ regions when compared to calculations without dispersion corrections. In contrast, the inclusion of dispersion corrections appears to worsen the agreement between the experimental and simulated VCD spectra of 5LOH dimers. This is concerning because the conformational ensemble is significantly different with and without dispersion. This would seem to imply that one set of conformers performs better for reproducing the VA and VCD spectra, while another set of conformers performs better for reproducing the Raman and ROA spectra. We have noted this dichotomy previously in the literature.^67^ Given this contradiction and poor performance for VA and VCD spectra with dispersion corrections, caution is warranted in the use of dispersion corrections, in general, for the use of chiroptical investigations.

It remains to be seen whether the current observations extend to other chiral carboxylic acids. To further investigate this, it is likely that several more combined VCD, ROA, and ORD studies of carboxylic acids will be necessary to quantitate the utility of modeling dimers for the interpretation of chiroptical spectra. In that context, it might be useful to focus on systems with less conformational complexity than those in 5LOH.

## Supplementary Material



## Notes

Simulated VCD and VA of monomers and dimers combined in ratios of 100% monomer (100:0) to 100% dimer (0:100) with the B3LYP, B3LYP-D3B­(J), and M06-2X-D3 functionals (Figure S1); simulated ROA and Raman of monomers and dimers combined in ratios of 100% monomer (100:0) to 100% dimer (0:100) with the B3LYP, B3LYP-D3B­(J), and M06-2X-D3 functionals (Figure S2); a comparison of the C=O stretching region of experimental, monomer, and dimer VA and VCD spectra (Figure S3); VA and VCD monomer–dimer analysis for the B3LYP, B3LYP-D3B­(J), and M06-2X-D3 functionals (Table S1); Raman and ROA monomer–dimer analysis for the B3LYP, B3LYP-D3B­(J), and M06-2X-D3 functionals (Table S2); a comparison of the C=O stretching region of simulated B3LYP spectra of dimers (top), monomers (middle), and the experiment (bottom) (Figure S3); a comparison of the low-energy B3LYP dimer geometries that are subsequently optimized at the B3LYP-D3B­(J) or M06-2X-D3 levels (Table S3); a comparison of VA, VCD, Raman, and ROA of the lowest-energy d5LOH B3LYP conformer, C139, at the B3LYP, B3LYP-D3B­(J), and M06-2X-D3 functionals (Table S4); and optimized geometries and energies: for 5LOH monomers and d5LOH dimers at all levels of theory (PDF)
