# Supplementary material for: How Important Are Dimers for Interpreting the Chiroptical Properties of Carboxylic Acids? A Case Study with [5]-Ladderanoic Acid
Source: J Phys Chem A. 2025 Oct 17;129(43):9871–82. doi: 10.1021/acs.jpca.5c04282 (PMC12581143; doi:10.1021/acs.jpca.5c04282)
Supplement: Supplementary file 1 [file jp5c04282_si_001.pdf]

Supporting Information

How Important are Dimers for Interpreting the Chiroptical Properties of Carboxylic Acids?: A Case Study with [5]-Ladderanoic Acid

Andrew R. Puente[a], Prasad L. Polavarapu[a]

[a]Department of Chemistry, Vanderbilt University, Nashville, TN 37235

Summary of Supplementary Information

Figure S1: Simulated VCD and VA of monomers and dimers combined in ratios of 100% monomer (100:0) to 100% dimer (0:100) with the (a) B3LYP, (b) B3LYP-D3B(J), and (c) M06-2X-D3 functionals.

Figure S2: Simulated ROA and Raman of monomers and dimers combined in ratios of 100% monomer (100:0) to 100% dimer (0:100) with the (a) B3LYP, (b) B3LYP-D3B(J), and (c) M06-2X-D3 functionals.

Table S1: VA and VCD monomer-dimer analysis for the B3LYP, B3LYP-D3B(J), and M06-2X-D3 functionals.

Table S2: Raman and ROA monomer-dimer analysis for the B3LYP, B3LYP-D3B(J), and M06-2X-D3 functionals.

Figure S3: Comparison of the C=O stretching region of simulated B3LYP spectra of dimers (top), monomer (middle), and experiment (bottom).

Table S3: Comparison of the low-energy B3LYP dimer geometries that are subsequently optimized at the B3LYP-D3B(J) or M06-2X-D3 levels.

Figure S4: Comparison of VA, VCD, Raman, and ROA of the lowest-energy B3LYP conformer, C139, at the B3LYP, B3LYP-D3B(J), and M06-2X-D3 functionals.

Optimized geometries and energies: For 5LOH monomers and d5LOH dimers at all levels of theory.

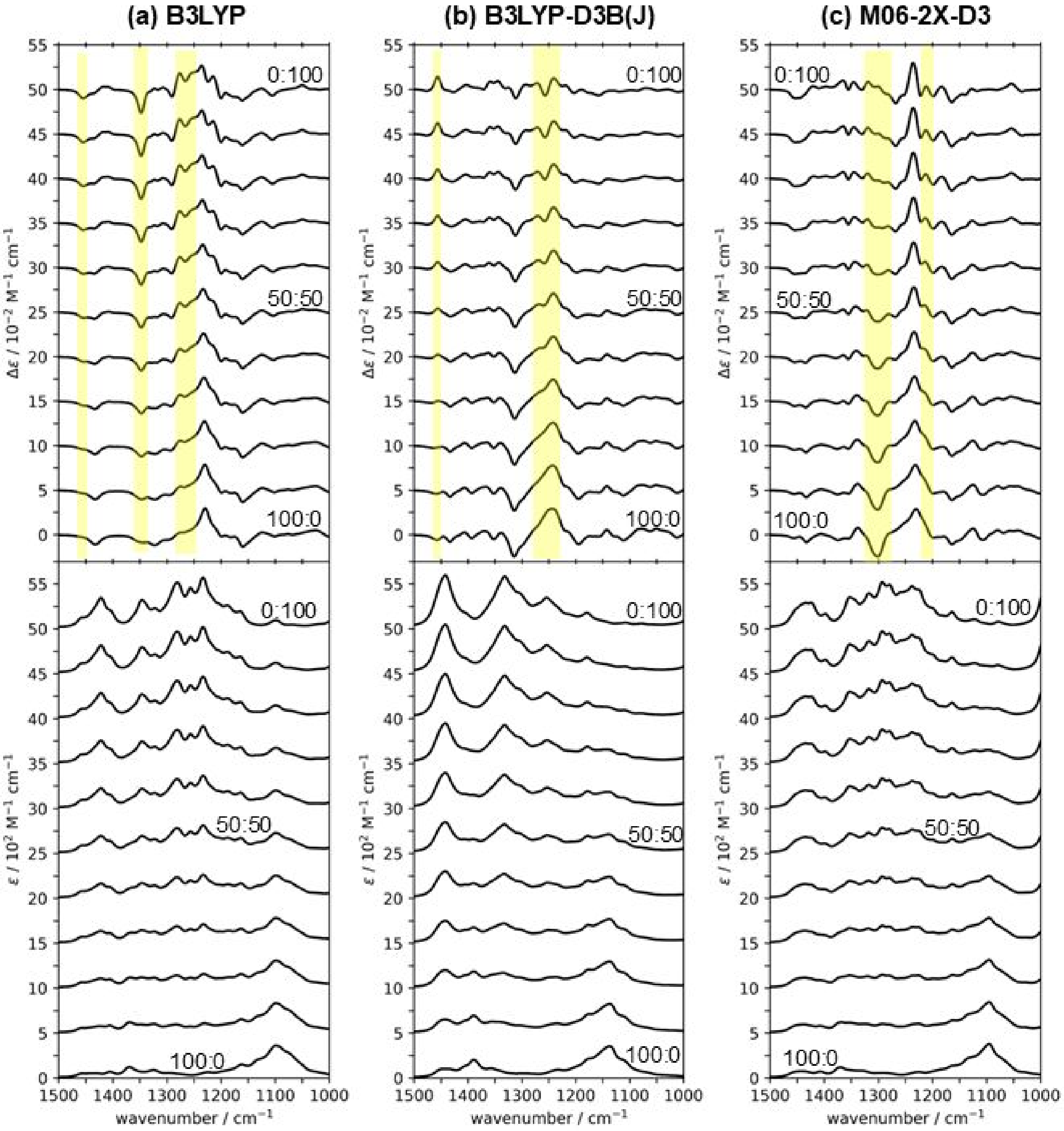

Fig. S1. Combined monomer and dimer VA (bottom) and VCD (top) spectra for B3LYP (a; left), B3LYP-D3B(J) (b; middle), and M06-2X-D3 (c; right). Combined ratios are in increments of 10%, from 100:0 (100% monomer, 0% dimer; bottom of each panel) and 0:100 (0% monomer, 100% dimer; top of each panel). Changes across the VCD spectra are highlighted in yellow for visualization purposes. All spectra are scaled by 0.97 for consistency.

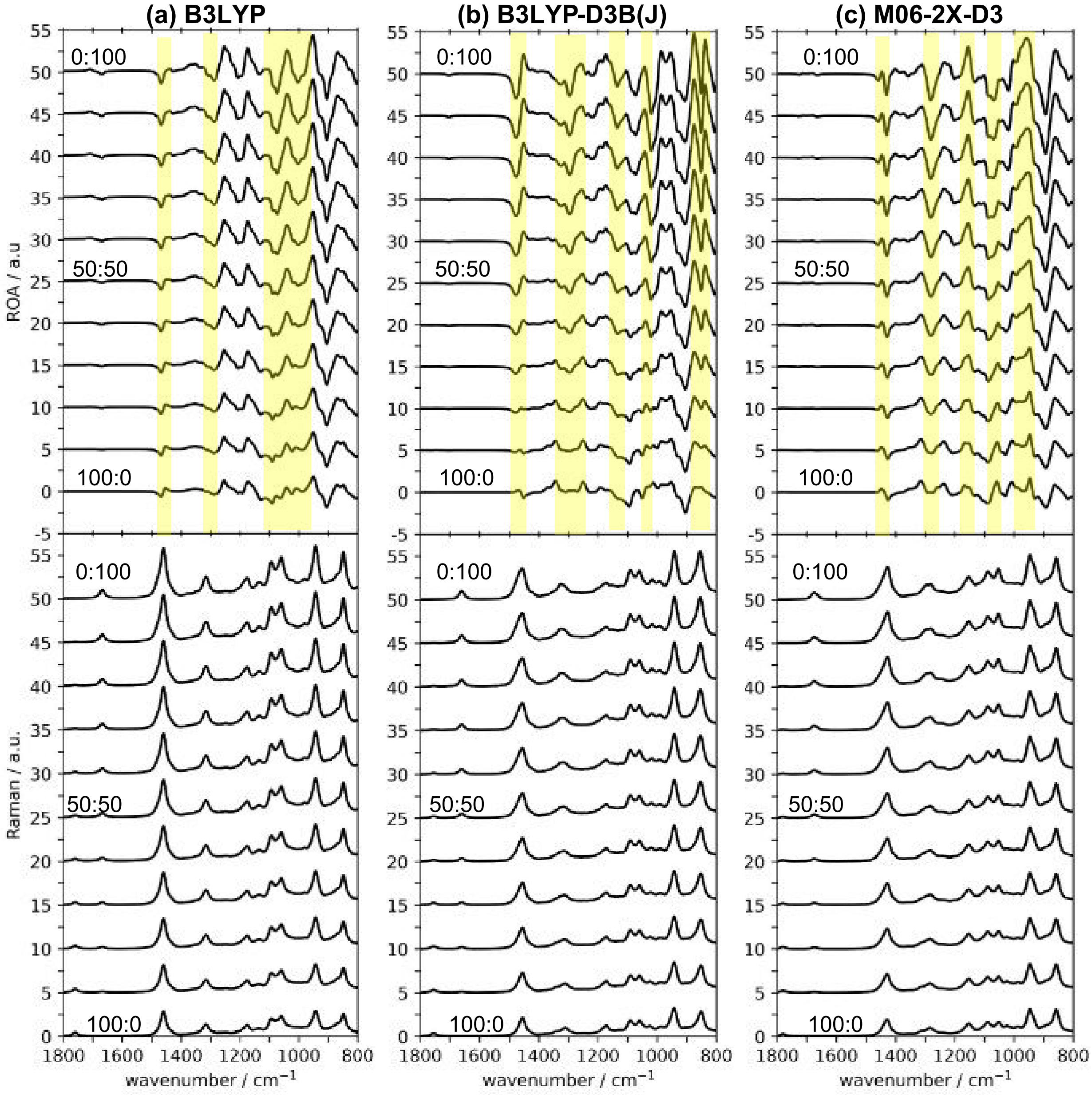

Fig. S2. Combined monomer and dimer Raman (bottom) and ROA (top) spectra for B3LYP (a; left), B3LYP-D3B(J) (b; middle), and M06-2X-D3 (c; right). Combined ratios are in increments of 10%, from 100:0 (100% monomer, 0% dimer; bottom of each panel) and 0:100 (0% monomer, 100% dimer; top of each panel). Changes across the ROA spectra are highlighted in yellow for visualization purposes. All spectra are scaled by 0.991 for consistency.

Table S1. Monomer:dimer analysis for the B3LYP, B3LYP-D3B(J), and M06-2X-D3 levels using VA and VCD spectra. The scale factor for each monomer:dimer ratio corresponds to the maximum SimVCD in the similarity overlap plot. Highlighted is the monomer:dimer ratio spectra that subsequently has the highest SimVA overlap and are reported in Table 4 of the manuscript. Values in parentheses are with universal scale factor as discussed in the section on Analysis Using a Universal Scale Factor.

	B3LYP			B3LYP-D3B(J)			M06-2X-D3			
	SF (0.980)	SimVA	SimVCD	SF (0.977)	SimVA	SimVCD	SF (0.968)	SimVA	SimVCD	
monomer	0.977	0.38 (0.39)	0.67 (0.62)	0.977	0.42 (0.42)	0.54 (0.54)	0.966	0.51 (0.53)	0.51 (0.50)	monomer
90:10	0.976	0.5 (0.51)	0.62 (0.57)	0.975	0.54(0.54)	0.54 (0.53)	0.967	0.62 (0.63)	0.50 (0.49)	90:10
80:20	0.975	0.62(0.62)	0.57(0.51)	0.974	0.64 (0.65)	0.51(0.47)	0.967	0.71 (0.72)	0.46 (0.46)	80:20
70:30	0.974	0.72 (0.72)	0.53 (0.46)	0.973	0.73 (0.73)	0.46(0.40)	0.967	0.77 (0.77)	0.41(0.40)	70:30
60:40	0.973	0.80(0.80)	0.50(0.43)	0.972	0.80 (0.80)	0.41(0.34)	0.967	0.81 (0.81)	0.35 (0.35)	60:40
50:50	0.972	0.85 (0.85)	0.48 (0.40)	0.971	0.85(0.83)	0.36 (0.29)	0.967	0.82 (0.82)	0.29 (0.29)	50:50
40:60	0.97	0.88(0.88)	0.47(0.38)	0.971	0.87 (0.85)	0.33(0.24)	0.968	0.82 (0.82)	0.25(0.25)	40:60
30:70	0.969	0.89 (0.88)	0.46 (0.37)	0.971	0.88 (0.86)	0.3 (0.21)	0.968	0.81 (0.81)	0.21(0.21)	30:70
20:80	0.967	0.89(0.88)	0.46 (0.36)	0.971	0.89(0.85)	0.28(0.18)	0.968	0.8 (0.8)	0.18(0.18)	20:80
10:90	0.966	0.88 (0.87)	0.46(0.34)	0.971	0.88 (0.84)	0.26(0.16)	0.968	0.78 (0.78)	0.15 (0.15)	10:90
dimer	0.966	0.87 (0.86)	0.46 (0.34)	0.97	0.87(0.83)	0.24 (0.14)	0.967	0.77 (0.76)	0.13 (0.13)	dimer

Table S2. Monomer:dimer analysis for the B3LYP, B3LYP-D3B(J), and M06-2X-D3 levels using the Raman and ROA spectra. The scale factor for each monomer:dimer ratio corresponds to the maximum *Sim*Raman in the similarity overlap plot. Highlighted is the monomer:dimer ratio spectra that subsequently has the highest *Sim*ROA overlap and are reported in Table 5 of the manuscript. Values in parentheses are with universal scale factor as discussed in the section on Analysis Using a Universal Scale Factor.

1800 to 800 cm^{-1}	B3LYP			B3LYP-D3B(J)			M06-2X-D3			
	SF (0.980)	SimRaman	SimROA	SF (0.977)	SimRaman	SimROA	SF (0.968)	SimRaman	SimROA	
monomer	0.987	0.66 (0.62)	0.37 (0.36)	0.984	0.69 (0.63)	0.28 (0.32)	0.973	0.63 (0.60)	0.19 (0.27)	monomer
90:10	0.987	0.67 (0.63)	0.32 (0.30)	0.984	0.68 (0.63)	0.33 (0.36)	0.974	0.64(0.61)	0.23(0.31)	90:10
80:20	0.987	0.67 (0.63)	0.28(0.25)	0.984	0.68(0.63)	0.35 (0.37)	0.974	0.64(0.61)	0.26 (0.33)	80:20
70:30	0.987	0.67 (0.63)	0.24(0.21)	0.983	0.68(0.63)	0.36(0.36)	0.974	0.64(0.61)	0.29(0.33)	70:30
60:40	0.987	0.67 (0.63)	0.21 (0.17)	0.983	0.67 (0.63)	0.34 (0.33)	0.974	0.64 (0.61)	0.30 (0.34)	60:40
50:50	0.987	0.68(0.63)	0.18(0.14)	0.983	0.67 (0.63)	0.32(0.31)	0.974	0.64 (0.61)	0.31 (0.33)	50:50
40:60	0.987	0.68(0.63)	0.15 (0.12)	0.983	0.67(0.63)	0.30(0.29)	0.974	0.64 (0.61)	0.32(0.33)	40:60
30:70	0.987	0.68(0.64)	0.13 (0.10)	0.983	0.67(0.63)	0.28 (0.27)	0.974	0.64 (0.61)	0.32 (0.33)	30:70
20:80	0.987	0.68(0.64)	0.12 (0.08)	0.983	0.66 (0.63)	0.26 (0.25)	0.974	0.64 (0.61)	0.32 (0.32)	20:80
10:90	0.987	0.68(0.64)	0.10 (0.06)	0.983	0.66 (0.63)	0.25 (0.23)	0.974	0.64 (0.61)	0.32 (0.32)	10:90
dimer	0.987	0.68 (0.64)	0.12 (0.07)	0.982	0.66 (0.63)	0.21 (0.19)	0.974	0.64 (0.61)	0.32 (0.31)	dimer

1200-800 cm-1

1200 to 800 cm^{-1}	B3LYP			B3LYP-D3B(J)			M06-2X-D3		
	SF (0.980)	SimRaman	SimROA	SF (0.977)	SimRaman	SimROA	SF (0.968)	SimRaman	SimROA

monomer	0.993	0.73 (0.57)	0.40 (0.40)	0.988	0.75 (0.63)	0.34 (0.48)	0.968	0.71	0.4	monomer
90:10	0.993	0.73 (0.57)	0.36 (0.34)	0.988	0.75(0.63)	0.37 (0.48)	0.968	0.7	0.44	90:10
80:20	0.993	0.73 (0.57)	0.32 (0.28)	0.988	0.74 (0.63)	0.39 (0.46)	0.968	0.7	0.46	80:20
70:30	0.993	0.73 (0.57)	0.29(0.23)	0.988	0.74 (0.62)	0.38 (0.42)	0.968	0.7	0.47	70:30
60:40	0.993	0.73 (0.57)	0.26 (0.19)	0.988	0.73 (0.62)	0.36 (0.38)	0.968	0.69	0.46	60:40
50:50	0.993	0.73 (0.57)	0.23 (0.16)	0.988	0.73 (0.62)	0.34 (0.34)	0.968	0.69	0.46	50:50
40:60	0.993	0.73 (0.57)	0.20 (0.13)	0.988	0.72 (0.62)	0.33 (0.30)	0.968	0.69	0.45	40:60
30:70	0.993	0.73 (0.57)	0.18 (0.11)	0.988	0.72 (0.62)	0.31(0.28)	0.968	0.69	0.44	30:70
20:80	0.993	0.73 (0.57)	0.17 (0.09)	0.988	0.72 (0.62)	0.29 (0.25)	0.968	0.68	0.43	20:80
10:90	0.993	0.73 (0.57)	0.15 (0.08)	0.988	0.72 (0.62)	0.28 (0.23)	0.968	0.68	0.42	10:90
dimer	0.993	0.73 (0.57)	0.17 (0.09)	0.988	0.71 (0.61)	0.23 (0.18)	0.968	0.68	0.41	dimer

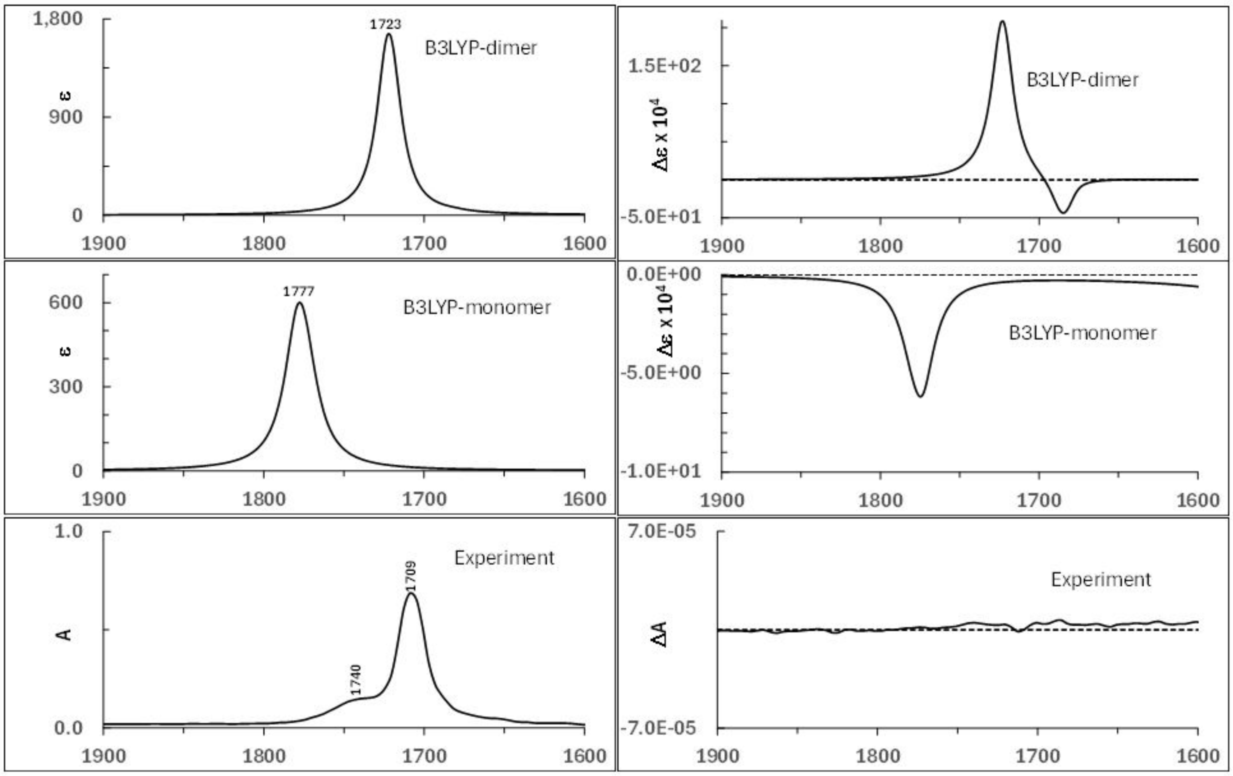

Fig. S3. Comparison of the C=O stretching region of simulated B3LYP spectra of dimers (top), monomer (middle), and experiment (bottom).

Table S3. Comparison of the low-energy B3LYP dimer geometries that are subsequently optimized at the B3LYP-D3B(J) or M06-2X-D3 levels. The lowest energy geometry in each column is the lowest-energy conformer at that level (low-energy B3LYP-D3B(J) and M06-2X-D3 geometries are not shown here). Energies reported in this table are electronic energies and not ZPEs, as used in the manuscript.

CREST Conformer number	B3LYP electronic energy (kcal/mol)	B3LYP then optimized with B3LYP-D3B(J) (kcal/mol)	B3LYP then optimized with M06-2X-D3 (kcal/mol)
2	1.40	4.85	2.80
4	1.46	3.54	9.06
8	1.44	3.99	2.69
10	1.70	4.76	4.66
11	1.45	4.68	4.01
15	1.38	5.32	4.64
17	1.20	7.31	5.82
57	1.27	4.67	9.25
58	0	8.84	7.34
114	1.16	6.70	6.02
139	0.03	7.61	5.41
144	1.86	5.29	3.42
158	1.06	4.56	2.91
168	1.18	7.10	5.69
223	1.02	9.01	8.92
246	1.41	6.12	5.01
295	1.92	6.21	9.94
355	1.70	7.45	5.98
396	1.80	12.45	10.00

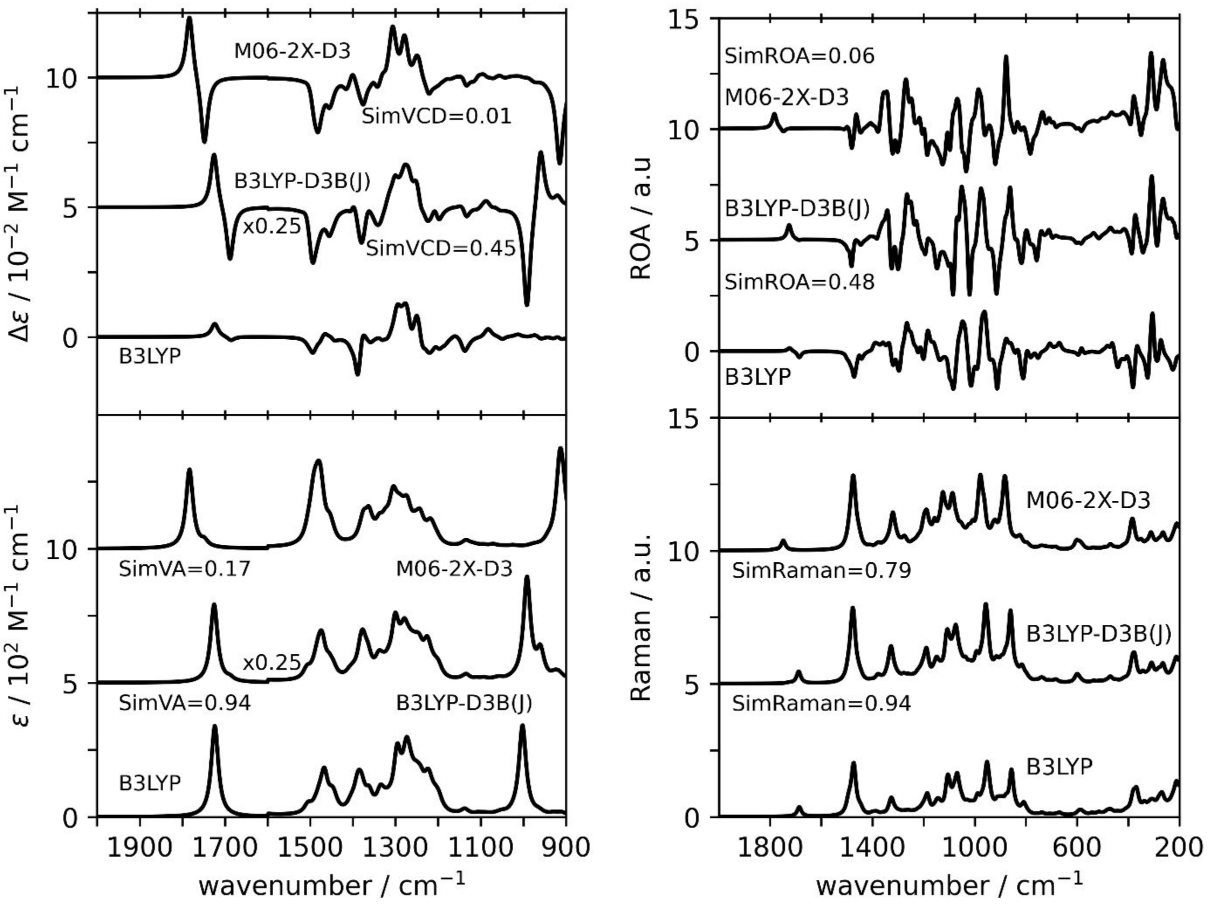

Fig. S4. Comparison of (left) VA, VCD, (right) Raman, and ROA of the lowest-energy B3LYP d5LOH conformer, C139, at the B3LYP, B3LYP-D3B(J), and M06-2X-D3 functionals. C139 is the lowest energy B3LYP conformer based on zero-point energy, as used in the manuscript. The optimized B3LYP geometry was subsequently optimized at the B3LYP-D3B(J) and M06-2X-D3 levels prior to VOA calculations. Spectra are presented unscaled. Due to intense bands in the C=O region, the 1600 to 2000 cm^{-1} region in the VA and VCD spectra is scaled by 0.25 for visualization purposes. *Sim* indices are indicated on the B3LYP-D3B(J) and M06-2X-D3 plots, which quantitatively compare these spectra to the B3LYP spectra.

Optimized geometries of monomers and dimers:

The following data are the geometries and energies of optimized conformers of monomers (5LOH) and dimers (d5LOH) of [5]-ladderanoic acid. To avoid listing all conformers, only those within 1.0 kcal/mol of the lowest energy conformer are listed here. Conformers are listed in terms of increasing zero-point energy and separated by method, with 5LOH conformers listed first then d5LOH. Gaussian 16 outputs of all conformers are available from the authors by reasonable request.

Naming convention: "VOA + [5LOH or d5LOH] + [method] + "C" + [conformer number from CREST conformational search]. Ex: VOA-d5LOH-B3PW91-D3BJ-C21

5LOH-B3LYP

VOA-5LOH-B3LYP-C4

__Requested operations__

Run with Gaussian 2016+B.01.

`freq=(VCD,ROA) CPHF=RdFreq B3LYP/6-31+G(2d,p) SCRF=(Solvent=chloroform) test`

__Relevant magnitudes__

Datum	Value
Charge	0
Multiplicity	1
Stoichiometry	C20H30O2
Number of Basis Functions	700
Electronic Energy (Eh)	-930.623562974
Sum of electronic and zero-point Energies (Eh)	-930.163148
Sum of electronic and thermal Energies (Eh)	-930.141086
Sum of electronic and enthalpy Energies (Eh)	-930.140142
Sum of electronic and thermal Free Energies (Eh)	-930.219245
Number of Imaginary Frequencies	0
Mean of alpha and beta Electrons	83

__Molecular Geometry in Cartesian Coordinates__

```xyz
C          1.433657        0.121467        0.223428
C          1.761588        1.564385       -0.280823
C          3.099012        1.069604       -0.883954
```

C	2.757219	-0.398989	-0.408170
C	4.294772	1.107881	0.089679
C	3.962408	-0.379875	0.549486
C	5.610056	0.619371	-0.543059
C	5.273648	-0.870535	-0.092255
C	6.806333	0.637730	0.428466
C	6.474806	-0.844868	0.872457
C	8.137364	0.141053	-0.195308
C	7.808928	-1.320309	0.238301
H	2.665979	-1.257982	-1.080747
H	3.307366	1.346134	-1.921216
H	5.201416	-1.728054	-0.769905
H	5.806869	0.931265	-1.574473
H	3.776296	-0.687099	1.584410
H	4.358868	1.970631	0.761432
H	6.282917	-1.147738	1.905625
H	6.868155	1.485348	1.116678
C	0.153874	-0.500993	-0.331730
C	-1.129252	0.118236	0.235999
C	-2.410879	-0.492302	-0.343535
C	-3.695094	0.112920	0.235202
C	-4.974588	-0.495865	-0.350095
C	-6.255514	0.110040	0.235012
C	-7.517418	-0.510012	-0.365231
C	-8.805617	0.052862	0.181674
O	-9.886600	-0.531690	-0.385869
O	-8.911833	0.918119	1.022131
H	1.428383	0.032341	1.317546
H	1.848428	2.343845	0.483693
H	1.054307	1.910921	-1.041930
H	9.024934	0.547167	0.298071
H	8.236990	0.314348	-1.272139
H	7.701433	-2.050580	-0.570819
H	8.517000	-1.723191	0.967962
H	0.151929	-0.405947	-1.428032
H	0.161455	-1.580095	-0.118842
H	-1.132413	-0.000750	1.329130
H	-1.128261	1.201078	0.047591
H	-2.408979	-1.577137	-0.163618
H	-2.410570	-0.365101	-1.435774
H	-3.698308	-0.018113	1.326751
H	-3.697055	1.198056	0.058607
H	-4.973403	-0.362190	-1.441443
H	-4.972359	-1.581488	-0.175921
H	-6.266001	-0.026415	1.323023
H	-6.265404	1.192917	0.062295
H	-7.542982	-0.380269	-1.454872

```
H         -7.541993       -1.595155       -0.201836
H        -10.679342       -0.128990        0.005380
```

VOA-5LOH-B3LYP-C1

__Requested operations__

Run with Gaussian 2016+B.01.

`freq=(VCD,ROA) CPHF=RdFreq B3LYP/6-31+G(2d,p) SCRF=(Solvent=chloroform) test`

__Relevant magnitudes__

Datum	Value
Charge	0
Multiplicity	1
Stoichiometry	C20H30O2
Number of Basis Functions	700
Electronic Energy (Eh)	-930.623551265
Sum of electronic and zero-point Energies (Eh)	-930.163052
Sum of electronic and thermal Energies (Eh)	-930.141036
Sum of electronic and enthalpy Energies (Eh)	-930.140092
Sum of electronic and thermal Free Energies (Eh)	-930.219346
Number of Imaginary Frequencies	0
Mean of alpha and beta Electrons	83

__Molecular Geometry in Cartesian Coordinates__

```xyz
C          1.656705       -1.825478       -0.522504
C          2.744649       -2.862602       -0.101664
C          3.526948       -1.764467        0.660163
C          2.413741       -0.713987        0.265213
C          4.555836       -0.998813       -0.196905
C          3.437968        0.069633       -0.575945
C          5.321199        0.080113        0.590005
C          4.198812        1.147245        0.218722
C          6.338866        0.860896       -0.264510
C          5.224431        1.924010       -0.629151
C          7.113024        1.956736        0.514691
C          6.012986        3.003176        0.159292
H          1.824420       -0.144218        0.990375
H          3.821378       -1.989135        1.689067
```

```
H          3.619679        1.725298        0.946874
H          5.627000       -0.176906        1.609938
H          3.142855        0.328687       -1.598522
H          5.131520       -1.585399       -0.920951
H          4.927998        2.176382       -1.651106
H          6.904168        0.286137       -1.003438
C          0.240559       -2.139813       -0.042451
C         -0.794424       -1.071334       -0.415726
C         -2.206417       -1.381990        0.095196
C         -3.242870       -0.318143       -0.285007
C         -4.653817       -0.627078        0.228979
C         -5.681780        0.446134       -0.147666
C         -7.081524        0.120376        0.373474
C         -8.124240        1.155908        0.033105
O         -9.342587        0.815275        0.514471
O         -7.943510        2.179025       -0.588965
H          1.633079       -1.631091       -1.602591
H          3.290587       -3.353453       -0.914663
H          2.344376       -3.638844        0.559361
H          8.087707        2.191801        0.077789
H          7.258213        1.751775        1.580733
H          5.474337        3.442603        1.005614
H          6.380502        3.818593       -0.470080
H         -0.067645       -3.107846       -0.464551
H          0.251276       -2.273943        1.049648
H         -0.474467       -0.097054       -0.020347
H         -0.820273       -0.962363       -1.509530
H         -2.527522       -2.357798       -0.297179
H         -2.180486       -1.487550        1.189428
H         -2.921161        0.657622        0.106320
H         -3.271076       -0.213202       -1.379019
H         -4.979440       -1.599857       -0.166763
H         -4.625436       -0.736865        1.322624
H         -5.364630        1.417746        0.249426
H         -5.717365        0.558424       -1.237901
H         -7.437582       -0.841056       -0.018466
H         -7.081557        0.006415        1.465232
H         -9.965575        1.517563        0.264740
```

VOA-5LOH-B3LYP-C6

__Requested operations__

Run with Gaussian 2016+B.01.

`freq=(VCD,ROA) CPHF=RdFreq B3LYP/6-31+G(2d,p) SCRF=(Solvent=chloroform) test`

__Relevant magnitudes__

Datum	Value
Charge	0
Multiplicity	1
Stoichiometry	C20H30O2
Number of Basis Functions	700
Electronic Energy (Eh)	-930.622882277
Sum of electronic and zero-point Energies (Eh)	-930.162216
Sum of electronic and thermal Energies (Eh)	-930.140318
Sum of electronic and enthalpy Energies (Eh)	-930.139374
Sum of electronic and thermal Free Energies (Eh)	-930.217924
Number of Imaginary Frequencies	0
Mean of alpha and beta Electrons	83

__Molecular Geometry in Cartesian Coordinates__

```xyz
C          -1.313488       -0.086736       -0.437870
C          -1.609538        1.429238       -0.678361
C          -2.886110        1.336767        0.192377
C          -2.570183       -0.186354        0.475081
C          -4.160116        0.930294       -0.577067
C          -3.854137       -0.600981       -0.265546
C          -5.412646        0.862562        0.314271
C          -5.100361       -0.665238        0.637671
C          -6.686275        0.436480       -0.442219
C          -6.379694       -1.080969       -0.113277
C          -7.955954        0.360914        0.446202
C          -7.650297       -1.132069        0.776365
H          -2.409382       -0.606195        1.473286
H          -3.010229        2.093138        0.972287
H          -4.960713       -1.076038        1.643391
H          -5.526188        1.650525        1.066627
H          -3.756053       -1.385384       -1.023990
H          -4.287874        1.351234       -1.580167
H          -6.274381       -1.863722       -0.869735
H          -6.812694        0.831924       -1.453937
C           0.018178       -0.404999        0.239413
C           1.236714       -0.181151       -0.664581
C           2.573655       -0.484521        0.022264
C           3.790877       -0.262982       -0.883212
C           5.127470       -0.572370       -0.199042
```

```
C         6.336903     -0.353564     -1.116698
C         7.680926     -0.679081     -0.458407
C         8.117767      0.315775      0.590893
O         9.069117     -0.196716      1.404773
O         7.717958      1.452918      0.708608
H        -1.407102     -0.697667     -1.345138
H        -0.843193      2.077684     -0.240345
H        -1.766903      1.740786     -1.716548
H        -8.885191      0.495832     -0.114585
H        -7.968114      1.051050      1.296556
H        -7.466593     -1.364421      1.830773
H        -8.413508     -1.821440      0.404385
H         0.004106     -1.451219      0.578739
H         0.118391      0.209261      1.146862
H         1.242080      0.858703     -1.020543
H         1.138795     -0.809659     -1.561632
H         2.570586     -1.525182      0.377709
H         2.671105      0.143504      0.919452
H         3.691472     -0.886860     -1.783371
H         3.797130      0.779101     -1.233312
H         5.123627     -1.615232      0.149773
H         5.228341      0.054022      0.696954
H         6.351142      0.683888     -1.470126
H         6.234578     -0.987367     -2.006123
H         8.483073     -0.690896     -1.208783
H         7.678944     -1.678048     -0.009014
H         9.335990      0.502967      2.023967
```

VOA-5LOH-B3LYP-C7

__Requested operations__

Run with Gaussian 2016+B.01.

`freq=(VCD,ROA) CPHF=RdFreq B3LYP/6-31+G(2d,p) SCRF=(Solvent=chloroform) test`

__Relevant magnitudes__

Datum	Value
Charge	0
Multiplicity	1
Stoichiometry	C20H30O2
Number of Basis Functions	700

Electronic Energy (Eh)	-930.622887266
Sum of electronic and zero-point Energies (Eh)	-930.162193
Sum of electronic and thermal Energies (Eh)	-930.140306
Sum of electronic and enthalpy Energies (Eh)	-930.139362
Sum of electronic and thermal Free Energies (Eh)	-930.217876
Number of Imaginary Frequencies	0
Mean of alpha and beta Electrons	83

__Molecular Geometry in Cartesian Coordinates__

```xyz
C          -1.301997       -0.285339        0.276753
C          -1.758629       -1.764906        0.062117
C          -3.046110       -1.283543       -0.650166
C          -2.570937        0.214067       -0.473406
C          -4.239050       -1.016798        0.291042
C          -3.773196        0.498039        0.444775
C          -5.505758       -0.553254       -0.449870
C          -5.034869        0.961420       -0.307578
C          -6.697028       -0.265147        0.484727
C          -6.232300        1.241718        0.620542
C          -7.978962        0.208364       -0.249969
C          -7.518887        1.692655       -0.120770
H          -2.399450        0.907195       -1.303287
H          -3.281115       -1.743521       -1.614030
H          -4.885204        1.651443       -1.144946
H          -5.732328       -1.051155       -1.398885
H          -3.560674        0.992538        1.398903
H          -4.379185       -1.715614        1.122750
H          -6.012666        1.732721        1.572734
H          -6.833631       -0.944136        1.331036
C           0.031921        0.098067       -0.361234
C           1.249517       -0.509120        0.346557
C           2.585911       -0.138635       -0.307628
C           3.804067       -0.736371        0.406138
C           5.139797       -0.359993       -0.245308
C           6.351480       -0.972954        0.467922
C           7.694281       -0.611472       -0.174093
C           8.116200        0.823906        0.034592
O           9.058660        1.206439       -0.857295
O           7.711964        1.569142        0.899490
H          -1.293261        0.019136        1.331390
H          -1.085788       -2.315709       -0.603930
H          -1.914225       -2.367219        0.963604
H          -8.898623       -0.006630        0.301524
H          -8.096025       -0.173911       -1.269626
H          -7.346943        2.227611       -1.060858
```

```
H          -8.186433        2.298881        0.498067
H           0.030162       -0.211886       -1.417113
H           0.122923        1.194186       -0.364791
H           1.150723       -1.603600        0.371089
H           1.255948       -0.180200        1.395776
H           2.684445        0.956110       -0.335644
H           2.580691       -0.470910       -1.355869
H           3.808164       -0.405774        1.454560
H           3.708342       -1.831629        0.431619
H           5.136630       -0.686743       -1.295298
H           5.237717        0.733423       -0.261224
H           6.258230       -2.065870        0.464382
H           6.360058       -0.661625        1.518774
H           7.697369       -0.821045       -1.249189
H           8.500860       -1.223406        0.252069
H           9.315595        2.118471       -0.642002
```

VOA-5LOH-B3LYP-C3

__Requested operations__

Run with Gaussian 2016+B.01.

`freq=(VCD,ROA) CPHF=RdFreq B3LYP/6-31+G(2d,p) SCRF=(Solvent=chloroform) test`

__Relevant magnitudes__

Datum	Value
Charge	0
Multiplicity	1
Stoichiometry	C20H30O2
Number of Basis Functions	700
Electronic Energy (Eh)	-930.622867187
Sum of electronic and zero-point Energies (Eh)	-930.162114
Sum of electronic and thermal Energies (Eh)	-930.140277
Sum of electronic and enthalpy Energies (Eh)	-930.139333
Sum of electronic and thermal Free Energies (Eh)	-930.217549
Number of Imaginary Frequencies	0
Mean of alpha and beta Electrons	83

__Molecular Geometry in Cartesian Coordinates__

```xyz
```

C	1.395131	1.644993	0.436495
C	2.354437	2.803585	0.019924
C	3.289920	1.799341	-0.696948
C	2.304677	0.624484	-0.312835
C	4.379248	1.182169	0.204481
C	3.393518	-0.014119	0.568208
C	5.299871	0.198862	-0.539696
C	4.308202	-0.996531	-0.187533
C	6.377822	-0.437486	0.359736
C	5.394707	-1.629491	0.702557
C	7.311243	-1.433931	-0.377080
C	6.339022	-2.607296	-0.046242
H	1.809922	-0.023502	-1.043048
H	3.586261	2.041849	-1.721242
H	3.829242	-1.650087	-0.924594
H	5.605833	0.479827	-1.553253
H	3.103266	-0.296508	1.586003
H	4.852025	1.847301	0.935106
H	5.098933	-1.908320	1.717807
H	6.839463	0.212266	1.108480
C	-0.038026	1.771498	-0.077538
C	-0.940397	0.589006	0.296027
C	-2.371345	0.718707	-0.239491
C	-3.276965	-0.458672	0.140305
C	-4.709558	-0.323367	-0.388460
C	-5.602213	-1.515110	-0.020412
C	-7.036124	-1.398224	-0.546110
C	-7.877636	-0.368248	0.170033
O	-8.954679	-0.000783	-0.561299
O	-7.670831	0.079134	1.276367
H	1.371791	1.466773	1.519363
H	2.810639	3.376309	0.834699
H	1.877538	3.509047	-0.669037
H	8.290967	-1.540575	0.097186
H	7.468731	-1.221907	-1.440008
H	5.889042	-3.116675	-0.905064
H	6.783431	-3.365854	0.604269
H	-0.473467	2.700309	0.319850
H	-0.020093	1.887870	-1.171572
H	-0.970842	0.491936	1.390819
H	-0.497211	-0.343826	-0.080171
H	-2.341347	0.814297	-1.334563
H	-2.815103	1.652161	0.135659
H	-2.836552	-1.392089	-0.239377
H	-3.304136	-0.557012	1.234873
H	-5.152379	0.602050	0.002821
H	-4.684307	-0.216950	-1.482646

```
H          -5.165307       -2.433956       -0.430276
H          -5.627685       -1.637431        1.068452
H          -7.056439       -1.178331       -1.618919
H          -7.567982       -2.351839       -0.425859
H          -9.473360        0.625038       -0.028825
```

VOA-5LOH-B3LYP-C2

__Requested operations__

Run with Gaussian 2016+B.01.

`freq=(VCD,ROA) CPHF=RdFreq B3LYP/6-31+G(2d,p) SCRF=(Solvent=chloroform) test`

__Relevant magnitudes__

Datum	Value
Charge	0
Multiplicity	1
Stoichiometry	C20H30O2
Number of Basis Functions	700
Electronic Energy (Eh)	-930.622865849
Sum of electronic and zero-point Energies (Eh)	-930.162083
Sum of electronic and thermal Energies (Eh)	-930.140259
Sum of electronic and enthalpy Energies (Eh)	-930.139315
Sum of electronic and thermal Free Energies (Eh)	-930.217562
Number of Imaginary Frequencies	0
Mean of alpha and beta Electrons	83

__Molecular Geometry in Cartesian Coordinates__

```xyz
C          -1.478628        1.848482       -0.315886
C          -2.459071        2.829573        0.399837
C          -3.243196        1.638730        1.003474
C          -2.231296        0.638007        0.315547
C          -4.381830        1.102410        0.111196
C          -3.366000        0.076459       -0.559937
C          -5.150100       -0.070917        0.744803
C          -4.126879       -1.097526        0.084914
C          -6.275743       -0.626031       -0.149919
C          -5.260153       -1.651495       -0.799494
C          -7.056062       -1.812269        0.475527
```

```
C          -6.051540      -2.822482      -0.158400
H          -1.617852      -0.084917       0.862160
H          -3.441157       1.669095       2.078483
H          -3.529019      -1.828782       0.639455
H          -5.361554      -0.000708       1.817267
H          -3.170930       0.004079      -1.635423
H          -4.973494       1.844778      -0.435245
H          -5.058967      -1.719852      -1.872263
H          -6.857841       0.104969      -0.718021
C          -0.008524       2.002175       0.070515
C           0.923124       0.989028      -0.606082
C           2.392675       1.141586      -0.195778
C           3.328554       0.140038      -0.882555
C           4.796798       0.293722      -0.469165
C           5.727693      -0.703165      -1.170828
C           7.200469      -0.556102      -0.776903
C           7.519053      -1.022299       0.624170
O           8.693087      -0.517236       1.067754
O           6.852443      -1.779126       1.294740
H          -1.564259       1.868193      -1.410093
H          -3.037914       3.499032      -0.245798
H          -1.955544       3.437324       1.159367
H          -8.072815      -1.914981       0.085856
H          -7.109666      -1.806177       1.569447
H          -5.478425      -3.437086       0.543956
H          -6.514136      -3.487676      -0.892983
H           0.317254       3.021559      -0.184621
H           0.088430       1.916670       1.163194
H           0.838209       1.095768      -1.697208
H           0.587531      -0.031238      -0.372882
H           2.478232       1.027705       0.894437
H           2.727488       2.164292      -0.422364
H           2.994797      -0.882602      -0.655964
H           3.244567       0.254196      -1.973099
H           5.131424       1.317170      -0.692368
H           4.879991       0.170570       0.618627
H           5.652923      -0.564778      -2.256411
H           5.399951      -1.727901      -0.961485
H           7.837456      -1.155203      -1.441683
H           7.545977       0.477699      -0.885060
H           8.859044      -0.887833       1.950554
```

VOA-5LOH-B3LYP-C23

__Requested operations__

Run with Gaussian 2016+B.01.

`freq=(VCD,ROA) CPHF=RdFreq B3LYP/6-31+G(2d,p) SCRF=(Solvent=chloroform) test`

__Relevant magnitudes__

Datum	Value
Charge	0
Multiplicity	1
Stoichiometry	C20H30O2
Number of Basis Functions	700
Electronic Energy (Eh)	-930.62286487
Sum of electronic and zero-point Energies (Eh)	-930.161952
Sum of electronic and thermal Energies (Eh)	-930.139971
Sum of electronic and enthalpy Energies (Eh)	-930.139026
Sum of electronic and thermal Free Energies (Eh)	-930.218135
Number of Imaginary Frequencies	0
Mean of alpha and beta Electrons	83

__Molecular Geometry in Cartesian Coordinates__

```xyz
C          -1.643177       -1.858025        0.459807
C          -2.742860       -2.869430        0.008031
C          -3.518226       -1.737765       -0.710667
C          -2.393600       -0.712306       -0.283854
C          -4.536351       -0.993340        0.177539
C          -3.406759        0.050360        0.589300
C          -5.294286        0.119889       -0.567629
C          -4.160298        1.162564       -0.163619
C          -6.301188        0.880086        0.317691
C          -5.175085        1.918690        0.715406
C          -7.066784        2.010203       -0.419973
C          -5.955823        3.032864       -0.030947
H          -1.802239       -0.122464       -0.991138
H          -3.819068       -1.923033       -1.745534
H          -3.578312        1.760305       -0.873381
H          -5.606409       -0.098053       -1.594706
H          -3.105057        0.270234        1.619104
H          -5.114768       -1.599774        0.882784
H          -4.872343        2.131595        1.744480
H          -6.869904        0.285429        1.038001
C          -0.232518       -2.166982       -0.039592
C           0.813326       -1.121658        0.367515
```

```
C           2.218164       -1.419348       -0.170099
C           3.265716       -0.381271        0.249581
C           4.668154       -0.674740       -0.296298
C           5.708424        0.367582        0.128785
C           7.113786        0.052538       -0.428783
C           8.126334        1.071645        0.023883
O           8.136938        2.172555       -0.761244
O           8.845861        0.967927        0.994102
H          -1.612078       -1.702667        1.545996
H          -2.353118       -3.625617       -0.681899
H          -3.289610       -3.383553        0.805945
H          -8.038185        2.238959        0.027441
H          -7.216338        1.844361       -1.492190
H          -5.415761        3.497865       -0.862581
H          -6.313844        3.828139        0.628949
H          -0.250394       -2.260046       -1.135850
H           0.069642       -3.152603        0.344321
H           0.496058       -0.129754        0.016208
H           0.850414       -1.058673        1.464591
H           2.535548       -2.414160        0.174358
H           2.181859       -1.474185       -1.267674
H           3.307216       -0.330948        1.346959
H           2.945984        0.614267       -0.090070
H           4.629673       -0.721812       -1.393697
H           4.991095       -1.668815        0.043353
H           5.396862        1.361188       -0.215719
H           5.759290        0.411946        1.223874
H           7.447246       -0.925069       -0.071698
H           7.082156        0.036630       -1.522613
H           8.780293        2.797128       -0.386938
```

VOA-5LOH-B3LYP-C24

__Requested operations__

Run with Gaussian 2016+B.01.

`freq=(VCD,ROA) CPHF=RdFreq B3LYP/6-31+G(2d,p) SCRF=(Solvent=chloroform) test`

__Relevant magnitudes__

Datum	Value
Charge	0

Multiplicity	1
Stoichiometry	C20H30O2
Number of Basis Functions	700
Electronic Energy (Eh)	-930.62286721
Sum of electronic and zero-point Energies (Eh)	-930.16193
Sum of electronic and thermal Energies (Eh)	-930.139961
Sum of electronic and enthalpy Energies (Eh)	-930.139017
Sum of electronic and thermal Free Energies (Eh)	-930.21805
Number of Imaginary Frequencies	0
Mean of alpha and beta Electrons	83

__Molecular Geometry in Cartesian Coordinates__

```xyz
C          -1.637572       -1.841652        0.478845
C          -2.735140       -2.864931        0.049196
C          -3.519828       -1.748114       -0.682499
C          -2.398141       -0.710786       -0.277093
C          -4.537085       -0.995575        0.199770
C          -3.410556        0.059588        0.590193
C          -5.304278        0.102761       -0.557863
C          -4.173446        1.156858       -0.175156
C          -6.310454        0.870989        0.321344
C          -5.187559        1.920988        0.697678
C          -7.085605        1.986178       -0.428925
C          -5.977658        3.019958       -0.061288
H          -1.813819       -0.128383       -0.996268
H          -3.824983       -1.950508       -1.712881
H          -3.598049        1.746822       -0.896680
H          -5.620504       -0.131884       -1.579991
H          -3.104620        0.296161        1.615044
H          -5.108896       -1.594318        0.916888
H          -4.880719        2.150847        1.721885
H          -6.872366        0.284220        1.053378
C          -0.228925       -2.151498       -0.025705
C           0.815459       -1.096807        0.360449
C           2.216665       -1.393992       -0.186831
C           3.264568       -0.349609        0.215973
C           4.661796       -0.640118       -0.344649
C           5.703312        0.407101        0.065025
C           7.100501        0.101229       -0.517604
C           8.104023        1.152554       -0.122019
O           8.635557        0.924213        1.100240
O           8.405034        2.124106       -0.781772
H          -1.599951       -1.670043        1.562394
H          -2.345906       -3.630166       -0.630967
H          -3.275049       -3.368797        0.858239
```

```
H          -8.055688         2.216838         0.020373
H          -7.240140         1.803628        -1.497713
H          -5.444223         3.474798        -0.902749
H          -6.336080         3.823477         0.588318
H          -0.253730        -2.259170        -1.120482
H           0.079855        -3.130743         0.369137
H           0.490466        -0.110295         0.001165
H           0.862038        -1.021636         1.456414
H           2.540211        -2.385256         0.162197
H           2.171069        -1.457086        -1.283583
H           3.318163        -0.293667         1.312597
H           2.937115         0.642919        -0.124983
H           4.610876        -0.691144        -1.441282
H           4.992198        -1.631938        -0.005305
H           5.382425         1.399252        -0.276313
H           5.769342         0.452984         1.158855
H           7.442972        -0.875587        -0.162224
H           7.054532         0.082559        -1.609381
H           9.238269         1.658835         1.303137
```

5LOH-B3LYP-D3B(J)

VOA-5LOH-B3LYP-D3BJ-C10

__Requested operations__

Run with Gaussian 2016+B.01.

`freq=(VCD,ROA) CPHF=RdFreq B3LYP/6-31+G(2d,p) EmpiricalDispersion=GD3BJ SCRF=(Solvent=chloroform) test`

__Relevant magnitudes__

Datum	Value
Charge	0
Multiplicity	1
Stoichiometry	C20H30O2
Number of Basis Functions	700
Electronic Energy (Eh)	-930.7197815599999
Sum of electronic and zero-point Energies (Eh)	-930.256808
Sum of electronic and thermal Energies (Eh)	-930.235582
Sum of electronic and enthalpy Energies (Eh)	-930.234638
Sum of electronic and thermal Free Energies (Eh)	-930.30867
Number of Imaginary Frequencies	0
Mean of alpha and beta Electrons	83

__Molecular Geometry in Cartesian Coordinates__

```xyz
C          1.537574      -2.288692       0.355848
C          0.722642      -3.616456       0.416655
C         -0.426618      -2.969690      -0.394111
C          0.398019      -1.626035      -0.467104
C         -1.493132      -2.269346       0.464909
C         -0.658837      -0.916908       0.397307
C         -2.609817      -1.619156      -0.369513
C         -1.780565      -0.261729      -0.420232
C         -3.682330      -0.926759       0.486507
C         -2.851504       0.416991       0.453016
C         -4.801258      -0.242170      -0.339507
C         -3.987839       1.086894      -0.360873
H          0.683138      -1.136200      -1.401789
H         -0.785850      -3.515650      -1.269906
H         -1.488199       0.259013      -1.337464
```

```
H          -2.955657       -2.173341       -1.247858
H          -0.295681       -0.363982        1.268373
H          -1.796145       -2.769744        1.389899
H          -2.494702        0.942066        1.340261
H          -3.984533       -1.436175        1.404883
C           2.886774       -2.391551       -0.353390
C           3.527301       -1.034149       -0.672216
C           3.920117       -0.209726        0.558785
C           4.337508        1.233061        0.234274
C           3.264215        2.066830       -0.485769
C           1.919574        2.110719        0.244642
C           0.876833        2.925181       -0.517576
C          -0.439043        3.041918        0.206998
O          -1.443663        3.397708       -0.624584
O          -0.617247        2.863153        1.391024
H           1.667308       -1.819036        1.336219
H           0.446869       -3.969819        1.415349
H           1.217994       -4.435308       -0.114526
H          -5.750461       -0.159448        0.196070
H          -4.996335       -0.699157       -1.314952
H          -3.692474        1.454662       -1.348706
H          -4.486105        1.899829        0.175981
H           3.567551       -2.996469        0.262675
H           2.747304       -2.945092       -1.292299
H           2.824681       -0.462548       -1.289148
H           4.416511       -1.188440       -1.297456
H           4.745564       -0.712013        1.079837
H           3.089247       -0.187046        1.271843
H           4.610349        1.737917        1.170360
H           5.244034        1.220442       -0.385280
H           3.639172        3.091115       -0.610859
H           3.110257        1.679295       -1.500768
H           2.051896        2.530541        1.247960
H           1.530610        1.097480        0.384728
H           1.231473        3.950486       -0.691440
H           0.687572        2.501973       -1.509680
H          -2.255799        3.462405       -0.095722
```

VOA-5LOH-B3LYP-D3BJ-C5

__Requested operations__

Run with Gaussian 2016+B.01.

`freq=(VCD,ROA) CPHF=RdFreq B3LYP/6-31+G(2d,p) EmpiricalDispersion=GD3BJ SCRF=(Solvent=chloroform) test`

__Relevant magnitudes__

Datum	Value
Charge	0
Multiplicity	1
Stoichiometry	C20H30O2
Number of Basis Functions	700
Electronic Energy (Eh)	-930.718688671
Sum of electronic and zero-point Energies (Eh)	-930.255734
Sum of electronic and thermal Energies (Eh)	-930.234519
Sum of electronic and enthalpy Energies (Eh)	-930.233574
Sum of electronic and thermal Free Energies (Eh)	-930.308678
Number of Imaginary Frequencies	0
Mean of alpha and beta Electrons	83

__Molecular Geometry in Cartesian Coordinates__

```xyz
C          -1.034693       -2.529403       -0.007009
C          -0.040428       -3.667994        0.373301
C           0.988897       -2.624557        0.867126
C          -0.049140       -1.473907        0.570393
C           1.920146       -2.060450       -0.225735
C           0.888307       -0.880306       -0.489274
C           2.936345       -1.044317        0.318045
C           1.892941        0.132908        0.079887
C           3.848149       -0.443608       -0.767567
C           2.816257        0.734251       -0.990648
C           4.877805        0.578447       -0.222718
C           3.856785        1.737072       -0.431083
H          -0.440647       -0.791903        1.326444
H           1.445119       -2.799034        1.844808
H           1.523196        0.805207        0.858220
H           3.404112       -1.277693        1.279788
H           0.450897       -0.645710       -1.464739
H           2.276837       -2.761104       -0.987451
H           2.359853        0.976634       -1.954049
H           4.187205       -1.120870       -1.555462
C          -2.412401       -2.584614        0.648980
C          -3.214197       -1.281743        0.507122
C          -3.541781       -0.894204       -0.939470
C          -4.161575        0.503658       -1.092046
```

```
C          -3.304978        1.653193       -0.532832
C          -1.862026        1.654735       -1.048525
C          -1.028603        2.834444       -0.550777
C          -0.715855        2.789518        0.923984
O          -0.115274        3.928477        1.340379
O          -0.932674        1.867210        1.676842
H          -1.148209       -2.423483       -1.090813
H          -0.423604       -4.295480        1.184700
H           0.292878       -4.320614       -0.440451
H           5.767725        0.673099       -0.850542
H           5.202117        0.400774        0.807685
H           4.187664        2.478118       -1.163476
H           3.540246        2.266751        0.473281
H          -2.978122       -3.426874        0.224817
H          -2.285626       -2.804527        1.718005
H          -4.149846       -1.367962        1.075387
H          -2.645628       -0.475409        0.982913
H          -4.227514       -1.637581       -1.367338
H          -2.633533       -0.943053       -1.549493
H          -5.141945        0.526319       -0.597880
H          -4.350059        0.685035       -2.158823
H          -3.782210        2.606577       -0.796328
H          -3.291647        1.608303        0.560801
H          -1.358757        0.730216       -0.756770
H          -1.865031        1.677553       -2.145344
H          -1.513722        3.794247       -0.763067
H          -0.059872        2.870738       -1.065586
H           0.081991        3.827480        2.286072
```

VOA-5LOH-B3LYP-D3BJ-C1

__Requested operations__

Run with Gaussian 2016+B.01.

`freq=(VCD,ROA) CPHF=RdFreq B3LYP/6-31+G(2d,p) EmpiricalDispersion=GD3BJ
SCRF=(Solvent=chloroform) test`

__Relevant magnitudes__

Datum	Value
Charge	0
Multiplicity	1

Stoichiometry	C20H30O2
Number of Basis Functions	700
Electronic Energy (Eh)	-930.717063736
Sum of electronic and zero-point Energies (Eh)	-930.255711
Sum of electronic and thermal Energies (Eh)	-930.233756
Sum of electronic and enthalpy Energies (Eh)	-930.232812
Sum of electronic and thermal Free Energies (Eh)	-930.311942
Number of Imaginary Frequencies	0
Mean of alpha and beta Electrons	83

__Molecular Geometry in Cartesian Coordinates__

```xyz
C          1.666260       -1.864282       -0.529450
C          2.763287       -2.875481       -0.079376
C          3.511176       -1.754684        0.680238
C          2.388892       -0.730055        0.250179
C          4.537900       -0.988542       -0.174064
C          3.409556        0.053004       -0.588909
C          5.268825        0.114181        0.606569
C          4.135945        1.154233        0.199183
C          6.284474        0.894543       -0.245757
C          5.159303        1.931368       -0.645797
C          7.024593        2.013926        0.529053
C          5.913441        3.034451        0.139365
H          1.778303       -0.157917        0.954390
H          3.793624       -1.956447        1.716413
H          3.535206        1.733715        0.907300
H          5.562068       -0.119675        1.634905
H          3.125708        0.288823       -1.619434
H          5.135010       -1.576628       -0.878100
H          4.874178        2.160537       -1.675663
H          6.869741        0.315947       -0.964854
C          0.255643       -2.186303       -0.051113
C         -0.773426       -1.119741       -0.433008
C         -2.180556       -1.415548        0.090608
C         -3.207967       -0.349857       -0.297195
C         -4.614097       -0.639876        0.232241
C         -5.631291        0.436760       -0.152274
C         -7.026722        0.132167        0.385819
C         -8.057626        1.173745        0.036834
O         -9.277227        0.855804        0.529046
O         -7.866156        2.185068       -0.600919
H          1.648454       -1.685394       -1.611696
H          3.333622       -3.361950       -0.877291
H          2.364522       -3.651420        0.581972
H          8.002158        2.260503        0.106413
```

```
H           7.151825        1.825486        1.599971
H           5.350489        3.472712        0.969755
H           6.277623        3.847632       -0.493950
H          -0.050419       -3.157325       -0.466267
H           0.266846       -2.310325        1.041811
H          -0.445093       -0.142839       -0.052423
H          -0.804639       -1.025191       -1.527601
H          -2.511069       -2.393672       -0.286830
H          -2.147799       -1.506272        1.185618
H          -2.874907        0.628227        0.077521
H          -3.244034       -0.260874       -1.392021
H          -4.951679       -1.614722       -0.147043
H          -4.577738       -0.733780        1.326765
H          -5.300915        1.410588        0.227141
H          -5.675293        0.532191       -1.243396
H          -7.394967       -0.831290        0.011580
H          -7.017412        0.035757        1.478881
H          -9.892004        1.562577        0.272235
```

VOA-5LOH-B3LYP-D3BJ-C4

__Requested operations__

Run with Gaussian 2016+B.01.

`freq=(VCD,ROA) CPHF=RdFreq B3LYP/6-31+G(2d,p) EmpiricalDispersion=GD3BJ SCRF=(Solvent=chloroform) test`

__Relevant magnitudes__

Datum	Value
Charge	0
Multiplicity	1
Stoichiometry	C20H30O2
Number of Basis Functions	700
Electronic Energy (Eh)	-930.7169487000001
Sum of electronic and zero-point Energies (Eh)	-930.255695
Sum of electronic and thermal Energies (Eh)	-930.23369
Sum of electronic and enthalpy Energies (Eh)	-930.232746
Sum of electronic and thermal Free Energies (Eh)	-930.311791
Number of Imaginary Frequencies	0
Mean of alpha and beta Electrons	83

__Molecular Geometry in Cartesian Coordinates__

```xyz
C           1.429514        0.080118        0.260675
C           1.730019        1.513555       -0.278859
C           3.061252        1.021427       -0.892802
C           2.749438       -0.437092       -0.371135
C           4.265648        1.106027        0.062293
C           3.961046       -0.369930        0.570899
C           5.575522        0.617026       -0.573735
C           5.267241       -0.861144       -0.072836
C           6.780080        0.683860        0.380894
C           6.476304       -0.787452        0.874837
C           8.106473        0.186808       -0.247480
C           7.805202       -1.263791        0.235264
H           2.664974       -1.317005       -1.015782
H           3.250993        1.271855       -1.939429
H           5.198767       -1.739750       -0.722051
H           5.754094        0.899054       -1.616195
H           3.791425       -0.647717        1.616108
H           4.326580        1.988899        0.706379
H           6.300608       -1.060395        1.918441
H           6.836668        1.553382        1.040542
C           0.157727       -0.561395       -0.280318
C          -1.121316        0.076351        0.266853
C          -2.400165       -0.536845       -0.307870
C          -3.679345        0.090502        0.250459
C          -4.956988       -0.515929       -0.333877
C          -6.232138        0.114273        0.230475
C          -7.493073       -0.499792       -0.371784
C          -8.775134        0.089993        0.155263
O          -9.860847       -0.492361       -0.404300
O          -8.872443        0.975072        0.975772
H           1.430287        0.012572        1.355719
H           1.823832        2.308622        0.467601
H           1.003292        1.834323       -1.032040
H           8.996013        0.619036        0.218296
H           8.183662        0.327965       -1.330375
H           7.691965       -2.018167       -0.549997
H           8.530325       -1.634908        0.964270
H           0.160054       -0.493399       -1.378279
H           0.164647       -1.633938       -0.039119
H          -1.131226       -0.019687        1.361794
H          -1.111377        1.154490        0.055611
H          -2.407859       -1.617345       -0.105731
H          -2.391967       -0.431399       -1.402028
H          -3.691882       -0.021303        1.343704
```

```
H          -3.669325        1.171878        0.054353
H          -4.945882       -0.401531       -1.426985
H          -4.967467       -1.597801       -0.140043
H          -6.253695       -0.005527        1.319888
H          -6.227182        1.193957        0.040981
H          -7.506197       -0.386068       -1.463122
H          -7.532185       -1.581375       -0.191374
H         -10.649468       -0.069824       -0.026201
```

VOA-5LOH-B3LYP-D3BJ-C164

__Requested operations__

Run with Gaussian 2016+B.01.

`freq=(VCD,ROA) CPHF=RdFreq B3LYP/6-31+G(2d,p) EmpiricalDispersion=GD3BJ SCRF=(Solvent=chloroform) test`

__Relevant magnitudes__

Datum	Value
Charge	0
Multiplicity	1
Stoichiometry	C20H30O2
Number of Basis Functions	700
Electronic Energy (Eh)	-930.718985511
Sum of electronic and zero-point Energies (Eh)	-930.255671
Sum of electronic and thermal Energies (Eh)	-930.234495
Sum of electronic and enthalpy Energies (Eh)	-930.233551
Sum of electronic and thermal Free Energies (Eh)	-930.307073
Number of Imaginary Frequencies	0
Mean of alpha and beta Electrons	83

__Molecular Geometry in Cartesian Coordinates__

```xyz
C           2.498219       -1.795831        0.215475
C           2.214453       -3.161824       -0.475874
C           0.845741       -2.648560       -0.967147
C           1.195977       -1.212849       -0.420241
C          -0.305907       -2.745747        0.059529
C          -0.007857       -1.252852        0.524077
C          -1.651230       -2.298221       -0.524729
```

```
C          -1.330164       -0.794850       -0.118902
C          -2.806402       -2.318538        0.494465
C          -2.507802       -0.811537        0.866694
C          -4.172156       -1.892830       -0.098273
C          -3.863449       -0.401270        0.231235
H           1.319677       -0.339067       -1.061254
H           0.573934       -2.857812       -2.004646
H          -1.278305        0.039473       -0.822747
H          -1.886176       -2.631981       -1.540217
H           0.162104       -0.943979        1.560531
H          -0.309485       -3.617272        0.721520
H          -2.306587       -0.458956        1.881074
H          -2.809094       -3.139574        1.215570
C           3.822183       -1.121902       -0.137927
C           4.197567        0.046963        0.787769
C           3.161056        1.174552        0.887198
C           3.017777        2.017836       -0.384621
C           1.926080        3.093062       -0.290162
C           0.507084        2.517220       -0.276624
C          -0.568441        3.615102       -0.165621
C          -1.943947        3.007361       -0.196061
O          -2.335539        2.562726        1.018071
O          -2.632858        2.872683       -1.184299
H           2.411531       -1.859584        1.307476
H           2.904697       -3.341164       -1.307352
H           2.205730       -4.049763        0.165275
H          -5.025171       -2.284716        0.461618
H          -4.315019       -2.132495       -1.156586
H          -4.571505        0.035176        0.942640
H          -3.779276        0.267021       -0.631243
H           3.794099       -0.795171       -1.186229
H           4.621534       -1.873211       -0.079426
H           5.159555        0.467716        0.464959
H           4.362859       -0.360266        1.794194
H           3.445229        1.842728        1.711020
H           2.192630        0.746836        1.169804
H           3.980607        2.501967       -0.593368
H           2.809437        1.373386       -1.247799
H           2.083942        3.695112        0.615543
H           2.018264        3.781474       -1.139871
H           0.385316        1.817266        0.555348
H           0.339582        1.946142       -1.197178
H          -0.488773        4.308606       -1.005866
H          -0.435136        4.170235        0.767596
H          -3.191705        2.116880        0.907873
```

VOA-5LOH-B3LYP-D3BJ-C924

__Requested operations__

Run with Gaussian 2016+B.01.

`freq=(VCD,ROA) CPHF=RdFreq B3LYP/6-31+G(2d,p) EmpiricalDispersion=GD3BJ SCRF=(Solvent=chloroform) test`

__Relevant magnitudes__

Datum	Value
Charge	0
Multiplicity	1
Stoichiometry	C20H30O2
Number of Basis Functions	700
Electronic Energy (Eh)	-930.718433376
Sum of electronic and zero-point Energies (Eh)	-930.255606
Sum of electronic and thermal Energies (Eh)	-930.234339
Sum of electronic and enthalpy Energies (Eh)	-930.233395
Sum of electronic and thermal Free Energies (Eh)	-930.307262
Number of Imaginary Frequencies	0
Mean of alpha and beta Electrons	83

__Molecular Geometry in Cartesian Coordinates__

```xyz
C          -3.208590       0.406625       0.505885
C          -3.550058       1.924818       0.382911
C          -2.265807       2.174097      -0.444378
C          -1.927317       0.637873      -0.344996
C          -1.015164       2.507648       0.390737
C          -0.677671       0.959031       0.487925
C           0.247731       2.723589      -0.460498
C           0.577494       1.170432      -0.369515
C           1.507042       3.027091       0.371369
C           1.832613       1.481850       0.460695
C           2.785037       3.231700      -0.482490
C           3.107521       1.709975      -0.388323
H          -1.790505      -0.004729      -1.215466
H          -2.381531       2.713144      -1.387837
H           0.700614       0.510341      -1.233319
H           0.126729       3.281226      -1.394526
H          -0.550142       0.413470       1.427141
```

```
H          -1.151991        3.170085        1.251022
H           1.952387        0.930077        1.395291
H           1.379950        3.677177        1.240424
C          -4.263933       -0.531063       -0.078600
C          -4.033698       -2.043552        0.096812
C          -2.777396       -2.616566       -0.586544
C          -1.503748       -2.522347        0.267034
C          -0.208189       -2.604261       -0.543166
C           1.039691       -2.362368        0.309866
C           2.307489       -2.254585       -0.532333
C           3.536838       -1.891445        0.258587
O           4.631915       -1.805144       -0.531230
O           3.587010       -1.686049        1.450551
H          -2.978747        0.097921        1.532989
H          -3.623249        2.482563        1.322188
H          -4.465768        2.096357       -0.192273
H           3.535524        3.861612        0.001980
H           2.606248        3.623128       -1.489057
H           3.135312        1.163745       -1.336773
H           4.031703        1.497911        0.155061
H          -5.227774       -0.279762        0.386056
H          -4.378716       -0.308228       -1.149863
H          -4.001535       -2.282904        1.169221
H          -4.919644       -2.554160       -0.300093
H          -2.950505       -3.668129       -0.846699
H          -2.621945       -2.096041       -1.540855
H          -1.502088       -1.581936        0.823824
H          -1.518293       -3.315475        1.026508
H          -0.136939       -3.577754       -1.047679
H          -0.245014       -1.850226       -1.341208
H           0.915199       -1.436817        0.880387
H           1.153904       -3.163851        1.048626
H           2.519388       -3.189048       -1.066082
H           2.193713       -1.491820       -1.312811
H           5.383002       -1.559587        0.033401
```

VOA-5LOH-B3LYP-D3BJ-C128

__Requested operations__

Run with Gaussian 2016+B.01.

`freq=(VCD,ROA) CPHF=RdFreq B3LYP/6-31+G(2d,p) EmpiricalDispersion=GD3BJ SCRF=(Solvent=chloroform) test`

__Relevant magnitudes__

Datum	Value
Charge	0
Multiplicity	1
Stoichiometry	C20H30O2
Number of Basis Functions	700
Electronic Energy (Eh)	-930.718503548
Sum of electronic and zero-point Energies (Eh)	-930.255475
Sum of electronic and thermal Energies (Eh)	-930.234297
Sum of electronic and enthalpy Energies (Eh)	-930.233352
Sum of electronic and thermal Free Energies (Eh)	-930.307044
Number of Imaginary Frequencies	0
Mean of alpha and beta Electrons	83

__Molecular Geometry in Cartesian Coordinates__

```xyz
C          -1.104632        2.606843        0.414637
C          -0.087315        3.773855        0.594648
C           0.974578        2.992455       -0.213816
C          -0.078797        1.847669       -0.475178
C           1.826635        2.011401        0.614956
C           0.776555        0.852450        0.322259
C           2.876351        1.269578       -0.229580
C           1.824640        0.111329       -0.518940
C           3.729745        0.283022        0.584098
C           2.677801       -0.864065        0.311523
C           4.773475       -0.490581       -0.262068
C           3.739803       -1.628705       -0.518446
H          -0.412575        1.542576       -1.469824
H           1.497549        3.535422       -1.004937
H           1.517846       -0.223951       -1.514091
H           3.391118        1.853069       -0.999470
H           0.262586        0.269251        1.093043
H           2.133982        2.337620        1.613340
H           2.161730       -1.419993        1.096259
H           4.039580        0.603804        1.581690
C          -2.443253        2.963172       -0.229509
C          -3.200284        1.754078       -0.803661
C          -3.304330        0.557170        0.144833
C          -3.975686       -0.656733       -0.501944
C          -3.935042       -1.931993        0.354148
C          -2.532044       -2.376886        0.797810
C          -1.554675       -2.542821       -0.364334
```

```
C          -0.195310      -3.038932       0.053138
O           0.626184      -3.193522      -1.009781
O           0.166096      -3.273875       1.184528
H          -1.288970       2.070153       1.350992
H           0.188899       4.029220       1.622850
H          -0.413089       4.687336       0.086780
H           5.643417      -0.819138       0.312180
H           5.129204       0.041173      -1.150305
H           3.469155      -1.804163      -1.564960
H           4.036750      -2.582685      -0.072690
H          -3.064736       3.475253       0.518365
H          -2.276823       3.687325      -1.038481
H          -4.204551       2.070433      -1.114160
H          -2.694063       1.420404      -1.719147
H          -2.296607       0.278086       0.467901
H          -3.846248       0.841219       1.058103
H          -5.021560      -0.418376      -0.734038
H          -3.494473      -0.851596      -1.469951
H          -4.546326      -1.785225       1.253754
H          -4.407558      -2.747783      -0.208843
H          -2.115069      -1.661720       1.513891
H          -2.612457      -3.326888       1.336348
H          -1.944288      -3.237022      -1.119402
H          -1.391609      -1.597441      -0.894701
H           1.491654      -3.481017      -0.675913
```

VOA-5LOH-B3LYP-D3BJ-C3

__Requested operations__

Run with Gaussian 2016+B.01.

`freq=(VCD,ROA) CPHF=RdFreq B3LYP/6-31+G(2d,p) EmpiricalDispersion=GD3BJ SCRF=(Solvent=chloroform) test`

__Relevant magnitudes__

Datum	Value
Charge	0
Multiplicity	1
Stoichiometry	C20H30O2
Number of Basis Functions	700
Electronic Energy (Eh)	-930.717017657

Sum of electronic and zero-point Energies (Eh)	-930.255426
Sum of electronic and thermal Energies (Eh)	-930.233637
Sum of electronic and enthalpy Energies (Eh)	-930.232693
Sum of electronic and thermal Free Energies (Eh)	-930.311189
Number of Imaginary Frequencies	0
Mean of alpha and beta Electrons	83

__Molecular Geometry in Cartesian Coordinates__

```xyz
C          1.389175        1.676896        0.449664
C          2.353433        2.817596        0.005284
C          3.263027        1.793957       -0.713603
C          2.273789        0.636679       -0.293781
C          4.357164        1.182978        0.181344
C          3.365507        0.005904        0.582975
C          5.250746        0.177628       -0.560186
C          4.253544       -0.998519       -0.168538
C          6.334357       -0.450818        0.333165
C          5.345116       -1.623725        0.715953
C          7.240229       -1.469993       -0.402832
C          6.262130       -2.624831       -0.031784
H          1.761624       -0.019687       -1.002975
H          3.546002        2.015452       -1.745601
H          3.755286       -1.660271       -0.884079
H          5.541778        0.435632       -1.583372
H          3.087323       -0.253358        1.609424
H          4.849567        1.855070        0.891185
H          5.062345       -1.878155        1.740527
H          6.814951        0.208743        1.060080
C         -0.040474        1.803066       -0.062221
C         -0.930118        0.617292        0.318323
C         -2.355500        0.726628       -0.227510
C         -3.246029       -0.456656        0.157830
C         -4.673080       -0.342107       -0.381894
C         -5.551883       -1.538561       -0.007318
C         -6.982097       -1.428773       -0.537271
C         -7.806060       -0.371440        0.155183
O         -8.869724        0.007895       -0.588411
O         -7.594493        0.089076        1.255053
H          1.370933        1.513319        1.534314
H          2.832761        3.389928        0.806101
H          1.872278        3.521544       -0.681354
H          8.227928       -1.580303        0.052523
H          7.375280       -1.277473       -1.472073
H          5.787650       -3.141546       -0.872279
H          6.710842       -3.376671        0.622724
```

```
H          -0.479890        2.731634        0.329667
H          -0.021713        1.911260       -1.156717
H          -0.965868        0.531063        1.413450
H          -0.473991       -0.313603       -0.045775
H          -2.318751        0.811579       -1.322886
H          -2.812970        1.657750        0.135675
H          -2.791480       -1.387768       -0.209607
H          -3.280161       -0.544070        1.252757
H          -5.129154        0.580967       -0.001876
H          -4.641851       -0.246161       -1.476501
H          -5.106727       -2.456226       -0.409538
H          -5.579324       -1.650765        1.082171
H          -6.999032       -1.233270       -1.614414
H          -7.522954       -2.373323       -0.392336
H          -9.376809        0.653739       -0.069047
```

VOA-5LOH-B3LYP-D3BJ-C182

__Requested operations__

Run with Gaussian 2016+B.01.

`freq=(VCD,ROA) CPHF=RdFreq B3LYP/6-31+G(2d,p) EmpiricalDispersion=GD3BJ SCRF=(Solvent=chloroform) test`

__Relevant magnitudes__

Datum	Value
Charge	0
Multiplicity	1
Stoichiometry	C20H30O2
Number of Basis Functions	700
Electronic Energy (Eh)	-930.7181461109999
Sum of electronic and zero-point Energies (Eh)	-930.255426
Sum of electronic and thermal Energies (Eh)	-930.234181
Sum of electronic and enthalpy Energies (Eh)	-930.233237
Sum of electronic and thermal Free Energies (Eh)	-930.307935
Number of Imaginary Frequencies	0
Mean of alpha and beta Electrons	83

__Molecular Geometry in Cartesian Coordinates__

```xyz
```

C	-1.430078	-2.445481	-0.226951
C	-0.559437	-3.733230	-0.334983
C	0.612358	-3.020343	0.379526
C	-0.285936	-1.733592	0.548672
C	1.553115	-2.236034	-0.554973
C	0.658868	-0.934421	-0.359740
C	2.707684	-1.552445	0.195050
C	1.810294	-0.253510	0.392930
C	3.647801	-0.756646	-0.725883
C	2.754012	0.532343	-0.533851
C	4.807489	-0.049414	0.020395
C	3.927000	1.222995	0.207289
H	-0.545385	-1.291935	1.513964
H	1.086558	-3.549802	1.209573
H	1.567163	0.201674	1.357030
H	3.173065	-2.119693	1.007275
H	0.196314	-0.365881	-1.172299
H	1.790686	-2.692685	-1.520998
H	2.290420	1.086534	-1.352461
H	3.880429	-1.201705	-1.696490
C	-2.746217	-2.595041	0.530561
C	-3.442299	-1.253587	0.796382
C	-4.003241	-0.559603	-0.460833
C	-3.927362	0.971918	-0.385475
C	-2.500578	1.499371	-0.573027
C	-2.380636	3.008969	-0.355045
C	-0.968991	3.545938	-0.589759
C	0.037179	3.116832	0.449531
O	1.298563	3.427499	0.076126
O	-0.207426	2.580463	1.506761
H	-1.614467	-1.974839	-1.198406
H	-0.972513	-4.561073	0.250396
H	-0.347361	-4.096852	-1.345764
H	5.686073	0.125024	-0.605951
H	5.133687	-0.545983	0.939649
H	3.716946	1.503974	1.245793
H	4.318871	2.099192	-0.316066
H	-3.413522	-3.263772	-0.032357
H	-2.545351	-3.093896	1.488618
H	-2.723586	-0.589213	1.290901
H	-4.255087	-1.398736	1.517893
H	-5.041651	-0.875573	-0.615517
H	-3.453328	-0.887832	-1.351767
H	-4.576045	1.417383	-1.150988
H	-4.318445	1.309066	0.585003
H	-1.819002	0.985192	0.112374
H	-2.161484	1.251181	-1.588858

```
H          -2.698306        3.259233        0.663535
H          -3.062127        3.533069       -1.035896
H          -0.960769        4.643382       -0.588405
H          -0.585655        3.248140       -1.572453
H           1.898630        3.132621        0.780679
```

VOA-5LOH-B3LYP-D3BJ-C834

__Requested operations__

Run with Gaussian 2016+B.01.

`freq=(VCD,ROA) CPHF=RdFreq B3LYP/6-31+G(2d,p) EmpiricalDispersion=GD3BJ SCRF=(Solvent=chloroform) test`

__Relevant magnitudes__

Datum	Value
Charge	0
Multiplicity	1
Stoichiometry	C20H30O2
Number of Basis Functions	700
Electronic Energy (Eh)	-930.718340275
Sum of electronic and zero-point Energies (Eh)	-930.255401
Sum of electronic and thermal Energies (Eh)	-930.234149
Sum of electronic and enthalpy Energies (Eh)	-930.233205
Sum of electronic and thermal Free Energies (Eh)	-930.307317
Number of Imaginary Frequencies	0
Mean of alpha and beta Electrons	83

__Molecular Geometry in Cartesian Coordinates__

```xyz
C           2.860216       -0.942850        0.349302
C           2.929270       -2.485853        0.570376
C           1.619706       -2.678472       -0.232129
C           1.582250       -1.127212       -0.514342
C           0.328443       -2.554456        0.600855
C           0.286974       -0.995841        0.297511
C          -0.953901       -2.744208       -0.227545
C          -0.987930       -1.183982       -0.534571
C          -2.244541       -2.597926        0.597189
C          -2.271689       -1.044559        0.301296
```

```
C          -3.541466       -2.763075       -0.237298
C          -3.577482       -1.228961       -0.509176
H           1.594210       -0.692686       -1.516830
H           1.622422       -3.442802       -1.013049
H          -0.995123       -0.748218       -1.538853
H          -0.940160       -3.529763       -0.989491
H           0.263864       -0.220983        1.066998
H           0.338630       -2.992498        1.603654
H          -2.270048       -0.274433        1.075813
H          -2.241550       -3.028701        1.601365
C           4.065506       -0.319989       -0.349464
C           3.876863        1.098997       -0.913293
C           3.652263        2.242542        0.093468
C           2.260439        2.366264        0.738905
C           1.098709        2.452974       -0.254526
C          -0.255272        2.647965        0.433347
C          -1.417202        2.526529       -0.548854
C          -2.776519        2.559014        0.097749
O          -3.767381        2.477353       -0.819252
O          -3.003880        2.635807        1.284617
H           2.650472       -0.400883        1.275103
H           3.796872       -2.935674        0.076889
H           2.904497       -2.831281        1.608952
H          -4.393936       -3.119156        0.346773
H          -3.438507       -3.403398       -1.119197
H          -3.518332       -0.919853       -1.557524
H          -4.439022       -0.732855       -0.054903
H           4.353807       -0.973744       -1.184941
H           4.916520       -0.324472        0.347012
H           4.783740        1.339374       -1.482289
H           3.065685        1.091543       -1.650894
H           4.404779        2.167915        0.890372
H           3.859692        3.186431       -0.428796
H           2.077466        1.529748        1.421308
H           2.257588        3.267779        1.366253
H           1.057299        1.537647       -0.854593
H           1.276764        3.274278       -0.963006
H          -0.290272        3.623819        0.931084
H          -0.377722        1.899612        1.223300
H          -1.355924        1.584527       -1.107623
H          -1.388657        3.318012       -1.307349
H          -4.612779        2.484024       -0.341259
```

VOA-5LOH-B3LYP-D3BJ-C2

__Requested operations__

Run with Gaussian 2016+B.01.

`freq=(VCD,ROA) CPHF=RdFreq B3LYP/6-31+G(2d,p) EmpiricalDispersion=GD3BJ SCRF=(Solvent=chloroform) test`

__Relevant magnitudes__

Datum	Value
Charge	0
Multiplicity	1
Stoichiometry	C20H30O2
Number of Basis Functions	700
Electronic Energy (Eh)	-930.717019564
Sum of electronic and zero-point Energies (Eh)	-930.255395
Sum of electronic and thermal Energies (Eh)	-930.233634
Sum of electronic and enthalpy Energies (Eh)	-930.23269
Sum of electronic and thermal Free Energies (Eh)	-930.310891
Number of Imaginary Frequencies	0
Mean of alpha and beta Electrons	83

__Molecular Geometry in Cartesian Coordinates__

```xyz
C          -1.475944       1.888034       -0.316733
C          -2.459551       2.840599        0.427226
C          -3.212417       1.628130        1.022735
C          -2.197567       0.656920        0.300945
C          -4.352905       1.094387        0.135941
C          -3.332648       0.098557       -0.569431
C          -5.090510      -0.100712        0.757531
C          -4.062478      -1.097051        0.063555
C          -6.217792      -0.652736       -0.132377
C          -5.197119      -1.649090       -0.815220
C          -6.967922      -1.860685        0.483382
C          -5.957609      -2.842361       -0.182637
H          -1.564925      -0.066919        0.822333
H          -3.396023       1.633854        2.099935
H          -3.445108      -1.828400        0.594629
H          -5.287415      -0.055165        1.833313
H          -3.151466       0.050629       -1.647920
H          -4.963528       1.837430       -0.386669
H          -5.009315      -1.693315       -1.890978
H          -6.817265       0.080922       -0.677174
```

```
C          -0.009309        2.045815        0.064989
C           0.910553        1.033065       -0.621062
C           2.377782        1.167126       -0.207744
C           3.299271        0.161710       -0.901833
C           4.765086        0.295234       -0.484141
C           5.681973       -0.707872       -1.189339
C           7.148421       -0.583595       -0.774544
C           7.421034       -1.041211        0.637041
O           8.586413       -0.546463        1.111901
O           6.723869       -1.783395        1.292506
H          -1.570228        1.924366       -1.409188
H          -3.062034        3.505840       -0.199818
H          -1.953323        3.446243        1.185823
H          -7.988441       -1.974381        0.108272
H          -7.001629       -1.872453        1.577599
H          -5.361395       -3.455343        0.500957
H          -6.420568       -3.503671       -0.919740
H           0.317364        3.065618       -0.184685
H           0.090128        1.951065        1.156321
H           0.826969        1.151619       -1.710656
H           0.564060        0.014638       -0.398015
H           2.460531        1.042273        0.881121
H           2.724407        2.187460       -0.425207
H           2.953146       -0.858316       -0.683914
H           3.218199        0.286394       -1.991063
H           5.113375        1.315429       -0.699183
H           4.844072        0.162203        0.602291
H           5.618708       -0.558126       -2.273623
H           5.336716       -1.728366       -0.989801
H           7.788041       -1.200504       -1.419433
H           7.512384        0.443422       -0.882949
H           8.720222       -0.911173        2.002450
```

VOA-5LOH-B3LYP-D3BJ-C7

__Requested operations__

Run with Gaussian 2016+B.01.

`freq=(VCD,ROA) CPHF=RdFreq B3LYP/6-31+G(2d,p) EmpiricalDispersion=GD3BJ SCRF=(Solvent=chloroform) test`

__Relevant magnitudes__

Datum	Value
Charge	0
Multiplicity	1
Stoichiometry	C20H30O2
Number of Basis Functions	700
Electronic Energy (Eh)	-930.716919623
Sum of electronic and zero-point Energies (Eh)	-930.255359
Sum of electronic and thermal Energies (Eh)	-930.233532
Sum of electronic and enthalpy Energies (Eh)	-930.232588
Sum of electronic and thermal Free Energies (Eh)	-930.311183
Number of Imaginary Frequencies	0
Mean of alpha and beta Electrons	83

__Molecular Geometry in Cartesian Coordinates__

```xyz
C          -1.289712       -0.249411        0.301885
C          -1.727460       -1.728401        0.063244
C          -3.007839       -1.250184       -0.659741
C          -2.554334        0.248499       -0.448017
C          -4.210449       -1.018176        0.273344
C          -3.763960        0.496301        0.465552
C          -5.470470       -0.552968       -0.471554
C          -5.019332        0.961673       -0.289343
C          -6.671227       -0.301620        0.456919
C          -6.225919        1.205634        0.632478
C          -7.947138        0.173313       -0.283096
C          -7.505973        1.658277       -0.114940
H          -2.385581        0.961766       -1.260236
H          -3.226371       -1.693990       -1.634147
H          -4.868905        1.672096       -1.108330
H          -5.680777       -1.030969       -1.433571
H          -3.565619        0.971075        1.431786
H          -4.351987       -1.736141        1.087312
H          -6.020628        1.676404        1.597209
H          -6.807200       -1.001943        1.284855
C           0.038300        0.144267       -0.332867
C           1.249293       -0.485969        0.358768
C           2.582876       -0.118646       -0.295708
C           3.794095       -0.742410        0.401143
C           5.126992       -0.367323       -0.249705
C           6.332740       -1.004970        0.445783
C           7.669753       -0.627417       -0.193011
C           8.064840        0.811158        0.032252
O           8.985902        1.228611       -0.865317
O           7.654563        1.532668        0.914078
```

```
H          -1.284320        0.042380        1.359473
H          -1.037558       -2.263316       -0.597276
H          -1.893082       -2.341205        0.955020
H          -8.871564       -0.063633        0.250150
H          -8.042995       -0.187311       -1.312329
H          -7.324546        2.213843       -1.040660
H          -8.188847        2.243180        0.506805
H           0.030066       -0.146517       -1.393796
H           0.135081        1.239187       -0.315281
H           1.138509       -1.579125        0.364910
H           1.263579       -0.174935        1.412928
H           2.693906        0.974851       -0.304806
H           2.568387       -0.432754       -1.349070
H           3.806786       -0.430870        1.454926
H           3.686477       -1.836478        0.406572
H           5.115788       -0.673437       -1.305428
H           5.236216        0.724634       -0.243833
H           6.234364       -2.096513        0.415699
H           6.344943       -0.716866        1.502755
H           7.671966       -0.824005       -1.270126
H           8.484757       -1.231937        0.226432
H           9.224970        2.142542       -0.638350
```

VOA-5LOH-B3LYP-D3BJ-C6

__Requested operations__

Run with Gaussian 2016+B.01.

`freq=(VCD,ROA) CPHF=RdFreq B3LYP/6-31+G(2d,p) EmpiricalDispersion=GD3BJ
SCRF=(Solvent=chloroform) test`

__Relevant magnitudes__

Datum	Value
Charge	0
Multiplicity	1
Stoichiometry	C20H30O2
Number of Basis Functions	700
Electronic Energy (Eh)	-930.71692163
Sum of electronic and zero-point Energies (Eh)	-930.255354
Sum of electronic and thermal Energies (Eh)	-930.233529
Sum of electronic and enthalpy Energies (Eh)	-930.232585

Sum of electronic and thermal Free Energies (Eh)	-930.311065
Number of Imaginary Frequencies	0
Mean of alpha and beta Electrons	83

__Molecular Geometry in Cartesian Coordinates__

```xyz
C          -1.300140       -0.247764       -0.431971
C          -1.565533        1.197958       -0.957006
C          -2.825918        1.307472       -0.067583
C          -2.548787       -0.146914        0.484171
C          -4.117178        0.807138       -0.739914
C          -3.844934       -0.651842       -0.167851
C          -5.354288        0.930466        0.162213
C          -5.078218       -0.525660        0.740417
C          -6.645481        0.417692       -0.498516
C          -6.372836       -1.028680        0.079860
C          -7.899344        0.530161        0.405141
C          -7.629116       -0.893777        0.977309
H          -2.394359       -0.385999        1.540557
H          -2.916846        2.196117        0.561819
H          -4.938064       -0.761366        1.800082
H          -5.435574        1.838373        0.768207
H          -3.772020       -1.558180       -0.777483
H          -4.250080        1.048408       -1.799237
H          -6.293953       -1.929981       -0.533279
H          -6.775710        0.639535       -1.560703
C           0.023867       -0.437754        0.297876
C           1.239212       -0.391259       -0.631008
C           2.572623       -0.536778        0.105566
C           3.788143       -0.500837       -0.823370
C           5.120151       -0.632255       -0.082172
C           6.331546       -0.605167       -1.018090
C           7.667133       -0.721013       -0.282806
C           8.023037        0.502119        0.525703
O           8.956905        0.231759        1.465464
O           7.573509        1.615612        0.368911
H          -1.402202       -1.022235       -1.202393
H          -0.774097        1.896629       -0.667698
H          -1.740677        1.304381       -2.032263
H          -8.834732        0.597806       -0.156573
H          -7.873109        1.352415        1.127470
H          -7.433204       -0.950871        2.052903
H          -8.416247       -1.612378        0.734276
H           0.006452       -1.399077        0.830976
H           0.123447        0.339714        1.069712
H           1.236627        0.555287       -1.188832
```

```
H            1.148540       -1.188449       -1.382447
H            2.574961       -1.479994        0.670232
H            2.663402        0.265498        0.851365
H            3.702611       -1.307429       -1.565410
H            3.782820        0.439782       -1.391660
H            5.127419       -1.570513        0.490569
H            5.206383        0.180032        0.650575
H            6.323634        0.318945       -1.606629
H            6.259361       -1.435281       -1.730680
H            8.491464       -0.861923       -0.994231
H            7.688533       -1.592839        0.379373
H            9.168275        1.064785        1.918580
```

5LOH-M06-2X-D3

VOA-5LOH-M06-2X-D3-C10

__Requested operations__

Run with Gaussian 2016+B.01.

`freq=(VCD,ROA) CPHF=RdFreq M062X/6-31+G(2d,p) EmpiricalDispersion=GD3 SCRF=(Solvent=chloroform) test`

__Relevant magnitudes__

Datum	Value
Charge	0
Multiplicity	1
Stoichiometry	C20H30O2
Number of Basis Functions	700
Electronic Energy (Eh)	-930.214475378
Sum of electronic and zero-point Energies (Eh)	-929.747615
Sum of electronic and thermal Energies (Eh)	-929.726872
Sum of electronic and enthalpy Energies (Eh)	-929.725927
Sum of electronic and thermal Free Energies (Eh)	-929.797198
Number of Imaginary Frequencies	0
Mean of alpha and beta Electrons	83

__Molecular Geometry in Cartesian Coordinates__

```xyz
C          1.552598       -2.101528        0.450462
C          0.734030       -3.409733        0.627482
C         -0.375456       -2.861894       -0.299909
C          0.401955       -1.497854       -0.386287
C         -1.510596       -2.175989        0.460687
C         -0.695829       -0.818893        0.452713
C         -2.563146       -1.543864       -0.461542
C         -1.779321       -0.167410       -0.408265
C         -3.722451       -0.912037        0.312001
C         -2.925683        0.440116        0.416698
C         -4.762296       -0.214816       -0.593769
C         -4.001858        1.130768       -0.450187
H          0.668572       -1.004200       -1.326048
H         -0.646231       -3.468718       -1.166323
H         -1.451161        0.400382       -1.285858
```

```
H          -2.808544       -2.089503       -1.377708
H          -0.373336       -0.274340        1.345255
H          -1.884671       -2.674274        1.360048
H          -2.644289        0.913929        1.359394
H          -4.103349       -1.464413        1.173393
C           2.852589       -2.304128       -0.323338
C           3.522700       -0.998199       -0.753285
C           4.062154       -0.158007        0.405971
C           4.395349        1.285059        0.011668
C           3.219855        2.052073       -0.609012
C           1.934175        1.972311        0.211954
C           0.788530        2.719191       -0.455635
C          -0.461103        2.753538        0.381615
O          -1.522676        3.191813       -0.317323
O          -0.537327        2.450276        1.545199
H           1.742035       -1.559351        1.383299
H           0.397378       -3.625600        1.645350
H           1.251723       -4.290588        0.237644
H          -5.768467       -0.190999       -0.171539
H          -4.821299       -0.620155       -1.607718
H          -3.629699        1.579857       -1.375545
H          -4.579724        1.881749        0.095206
H           3.541424       -2.902781        0.288811
H           2.629124       -2.903344       -1.216863
H           2.790202       -0.415224       -1.323490
H           4.342029       -1.213565       -1.449957
H           4.959818       -0.638665        0.812843
H           3.335371       -0.139895        1.225164
H           4.739322        1.822033        0.903982
H           5.232470        1.289604       -0.697191
H           3.505472        3.104686       -0.725788
H           3.022988        1.681222       -1.622427
H           2.103398        2.377657        1.216019
H           1.629467        0.926979        0.348386
H           1.058495        3.762965       -0.661037
H           0.534643        2.276877       -1.425578
H          -2.290323        3.188730        0.275141
```

VOA-5LOH-M06-2X-D3-C924

__Requested operations__

Run with Gaussian 2016+B.01.

`freq=(VCD,ROA) CPHF=RdFreq M062X/6-31+G(2d,p) EmpiricalDispersion=GD3 SCRF=(Solvent=chloroform) test`

__Relevant magnitudes__

Datum	Value
Charge	0
Multiplicity	1
Stoichiometry	C20H30O2
Number of Basis Functions	700
Electronic Energy (Eh)	-930.21378956
Sum of electronic and zero-point Energies (Eh)	-929.747307
Sum of electronic and thermal Energies (Eh)	-929.726417
Sum of electronic and enthalpy Energies (Eh)	-929.725473
Sum of electronic and thermal Free Energies (Eh)	-929.797558
Number of Imaginary Frequencies	0
Mean of alpha and beta Electrons	83

__Molecular Geometry in Cartesian Coordinates__

```xyz
C          -3.183949       0.499622        0.506042
C          -3.455184       2.023171        0.351675
C          -2.163342       2.196478       -0.475557
C          -1.896088       0.655255       -0.339496
C          -0.909106       2.491074        0.360790
C          -0.647099       0.938269        0.499808
C           0.364138       2.622411       -0.485228
C           0.618431       1.065828       -0.350442
C           1.625724       2.884476        0.348456
C           1.871240       1.334951        0.490542
C           2.909920       2.992245       -0.505102
C           3.161862       1.468281       -0.344251
H          -1.779248      -0.013927       -1.194508
H          -2.249911       2.719353       -1.430187
H           0.716609       0.377173       -1.196323
H           0.277138       3.160813       -1.433757
H          -0.554944       0.411710        1.455169
H          -1.019460       3.182363        1.201440
H           1.946491       0.804335        1.442157
H           1.528855       3.569256        1.193363
C          -4.273009      -0.394945       -0.076491
C          -4.095523      -1.909225        0.108932
C          -2.859092      -2.520451       -0.568461
C          -1.586297      -2.444604        0.282739
```

```
C          -0.297807       -2.547365       -0.530338
C           0.951353       -2.303371        0.313229
C           2.216067       -2.243866       -0.531219
C           3.435402       -1.839125        0.251548
O           4.536652       -1.804355       -0.520044
O           3.463631       -1.565169        1.424387
H          -2.973967        0.194606        1.539240
H          -3.503068        2.605435        1.276421
H          -4.360863        2.218355       -0.230783
H           3.687900        3.605705       -0.046997
H           2.746940        3.345223       -1.527207
H           3.180054        0.881286       -1.268412
H           4.065790        1.243237        0.226215
H          -5.227480       -0.103598        0.381905
H          -4.369598       -0.173027       -1.149872
H          -4.067644       -2.141024        1.182712
H          -4.995975       -2.393589       -0.284524
H          -3.056357       -3.570667       -0.812162
H          -2.693095       -2.016671       -1.530233
H          -1.569139       -1.503474        0.841050
H          -1.610671       -3.238753        1.040086
H          -0.237237       -3.528234       -1.020798
H          -0.330203       -1.802908       -1.338680
H           0.845883       -1.356620        0.854975
H           1.053191       -3.083913        1.075586
H           2.432344       -3.204634       -1.012031
H           2.110370       -1.519224       -1.348906
H           5.279178       -1.527043        0.037275
```

VOA-5LOH-M06-2X-D3-C5

__Requested operations__

Run with Gaussian 2016+B.01.

`freq=(VCD,ROA) CPHF=RdFreq M062X/6-31+G(2d,p) EmpiricalDispersion=GD3 SCRF=(Solvent=chloroform) test`

__Relevant magnitudes__

Datum	Value
Charge	0
Multiplicity	1

Stoichiometry	C20H30O2
Number of Basis Functions	700
Electronic Energy (Eh)	-930.2142545389999
Sum of electronic and zero-point Energies (Eh)	-929.747267
Sum of electronic and thermal Energies (Eh)	-929.726603
Sum of electronic and enthalpy Energies (Eh)	-929.725659
Sum of electronic and thermal Free Energies (Eh)	-929.797103
Number of Imaginary Frequencies	0
Mean of alpha and beta Electrons	83

__Molecular Geometry in Cartesian Coordinates__

```xyz
C           1.246742        2.180095       -0.177993
C           0.371200        3.459033       -0.263320
C          -0.801235        2.718618        0.408506
C           0.107223        1.443914        0.572844
C          -1.703017        1.945724       -0.570219
C          -0.822941        0.650088       -0.342633
C          -2.884813        1.265946        0.123977
C          -1.989097       -0.007622        0.406470
C          -3.752686        0.431259       -0.830537
C          -2.882852       -0.842738       -0.513469
C          -4.960570       -0.235072       -0.138403
C          -4.092015       -1.473926        0.210863
H           0.369146        0.988867        1.530898
H          -1.307930        3.221669        1.234817
H          -1.766876       -0.406788        1.400431
H          -3.411105        1.842413        0.890871
H          -0.365348        0.065333       -1.148066
H          -1.892275        2.410737       -1.542577
H          -2.382888       -1.451457       -1.270655
H          -3.908351        0.836514       -1.832634
C           2.539821        2.326068        0.615715
C           3.281665        0.996076        0.784762
C           4.003808        0.546020       -0.488566
C           4.451786       -0.920324       -0.473490
C           3.321145       -1.920298       -0.191191
C           2.077975       -1.669141       -1.043898
C           0.931306       -2.637645       -0.778934
C           0.256102       -2.411532        0.549280
O          -0.842552       -3.175851        0.685783
O           0.617609       -1.649252        1.409276
H           1.452613        1.733999       -1.158541
H           0.772670        4.265791        0.357704
H           0.170895        3.854772       -1.263340
H          -5.772767       -0.471324       -0.828784
```

```
H          -5.374100        0.322302        0.706759
H          -4.448365       -2.397133       -0.249887
H          -3.932941       -1.658007        1.277757
H           3.190469        3.063112        0.123934
H           2.288704        2.739174        1.601845
H           4.012001        1.072274        1.599321
H           2.552843        0.235766        1.094724
H           4.879511        1.186832       -0.647927
H           3.355632        0.709603       -1.357531
H           5.238354       -1.056334        0.278830
H           4.906253       -1.154672       -1.444226
H           3.688719       -2.937019       -0.378066
H           3.041593       -1.878865        0.865713
H           1.709464       -0.653630       -0.863644
H           2.345665       -1.724899       -2.105905
H           1.266502       -3.681890       -0.798738
H           0.153348       -2.557177       -1.546233
H          -1.229183       -2.986405        1.554088
```

VOA-5LOH-M06-2X-D3-C164

__Requested operations__

Run with Gaussian 2016+B.01.

`freq=(VCD,ROA) CPHF=RdFreq M062X/6-31+G(2d,p) EmpiricalDispersion=GD3 SCRF=(Solvent=chloroform) test`

__Relevant magnitudes__

Datum	Value
Charge	0
Multiplicity	1
Stoichiometry	C20H30O2
Number of Basis Functions	700
Electronic Energy (Eh)	-930.213663645
Sum of electronic and zero-point Energies (Eh)	-929.746696
Sum of electronic and thermal Energies (Eh)	-929.725882
Sum of electronic and enthalpy Energies (Eh)	-929.724937
Sum of electronic and thermal Free Energies (Eh)	-929.796902
Number of Imaginary Frequencies	0
Mean of alpha and beta Electrons	83

__Molecular Geometry in Cartesian Coordinates__

```xyz
C          2.433835       -1.824721        0.181923
C          2.108988       -3.142893       -0.566940
C          0.754085       -2.571445       -1.010980
C          1.161530       -1.169832       -0.433486
C         -0.367259       -2.660961        0.043056
C         -0.032306       -1.186774        0.518091
C         -1.710790       -2.183878       -0.506482
C         -1.346299       -0.694664       -0.109962
C         -2.829745       -2.185340        0.546742
C         -2.506419       -0.684437        0.889035
C         -4.203039       -1.759684       -0.008739
C         -3.855979       -0.268815        0.250053
H          1.323326       -0.285395       -1.053933
H          0.444188       -2.741834       -2.044036
H         -1.283389        0.139309       -0.816472
H         -1.981879       -2.512340       -1.514156
H          0.151087       -0.891575        1.557085
H         -0.370052       -3.541425        0.692601
H         -2.294797       -0.317123        1.895676
H         -2.810848       -2.998781        1.274836
C          3.782774       -1.193185       -0.141325
C          4.181189       -0.066457        0.818526
C          3.165161        1.072615        0.931484
C          3.075243        1.950361       -0.317664
C          1.987280        3.023119       -0.225442
C          0.574144        2.447979       -0.297841
C         -0.501870        3.537456       -0.180236
C         -1.860315        2.900705       -0.244842
O         -2.269178        2.452285        0.953478
O         -2.510557        2.742095       -1.248540
H          2.335612       -1.927433        1.270883
H          2.783997       -3.285109       -1.417319
H          2.087378       -4.063908        0.023340
H         -5.035330       -2.106107        0.606833
H         -4.396146       -2.039499       -1.047720
H         -4.550984        0.217249        0.941295
H         -3.754643        0.352008       -0.646091
H          3.772914       -0.838409       -1.181034
H          4.552155       -1.974831       -0.095808
H          5.153187        0.344082        0.516602
H          4.325318       -0.506742        1.813062
H          3.439265        1.712938        1.779285
H          2.181519        0.651632        1.175519
H          4.047941        2.432761       -0.473293
```

```
H          2.893820       1.334035       -1.207675
H          2.104941       3.582112        0.712698
H          2.112842       3.746869       -1.039511
H          0.417677       1.707359        0.495703
H          0.443911       1.924274       -1.253061
H         -0.417473       4.245965       -1.006519
H         -0.392011       4.070230        0.767846
H         -3.106888       1.979755        0.824043
```

VOA-5LOH-M06-2X-D3-C69

__Requested operations__

Run with Gaussian 2016+B.01.

`freq=(VCD,ROA) CPHF=RdFreq M062X/6-31+G(2d,p) EmpiricalDispersion=GD3 SCRF=(Solvent=chloroform) test`

__Relevant magnitudes__

Datum	Value
Charge	0
Multiplicity	1
Stoichiometry	C20H30O2
Number of Basis Functions	700
Electronic Energy (Eh)	-930.2133757200002
Sum of electronic and zero-point Energies (Eh)	-929.746572
Sum of electronic and thermal Energies (Eh)	-929.725782
Sum of electronic and enthalpy Energies (Eh)	-929.724837
Sum of electronic and thermal Free Energies (Eh)	-929.797154
Number of Imaginary Frequencies	0
Mean of alpha and beta Electrons	83

__Molecular Geometry in Cartesian Coordinates__

```xyz
C          1.536995      -2.387578        0.418506
C          0.742546      -3.717973        0.350630
C         -0.396265      -3.005828       -0.401921
C          0.477026      -1.703564       -0.485991
C         -1.402762      -2.254091        0.488198
C         -0.556015      -0.923590        0.327253
C         -2.536923      -1.627867       -0.330977
```

```
C          -1.683006     -0.304398     -0.502902
C          -3.549571     -0.856508      0.521689
C          -2.691597      0.454026      0.368765
C          -4.673453     -0.197461     -0.309888
C          -3.834708      1.103684     -0.435850
H           0.830501     -1.273402     -1.427112
H          -0.820781     -3.513798     -1.270321
H          -1.409688      0.156929     -1.455708
H          -2.936376     -2.226842     -1.155082
H          -0.175329     -0.322342      1.162000
H          -1.669568     -2.715461      1.443763
H          -2.281229      1.014626      1.212104
H          -3.830540     -1.299046      1.479811
C           2.967749     -2.399077     -0.103824
C           3.502508     -0.994117     -0.407883
C           3.299817      0.001906      0.733348
C           3.840398      1.401925      0.435601
C           3.237137      2.056250     -0.814576
C           1.706960      2.066113     -0.864453
C           1.075161      2.763035      0.334000
C          -0.403013      2.992129      0.163229
O          -0.980909      3.417454      1.302084
O          -1.028440      2.843243     -0.854872
H           1.523022     -1.956871      1.425706
H           0.476558     -4.188896      1.301477
H           1.247938     -4.456941     -0.278834
H          -5.603206     -0.059206      0.245409
H          -4.903718     -0.710827     -1.247789
H          -3.558448      1.414100     -1.446587
H          -4.294454      1.954529      0.072684
H           3.612066     -2.896191      0.633564
H           3.014195     -3.003256     -1.019244
H           4.570172     -1.053177     -0.654629
H           3.003346     -0.616889     -1.308437
H           2.228854      0.075443      0.961752
H           3.776757     -0.385019      1.643226
H           3.659215      2.043719      1.306657
H           4.929386      1.354588      0.313246
H           3.599275      3.089911     -0.877705
H           3.609246      1.549195     -1.711502
H           1.379482      2.571792     -1.777428
H           1.314997      1.042653     -0.926820
H           1.221716      2.206555      1.265911
H           1.527704      3.749739      0.499937
H          -1.920199      3.566714      1.114525
```

VOA-5LOH-M06-2X-D3-C244

__Requested operations__

Run with Gaussian 2016+B.01.

`freq=(VCD,ROA) CPHF=RdFreq M062X/6-31+G(2d,p) EmpiricalDispersion=GD3 SCRF=(Solvent=chloroform) test`

__Relevant magnitudes__

Datum	Value
Charge	0
Multiplicity	1
Stoichiometry	C20H30O2
Number of Basis Functions	700
Electronic Energy (Eh)	-930.213361648
Sum of electronic and zero-point Energies (Eh)	-929.746564
Sum of electronic and thermal Energies (Eh)	-929.725819
Sum of electronic and enthalpy Energies (Eh)	-929.724875
Sum of electronic and thermal Free Energies (Eh)	-929.796888
Number of Imaginary Frequencies	0
Mean of alpha and beta Electrons	83

__Molecular Geometry in Cartesian Coordinates__

```xyz
C          1.615904      -2.345439       0.281431
C          0.907199      -3.626116      -0.227448
C         -0.247791      -2.779208      -0.777275
C          0.579470      -1.475979      -0.493068
C         -1.285819      -2.324073       0.271868
C         -0.533029      -0.936161       0.399307
C         -2.478498      -1.627268      -0.375330
C         -1.682724      -0.260076      -0.372630
C         -3.472416      -1.041420       0.643443
C         -2.736522       0.346975       0.554514
C         -4.723908      -0.416747      -0.001406
C         -3.958007       0.917096      -0.208406
H          0.982920      -0.839306      -1.286141
H         -0.656390      -3.043726      -1.754760
H         -1.416455       0.303702      -1.272192
H         -2.908085      -2.095441      -1.266177
H         -0.242694      -0.466763       1.346038
```

```
H          -1.480374       -3.006612        1.104469
H          -2.382765        0.909066        1.421290
H          -3.606299       -1.607324        1.567613
C           3.090768       -2.205259       -0.080473
C           3.841471       -1.183032        0.777159
C           3.285768        0.241386        0.735630
C           3.436333        0.933870       -0.620142
C           3.255790        2.453988       -0.539578
C           1.958848        2.907115        0.133574
C           0.712603        2.390803       -0.579753
C          -0.557956        2.831409        0.094552
O          -1.573902        2.986119       -0.770640
O          -0.689414        3.015964        1.278868
H           1.496201       -2.214478        1.365087
H           0.657431       -4.392496        0.512537
H           1.480547       -4.089379       -1.037376
H          -5.550006       -0.312940        0.705094
H          -5.093719       -0.916805       -0.900902
H          -3.742426        1.175007       -1.249572
H          -4.433841        1.774927        0.273461
H           3.573283       -3.182396        0.050274
H           3.181940       -1.961497       -1.148081
H           4.896412       -1.160872        0.476044
H           3.822004       -1.536110        1.815909
H           3.810459        0.840268        1.491830
H           2.228916        0.230872        1.039186
H           4.436691        0.724568       -1.019935
H           2.730207        0.509365       -1.344252
H           4.097286        2.877336        0.021892
H           3.308338        2.879904       -1.549085
H           1.933343        2.574835        1.175835
H           1.928522        4.001698        0.157355
H           0.687077        2.695282       -1.629942
H           0.690316        1.292517       -0.568110
H          -2.367221        3.208950       -0.259032
```

VOA-5LOH-M06-2X-D3-C128

__Requested operations__

Run with Gaussian 2016+B.01.

`freq=(VCD,ROA) CPHF=RdFreq M062X/6-31+G(2d,p) EmpiricalDispersion=GD3 SCRF=(Solvent=chloroform) test`

__Relevant magnitudes__

Datum	Value
Charge	0
Multiplicity	1
Stoichiometry	C20H30O2
Number of Basis Functions	700
Electronic Energy (Eh)	-930.213293685
Sum of electronic and zero-point Energies (Eh)	-929.746437
Sum of electronic and thermal Energies (Eh)	-929.725678
Sum of electronic and enthalpy Energies (Eh)	-929.724734
Sum of electronic and thermal Free Energies (Eh)	-929.796439
Number of Imaginary Frequencies	0
Mean of alpha and beta Electrons	83

__Molecular Geometry in Cartesian Coordinates__

```xyz
C          -1.296468        2.495827        0.448771
C          -0.343048        3.708568        0.623098
C           0.740576        2.991082       -0.206041
C          -0.244167        1.788647       -0.442575
C           1.658549        2.071225        0.611364
C           0.676822        0.854949        0.351351
C           2.725230        1.393346       -0.256823
C           1.746976        0.172113       -0.504850
C           3.660010        0.478984        0.541047
C           2.679818       -0.730201        0.316131
C           4.718758       -0.230756       -0.331282
C           3.757533       -1.432148       -0.540633
H          -0.571410        1.452353       -1.430426
H           1.217204        3.558973       -1.007609
H           1.439053       -0.199804       -1.487067
H           3.177748        2.002912       -1.044786
H           0.209743        0.249109        1.137292
H           1.971360        2.423511        1.598654
H           2.223335       -1.305405        1.124167
H           3.978759        0.832970        1.523454
C          -2.640324        2.793115       -0.209541
C          -3.317937        1.551191       -0.802851
C          -3.290776        0.329976        0.112566
C          -3.869197       -0.916300       -0.551222
C          -3.735315       -2.185261        0.296388
C          -2.309783       -2.483997        0.773486
C          -1.291074       -2.496960       -0.359737
```

```
C            0.080499       -2.916962        0.092947
O            0.940661       -3.013113       -0.935239
O            0.409891       -3.140757        1.230510
H           -1.462938        1.949984        1.383886
H           -0.058045        3.965204        1.647362
H           -0.726839        4.607145        0.131370
H            5.625410       -0.498249        0.214257
H            5.005012        0.314596       -1.234807
H            3.461382       -1.635417       -1.574580
H            4.136097       -2.355769       -0.094471
H           -3.296771        3.262926        0.534573
H           -2.496779        3.532495       -1.008161
H           -4.353379        1.791664       -1.072456
H           -2.815568        1.282850       -1.741253
H           -2.250249        0.131313        0.395784
H           -3.826454        0.541166        1.049044
H           -4.927309       -0.751304       -0.787033
H           -3.365650       -1.065638       -1.516046
H           -4.383367       -2.104572        1.177050
H           -4.104468       -3.039481       -0.284283
H           -1.996044       -1.752341        1.525536
H           -2.293522       -3.456312        1.275338
H           -1.595557       -3.168277       -1.171356
H           -1.175151       -1.506082       -0.820211
H            1.809902       -3.248201       -0.574951
```

d5LOH-B3LYP

VOA-d5LOH-B3LYP-C139

__Requested operations__

Run with Gaussian 2016+B.01.

`freq=(VCD,ROA) CPHF=Rdfreq B3LYP/6-31+G(2d,p) SCRF=(Solvent=chloroform,Read) test`

__Relevant magnitudes__

Datum	Value
Charge	0
Multiplicity	1
Stoichiometry	C40H60O4
Number of Basis Functions	1400
Electronic Energy (Eh)	-1861.2630803800002
Sum of electronic and zero-point Energies (Eh)	-1860.340007
Sum of electronic and thermal Energies (Eh)	-1860.294972
Sum of electronic and enthalpy Energies (Eh)	-1860.294027
Sum of electronic and thermal Free Energies (Eh)	-1860.432215
Number of Imaginary Frequencies	0
Mean of alpha and beta Electrons	166

__Molecular Geometry in Cartesian Coordinates__

```xyz
C          -2.582341       -3.726107        0.805364
C          -1.263524       -3.415372        1.466149
C          -0.146336       -3.002713        0.507735
C           1.162371       -2.686696        1.241177
C           2.297872       -2.269945        0.299237
C           3.606114       -1.940320        1.027465
C           4.743694       -1.525198        0.086819
C           6.045001       -1.173395        0.818259
C           7.186567       -0.779142       -0.116998
O          -3.523869       -4.085493        1.662324
O          -2.772811       -3.654606       -0.406439
C           7.824037       -1.944061       -0.941816
C           9.204051       -1.671147       -0.295290
C           8.557275       -0.518548        0.572479
C          10.066029       -0.629207       -1.037781
C           9.432260        0.525880       -0.143605
```

C	11.435309	-0.390777	-0.376817
C	10.799787	0.756641	0.526381
C	12.295080	0.663313	-1.101534
C	11.667926	1.802409	-0.199211
C	13.676358	0.917151	-0.442029
C	13.057611	2.035864	0.450791
H	8.551628	-0.501937	1.667119
H	9.734291	-2.531066	0.123248
H	10.816934	0.772634	1.621461
H	11.957409	-1.273098	0.008850
H	8.921989	1.411758	-0.537218
H	10.042562	-0.656951	-2.132487
H	11.152134	2.682807	-0.592713
H	12.261896	0.657961	-2.194598
H	-1.456710	-2.631570	2.210083
H	-0.980053	-4.301691	2.048314
H	0.022175	-3.804239	-0.221439
H	-0.466880	-2.126847	-0.068792
H	0.985381	-1.884946	1.972468
H	1.476874	-3.565421	1.822352
H	2.479181	-3.074738	-0.427522
H	1.979210	-1.396141	-0.286956
H	3.423268	-1.133982	1.752458
H	3.923305	-2.812986	1.616510
H	4.933781	-2.336472	-0.629949
H	4.422690	-0.659761	-0.510774
H	5.856036	-0.345833	1.517748
H	6.361140	-2.028349	1.434682
H	6.863343	0.067242	-0.736869
H	-4.383721	-4.280108	1.181385
H	7.786640	-1.851977	-2.032572
H	7.412875	-2.921630	-0.667967
H	14.428165	1.292062	-1.142292
H	14.102027	0.057559	0.086591
H	13.096189	1.866012	1.532076
H	13.470733	3.027898	0.247603
C	-6.020850	-4.520359	-0.768683
C	-7.336703	-4.875463	-1.416210
C	-8.589625	-4.507847	-0.602327
C	-9.046511	-3.046736	-0.743605
C	-8.078871	-1.991569	-0.192709
C	-8.628888	-0.563171	-0.290677
C	-7.667020	0.499755	0.253624
C	-8.217883	1.928354	0.165695
C	-7.253744	2.995475	0.681624
O	-5.084623	-4.147718	-1.624882
O	-5.822003	-4.612382	0.440915

```
C          -7.756694        4.466931        0.547414
C          -6.539442        4.824053       -0.340616
C          -6.042521        3.326764       -0.241881
C          -5.291667        5.290596        0.437616
C          -4.781409        3.784297        0.513081
C          -4.104499        5.647906       -0.474365
C          -3.599616        4.138844       -0.408806
C          -2.847348        6.096275        0.296497
C          -2.343017        4.597245        0.355793
C          -1.641087        6.454172       -0.611380
C          -1.146733        4.976293       -0.557166
H          -5.896451        2.671680       -1.106314
H          -6.746896        5.341019       -1.281626
H          -3.441418        3.493528       -1.279561
H          -4.336948        6.190064       -1.397323
H          -4.539980        3.252488        1.439809
H          -5.462354        5.937504        1.304875
H          -2.108855        4.064848        1.281948
H          -3.008276        6.725086        1.176630
H          -7.366108       -4.441503       -2.419907
H          -7.297067       -5.965144       -1.550283
H          -9.405443       -5.157129       -0.938870
H          -8.413321       -4.748814        0.452195
H          -9.245488       -2.835542       -1.804346
H         -10.010961       -2.942735       -0.228383
H          -7.848424       -2.222155        0.856434
H          -7.123688       -2.036045       -0.733470
H          -8.864009       -0.336177       -1.340587
H          -9.581901       -0.504303        0.254688
H          -7.429154        0.270078        1.302209
H          -6.716250        0.439414       -0.294333
H          -8.483068        2.155651       -0.877731
H          -9.154741        1.993117        0.738702
H          -6.942220        2.732635        1.700829
H          -4.219674       -3.971462       -1.146018
H          -7.847709        5.038340        1.477593
H          -8.709578        4.531850        0.011145
H          -0.944223        7.158287       -0.147966
H          -1.905385        6.838863       -1.602175
H          -1.109666        4.445079       -1.514209
H          -0.174856        4.864202       -0.068260
```

VOA-d5LOH-B3LYP-C58

__Requested operations__

Run with Gaussian 2016+B.01.

`freq=(VCD,ROA) CPHF=Rdfreq B3LYP/6-31+G(2d,p) SCRF=(Solvent=chloroform,Read) test`

__Relevant magnitudes__

Datum	Value
Charge	0
Multiplicity	1
Stoichiometry	C40H60O4
Number of Basis Functions	1400
Electronic Energy (Eh)	-1861.26313223
Sum of electronic and zero-point Energies (Eh)	-1860.339626
Sum of electronic and thermal Energies (Eh)	-1860.294811
Sum of electronic and enthalpy Energies (Eh)	-1860.293867
Sum of electronic and thermal Free Energies (Eh)	-1860.431371
Number of Imaginary Frequencies	0
Mean of alpha and beta Electrons	166

__Molecular Geometry in Cartesian Coordinates__

```xyz
C          -1.182882       -0.528933        1.451166
C          -0.702310       -1.953342        1.331262
C           0.558527       -2.125571        0.463289
C           1.800789       -1.431681        1.034104
C           3.055547       -1.647097        0.179855
C           4.304873       -0.962926        0.747079
C           5.561278       -1.176462       -0.105572
C           6.807738       -0.483511        0.458587
C           8.067245       -0.711538       -0.375204
O          -1.376032        0.075943        0.292612
O          -1.392663        0.016999        2.533748
C           8.680066       -2.147826       -0.301951
C          10.004160       -1.574266        0.259283
C           9.378181       -0.122824        0.221917
C          11.030650       -1.160332       -0.815193
C          10.413510        0.307359       -0.832974
C          12.338421       -0.604229       -0.224127
C          11.718368        0.862641       -0.232148
C          13.363074       -0.171259       -1.290877
C          12.749706        1.287801       -1.295020
C          14.683320        0.397918       -0.707353
C          14.077951        1.834920       -0.708107
```

H	9.262148	0.519920	1.100515
H	10.406499	-2.046363	1.159861
H	11.621874	1.505931	0.649085
H	12.733657	-1.111221	0.662772
H	10.029017	0.812772	-1.725614
H	11.120582	-1.811794	-1.691103
H	12.360271	1.787282	-2.186560
H	13.446716	-0.804591	-2.178473
H	-0.537475	-2.330081	2.343537
H	-1.526511	-2.528051	0.890565
H	0.752105	-3.201121	0.370122
H	0.355011	-1.755107	-0.547725
H	1.612067	-0.352964	1.128633
H	1.985547	-1.800038	2.053128
H	3.245465	-2.725664	0.081443
H	2.868535	-1.275885	-0.837874
H	4.113348	0.115205	0.846647
H	4.490467	-1.334361	1.765202
H	5.753431	-2.254419	-0.201744
H	5.375355	-0.807896	-1.124738
H	6.620402	0.597825	0.531980
H	6.986572	-0.832984	1.486577
H	7.882989	-0.367605	-1.401288
H	-1.707174	1.013859	0.433533
H	8.765601	-2.694178	-1.247378
H	8.156334	-2.788472	0.415652
H	15.537171	0.290158	-1.382228
H	14.968895	-0.005084	0.270172
H	13.986011	2.319855	0.269589
H	14.599066	2.522041	-1.380715
C	-2.493412	3.095947	1.688668
C	-2.971389	4.521798	1.819332
C	-3.754973	5.044968	0.610393
C	-5.137262	4.399885	0.448285
C	-5.914618	4.948494	-0.755608
C	-7.332953	4.377585	-0.916714
C	-7.388527	2.884992	-1.269823
C	-8.815245	2.366838	-1.490999
C	-8.885561	0.875379	-1.815096
O	-2.318506	2.486009	2.848051
O	-2.257387	2.559042	0.608107
C	-10.325739	0.294688	-1.973287
C	-10.073223	-0.702829	-0.815955
C	-8.626693	-0.095537	-0.623571
C	-9.452274	-2.046849	-1.249885
C	-7.996755	-1.439374	-1.032004
C	-9.223146	-3.017790	-0.077617

```
C          -7.771005       -2.403675        0.147326
C          -8.585719       -4.355929       -0.500145
C          -7.142894       -3.746574       -0.272178
C          -8.342463       -5.340900        0.673925
C          -6.922049       -4.738649        0.900421
H          -8.272472        0.359189        0.306727
H         -10.829380       -0.748659       -0.027319
H          -7.416497       -1.962896        1.085263
H         -10.007650       -3.063915        0.685429
H          -7.213167       -1.405687       -1.796617
H          -9.815305       -2.480171       -2.188070
H          -6.365079       -3.707316       -1.039944
H          -8.927606       -4.787222       -1.445140
H          -3.550872        4.607091        2.743958
H          -2.064471        5.123067        1.972324
H          -3.870989        6.129444        0.726675
H          -3.163014        4.888025       -0.298420
H          -5.018973        3.314018        0.351810
H          -5.723814        4.568845        1.363267
H          -5.983379        6.041056       -0.660938
H          -5.341066        4.757622       -1.674108
H          -7.900654        4.553365        0.008742
H          -7.851452        4.943824       -1.702649
H          -6.796287        2.708469       -2.179583
H          -6.911674        2.295305       -0.476757
H          -9.422013        2.570523       -0.595883
H          -9.285185        2.929483       -2.311296
H          -8.234310        0.665663       -2.673447
H          -1.962136        1.556779        2.707917
H         -10.566408       -0.156033       -2.942276
H         -11.099052        1.033928       -1.738145
H          -8.320876       -6.389135        0.362704
H          -9.044777       -5.247928        1.509157
H          -6.746547       -4.270692        1.874967
H          -6.116041       -5.454829        0.717038
```

d5LOH-B3LYP-D3B(J)

VOA-d5LOH-B3LYP-D3BJ-C336

__Requested operations__

Run with Gaussian 2016+B.01.

`freq=(VCD,ROA) CPHF=Rdfreq B3LYP/6-31+G(2d,p) SCRF=(Solvent=chloroform,Read) EmpiricalDispersion=GD3BJ test`

__Relevant magnitudes__

Datum	Value
Charge	0
Multiplicity	1
Stoichiometry	C40H60O4
Number of Basis Functions	1400
Electronic Energy (Eh)	-1861.47528897
Sum of electronic and zero-point Energies (Eh)	-1860.547151
Sum of electronic and thermal Energies (Eh)	-1860.503637
Sum of electronic and enthalpy Energies (Eh)	-1860.502693
Sum of electronic and thermal Free Energies (Eh)	-1860.628595
Number of Imaginary Frequencies	0
Mean of alpha and beta Electrons	166

__Molecular Geometry in Cartesian Coordinates__

```xyz
C          -2.439100       3.680029      -0.608806
C          -3.897535       3.550092      -0.963051
C          -4.485791       2.189447      -0.550179
C          -4.333530       1.911394       0.946382
C          -4.899571       0.553390       1.365601
C          -4.664382       0.208404       2.844778
C          -3.194083       0.246586       3.293304
C          -2.265387      -0.637132       2.458423
C          -0.803468      -0.553596       2.875360
O          -1.690653       2.695241      -1.062834
O          -1.989781       4.616793       0.051275
C          -0.089067       0.779138       2.484203
C           0.894290       0.004622       1.578400
C           0.165639      -1.343571       1.956951
C           2.165684      -0.481084       2.297359
```

C	1.434647	-1.845899	2.661705
C	3.121167	-1.242833	1.368157
C	2.386747	-2.606045	1.724639
C	4.393058	-1.746949	2.068492
C	3.665460	-3.107374	2.416741
C	5.355376	-2.513897	1.127114
C	4.636041	-3.854112	1.466151
H	-0.300231	-2.011118	1.227845
H	1.017378	0.372759	0.560881
H	1.926383	-3.274835	0.990717
H	3.239997	-0.853920	0.356119
H	1.317363	-2.241249	3.675804
H	2.618350	0.199860	3.025155
H	3.555951	-3.504203	3.429180
H	4.843297	-1.082826	2.810487
H	-4.428732	4.373528	-0.480893
H	-3.985223	3.679227	-2.048286
H	-5.545032	2.174015	-0.831528
H	-3.992536	1.395098	-1.121040
H	-3.268873	1.955935	1.204138
H	-4.819731	2.709634	1.524195
H	-5.978256	0.532990	1.163023
H	-4.461827	-0.226303	0.730256
H	-5.241794	0.900933	3.471560
H	-5.071101	-0.793285	3.037349
H	-2.825639	1.279842	3.269835
H	-3.137613	-0.066538	4.344019
H	-2.602476	-1.682345	2.512436
H	-2.331403	-0.352305	1.401727
H	-0.703328	-0.818714	3.935207
H	-0.739734	2.794690	-0.749075
H	0.371787	1.346632	3.299050
H	-0.741107	1.455125	1.922801
H	6.397642	-2.489621	1.455727
H	5.318653	-2.199089	0.079221
H	4.152816	-4.365602	0.627449
H	5.281548	-4.571785	1.979117
C	1.268749	3.845514	0.362609
C	2.719575	3.956608	0.757449
C	3.590442	2.755368	0.388595
C	3.791857	2.559877	-1.117221
C	4.714063	1.376768	-1.434008
C	4.661961	0.898160	-2.893900
C	3.260021	0.507286	-3.396078
C	2.518808	-0.471214	-2.482108
C	1.101326	-0.793094	-2.939134
O	0.549451	4.894535	0.720123

```
O           0.792293        2.879182       -0.226838
C           0.105001        0.400323       -2.794088
C          -0.709486       -0.385558       -1.742318
C           0.283862       -1.602560       -1.897704
C          -1.847380       -1.229848       -2.339490
C          -0.848578       -2.458838       -2.486447
C          -2.641506       -1.999036       -1.276037
C          -1.639393       -3.225871       -1.413270
C          -3.774397       -2.862983       -1.852942
C          -2.780313       -4.086056       -1.982044
C          -4.572390       -3.637748       -0.774282
C          -3.590212       -4.841395       -0.897172
H           0.854242       -2.034343       -1.071376
H          -0.918054        0.116508       -0.797904
H          -1.062090       -3.654119       -0.587906
H          -2.853004       -1.472611       -0.343493
H          -0.640933       -2.988756       -3.421609
H          -2.417513       -0.783809       -3.160442
H          -2.579359       -4.618940       -2.914857
H          -4.340898       -2.440342       -2.686527
H           3.103060        4.879916        0.304958
H           2.733335        4.135141        1.839182
H           4.564314        2.883860        0.876608
H           3.147891        1.847354        0.812917
H           2.813398        2.404729       -1.577201
H           4.202375        3.477935       -1.560468
H           5.748521        1.642325       -1.180729
H           4.456125        0.540152       -0.774485
H           5.065934        1.680102       -3.550730
H           5.332572        0.034578       -2.996362
H           2.649195        1.409619       -3.521451
H           3.352950        0.068275       -4.398063
H           3.095592       -1.402231       -2.384754
H           2.450965       -0.049572       -1.472984
H           1.119608       -1.251128       -3.935825
H          -0.412183        4.771617        0.450407
H          -0.453045        0.683367       -3.691873
H           0.581101        1.295915       -2.387649
H          -5.593228       -3.882412       -1.079204
H          -4.614629       -3.148191        0.204074
H          -3.023567       -5.093253        0.005086
H          -4.068904       -5.752102       -1.266437
```

VOA-d5LOH-B3LYP-D3BJ-C310

__Requested operations__

Run with Gaussian 2016+B.01.

`freq=(VCD,ROA) CPHF=Rdfreq B3LYP/6-31+G(2d,p) SCRF=(Solvent=chloroform,Read) EmpiricalDispersion=GD3BJ test`

__Relevant magnitudes__

Datum	Value
Charge	0
Multiplicity	1
Stoichiometry	C40H60O4
Number of Basis Functions	1400
Electronic Energy (Eh)	-1861.47503397
Sum of electronic and zero-point Energies (Eh)	-1860.545992
Sum of electronic and thermal Energies (Eh)	-1860.502673
Sum of electronic and enthalpy Energies (Eh)	-1860.501729
Sum of electronic and thermal Free Energies (Eh)	-1860.625652
Number of Imaginary Frequencies	0
Mean of alpha and beta Electrons	166

__Molecular Geometry in Cartesian Coordinates__

```xyz
C          -3.434046        2.242295       -1.760850
C          -4.835646        1.845144       -2.138987
C          -5.327738        0.640457       -1.310659
C          -5.238527        0.878591        0.198088
C          -5.707253       -0.317896        1.028774
C          -5.598196       -0.102462        2.547537
C          -4.206634        0.318901        3.046504
C          -3.079913       -0.650652        2.679651
C          -1.696878       -0.136444        3.064225
O          -2.520020        1.335262       -2.047183
O          -3.170767        3.308257       -1.204810
C          -1.189240        1.041587        2.173642
C          -0.049794        0.153392        1.620495
C          -0.533153       -1.023846        2.554768
C           1.262348        0.248767        2.412573
C           0.768030       -0.919596        3.370116
C           2.355223       -0.674876        1.849119
C           1.883409       -1.828113        2.834992
C           3.690014       -0.577753        2.599595
C           3.210010       -1.698341        3.608135
```

C	4.778424	-1.546614	2.066361
C	4.330152	-2.632696	3.089195
H	-0.808263	-2.017729	2.192532
H	0.037197	0.079925	0.538130
H	1.594383	-2.835482	2.519083
H	2.397809	-0.767945	0.763661
H	0.668845	-0.838263	4.457213
H	1.575832	1.241398	2.750686
H	3.134727	-1.559671	4.689540
H	4.018831	0.420007	2.900923
H	-5.478899	2.712667	-1.978294
H	-4.848397	1.590947	-3.203812
H	-6.363917	0.431075	-1.599656
H	-4.739684	-0.243284	-1.580267
H	-4.199410	1.112170	0.457229
H	-5.822905	1.769380	0.466938
H	-6.751572	-0.545676	0.778264
H	-5.128643	-1.202506	0.737842
H	-6.323810	0.663335	2.852445
H	-5.898024	-1.029476	3.054142
H	-3.965237	1.315506	2.656632
H	-4.241261	0.427504	4.138375
H	-3.257070	-1.623052	3.160934
H	-3.083187	-0.842724	1.599802
H	-1.658911	0.062254	4.142430
H	-1.610498	1.637469	-1.736269
H	-0.859476	1.942682	2.699645
H	-1.915676	1.340477	1.412639
H	5.799851	-1.191499	2.224842
H	4.670272	-1.815488	1.011260
H	3.984209	-3.581568	2.666635
H	5.092439	-2.849767	3.842126
C	0.070010	2.989239	-0.421973
C	1.424144	3.263299	0.166243
C	2.335944	3.913376	-0.894429
C	3.726593	4.284467	-0.364621
C	4.641668	3.114165	0.031103
C	4.917608	2.110074	-1.108452
C	4.042811	0.855352	-1.032984
C	4.144995	-0.061066	-2.252019
C	3.291177	-1.318621	-2.120311
O	-0.860649	3.857555	-0.077979
O	-0.136180	2.051912	-1.192799
C	3.172423	-2.173188	-3.418311
C	1.644070	-1.925699	-3.464330
C	1.757209	-1.076671	-2.137457
C	0.813581	-2.986783	-2.723150

```
C           0.944865       -2.143069       -1.381924
C          -0.697984       -2.701771       -2.751903
C          -0.564805       -1.874210       -1.401264
C          -1.536570       -3.758873       -2.015594
C          -1.381656       -2.950151       -0.666015
C          -3.057483       -3.453775       -2.004217
C          -2.903423       -2.673224       -0.664896
H           1.416007       -0.044246       -2.039977
H           1.230720       -1.543473       -4.401020
H          -0.888452       -0.839324       -1.276477
H          -1.107696       -2.305357       -3.686280
H           1.367584       -2.520890       -0.446938
H           1.122463       -4.030834       -2.837755
H          -0.944882       -3.358495        0.247769
H          -1.240936       -4.803476       -2.142505
H           1.319832        3.923099        1.030693
H           1.842604        2.311955        0.500395
H           2.420849        3.238337       -1.752674
H           1.847875        4.823556       -1.261825
H           4.234199        4.867418       -1.144119
H           3.610833        4.957429        0.495233
H           5.585912        3.544766        0.383342
H           4.220484        2.578180        0.892117
H           4.771153        2.606488       -2.077873
H           5.969899        1.801379       -1.086558
H           4.316697        0.296334       -0.130678
H           2.994734        1.141451       -0.900736
H           3.833250        0.493779       -3.149082
H           5.192188       -0.352147       -2.418130
H           3.621124       -1.887934       -1.242783
H          -1.731317        3.641875       -0.536031
H           3.468333       -3.223770       -3.332668
H           3.711781       -1.731474       -4.262177
H          -3.681433       -4.346782       -1.914674
H          -3.413725       -2.869442       -2.858673
H          -3.169830       -1.613665       -0.694537
H          -3.433504       -3.137571        0.170521
```

VOA-d5LOH-B3LYP-D3BJ-C178

__Requested operations__

Run with Gaussian 2016+B.01.

`freq=(VCD,ROA) CPHF=Rdfreq B3LYP/6-31+G(2d,p) SCRF=(Solvent=chloroform,Read) EmpiricalDispersion=GD3BJ test`

__Relevant magnitudes__

Datum	Value
Charge	0
Multiplicity	1
Stoichiometry	C40H60O4
Number of Basis Functions	1400
Electronic Energy (Eh)	-1861.47383183
Sum of electronic and zero-point Energies (Eh)	-1860.545718
Sum of electronic and thermal Energies (Eh)	-1860.502198
Sum of electronic and enthalpy Energies (Eh)	-1860.501253
Sum of electronic and thermal Free Energies (Eh)	-1860.627909
Number of Imaginary Frequencies	0
Mean of alpha and beta Electrons	166

__Molecular Geometry in Cartesian Coordinates__

```xyz
C          3.185198      -2.821794      -0.879229
C          4.555452      -2.287991      -1.200613
C          5.519907      -2.475553      -0.012633
C          5.080761      -1.760933       1.271298
C          5.049673      -0.232109       1.164475
C          4.748271       0.467119       2.498590
C          3.335142       0.231971       3.053157
C          2.227794       0.875329       2.214854
C          0.830480       0.614425       2.762820
O          2.232233      -1.912845      -0.873587
O          2.985065      -4.009502      -0.623727
C          0.287103      -0.825192       2.498822
C         -0.859376      -0.251161       1.633892
C         -0.318417       1.206552       1.907290
C         -2.137022       0.070795       2.427646
C         -1.591523       1.538991       2.703124
C         -3.258589       0.652385       1.554774
C         -2.715077       2.120717       1.831460
C         -4.539403       0.981807       2.337790
C         -4.000442       2.442773       2.612584
C         -5.668502       1.574954       1.457748
C         -5.138074       3.014969       1.728843
H         -0.025227       1.907148       1.122670
H         -0.988312      -0.683737       0.642545
```

H	-2.418898	2.832929	1.055330
H	-3.388167	0.217212	0.562464
H	-1.455790	1.980040	3.695943
H	-2.433759	-0.642145	3.203630
H	-3.877307	2.875421	3.608667
H	-4.833814	0.277836	3.120235
H	4.930064	-2.850341	-2.061979
H	4.473797	-1.238465	-1.487381
H	5.622767	-3.547887	0.183155
H	6.506751	-2.110278	-0.319967
H	4.095832	-2.136747	1.576083
H	5.771000	-2.043881	2.076618
H	6.026159	0.112898	0.798894
H	4.316423	0.078725	0.411493
H	5.485440	0.134572	3.241717
H	4.900538	1.547941	2.376683
H	3.142784	-0.844074	3.150565
H	3.283321	0.639701	4.071270
H	2.399734	1.959991	2.162116
H	2.272916	0.507287	1.182127
H	0.786340	0.924315	3.814549
H	1.345238	-2.328947	-0.639257
H	-0.034332	-1.394537	3.376938
H	0.988589	-1.440393	1.928226
H	-6.670603	1.402229	1.858840
H	-5.655374	1.245948	0.413621
H	-4.799502	3.575922	0.851743
H	-5.846596	3.637641	2.281291
C	-0.292255	-4.115077	0.082178
C	-1.631820	-4.643322	0.528350
C	-2.794626	-3.664221	0.366611
C	-3.132962	-3.346745	-1.092507
C	-4.330724	-2.402045	-1.229385
C	-4.537857	-1.844082	-2.646866
C	-3.323639	-1.100913	-3.231551
C	-2.778506	0.004576	-2.325486
C	-1.514260	0.673266	-2.850510
O	0.661212	-5.028898	0.086315
O	-0.096095	-2.945568	-0.237755
C	-0.245270	-0.234633	-2.781287
C	0.392058	0.710032	-1.736444
C	-0.872996	1.648366	-1.829716
C	1.266835	1.816469	-2.348544
C	-0.008045	2.762238	-2.440828
C	1.884047	2.745516	-1.293792
C	0.606037	3.689711	-1.378894
C	2.747492	3.868737	-1.891743

```
C          1.479020       4.809457      -1.969260
C          3.367112       4.809003      -0.827353
C          2.114937       5.733793      -0.899648
H         -1.495651       1.919914      -0.974082
H          0.749001       0.258677      -0.812317
H         -0.030782       3.955201      -0.529165
H          2.252412       2.276819      -0.378504
H         -0.373393       3.233831      -3.358773
H          1.900883       1.536010      -3.195600
H          1.120180       5.283009      -2.886626
H          3.371433       3.606756      -2.750048
H         -1.814741      -5.577162      -0.015960
H         -1.508165      -4.935007       1.579054
H         -3.671752      -4.093871       0.865346
H         -2.558720      -2.732514       0.893457
H         -2.251929      -2.899445      -1.560086
H         -3.335633      -4.279228      -1.638049
H         -5.243495      -2.923892      -0.913887
H         -4.208201      -1.569645      -0.526103
H         -4.809236      -2.664552      -3.324537
H         -5.399300      -1.163215      -2.628171
H         -2.521017      -1.817204      -3.445905
H         -3.605332      -0.668986      -4.200751
H         -3.554875       0.765069      -2.158518
H         -2.544862      -0.410727      -1.338529
H         -1.701860       1.121246      -3.834228
H          1.538706      -4.622670      -0.193826
H          0.324691      -0.344738      -3.709119
H         -0.459584      -1.232202      -2.388402
H          4.284682       5.300452      -1.161435
H          3.562962       4.338403       0.141595
H          1.535672       5.831621       0.024216
H          2.340967       6.737153      -1.269960
```

VOA-d5LOH-B3LYP-D3BJ-C177

__Requested operations__

Run with Gaussian 2016+B.01.

`freq=(VCD,ROA) CPHF=Rdfreq B3LYP/6-31+G(2d,p) SCRF=(Solvent=chloroform,Read) EmpiricalDispersion=GD3BJ test`

__Relevant magnitudes__

Datum	Value
Charge	0
Multiplicity	1
Stoichiometry	C40H60O4
Number of Basis Functions	1400
Electronic Energy (Eh)	-1861.4740215299998
Sum of electronic and zero-point Energies (Eh)	-1860.545579
Sum of electronic and thermal Energies (Eh)	-1860.502155
Sum of electronic and enthalpy Energies (Eh)	-1860.501211
Sum of electronic and thermal Free Energies (Eh)	-1860.62477
Number of Imaginary Frequencies	0
Mean of alpha and beta Electrons	166

__Molecular Geometry in Cartesian Coordinates__

```xyz
C          -4.137683        1.608868       -1.249935
C          -5.319334        0.934466       -1.898134
C          -6.462292        0.581112       -0.933651
C          -6.201686       -0.656411       -0.063833
C          -5.044484       -0.513005        0.927826
C          -4.920154       -1.694398        1.896106
C          -3.727742       -1.550584        2.853388
C          -2.374171       -1.817417        2.182060
C          -1.195645       -1.177509        2.907138
O          -2.969763        1.194467       -1.698667
O          -4.257819        2.490953       -0.400411
C          -1.147291        0.376384        2.754101
C           0.161962        0.268642        1.936693
C           0.146145       -1.296087        2.140304
C           1.435309        0.375618        2.790189
C           1.413402       -1.199192        3.007663
C           2.720395        0.244319        1.961536
C           2.703055       -1.329562        2.180291
C           4.007757        0.345535        2.795109
C           3.992945       -1.220912        3.010736
C           5.305115        0.208090        1.958743
C           5.286598       -1.336150        2.163369
H           0.169764       -2.016534        1.320647
H           0.173500        0.753496        0.961309
H           2.696601       -2.066534        1.373202
H           2.725021        0.733878        0.986550
H           1.393844       -1.697052        3.982312
H           1.436090        1.119709        3.593097
H           3.993847       -1.706044        3.990011
```

H	4.012317	1.077828	3.606391
H	-5.685158	1.648450	-2.647253
H	-4.969121	0.053577	-2.441813
H	-6.671558	1.448101	-0.297182
H	-7.360976	0.406156	-1.534614
H	-7.120154	-0.881890	0.493551
H	-6.018587	-1.524061	-0.713573
H	-4.100976	-0.394649	0.382652
H	-5.176714	0.409407	1.508218
H	-5.850703	-1.775839	2.472456
H	-4.825636	-2.630940	1.327826
H	-3.736381	-0.533354	3.268469
H	-3.845439	-2.229014	3.707191
H	-2.212797	-2.900176	2.093873
H	-2.388551	-1.433207	1.156072
H	-1.149444	-1.536278	3.942929
H	-2.218548	1.675843	-1.232667
H	-1.061903	0.952820	3.680583
H	-1.991333	0.772927	2.181442
H	6.170711	0.692471	2.417876
H	5.226669	0.556766	0.924584
H	5.186887	-1.937392	1.254658
H	6.146073	-1.707334	2.727414
C	-1.084084	3.352061	0.272984
C	0.093231	4.082305	0.868237
C	1.453482	3.679296	0.297823
C	1.636442	4.045842	-1.178945
C	2.991773	3.628547	-1.767881
C	3.324715	2.143670	-1.553683
C	4.255792	1.566049	-2.624057
C	4.788945	0.154942	-2.314480
C	3.751631	-0.803269	-1.738207
O	-2.251456	3.823340	0.671157
O	-0.974873	2.400278	-0.496285
C	4.097299	-2.320752	-1.779726
C	2.740099	-2.621751	-2.451687
C	2.458301	-1.073924	-2.564806
C	1.553453	-2.753767	-1.472460
C	1.226058	-1.208804	-1.663367
C	0.233472	-3.119775	-2.163231
C	-0.076457	-1.576919	-2.394017
C	-0.954214	-3.203012	-1.187372
C	-1.269938	-1.672368	-1.430881
C	-2.297589	-3.575551	-1.862219
C	-2.609962	-2.067682	-2.094098
H	2.352838	-0.538256	-3.511652
H	2.740078	-3.310851	-3.299928

```
H          -0.252461     -1.110067     -3.367838
H           0.277689     -3.875252     -2.954201
H           1.134537     -0.490801     -0.845330
H           1.758801     -3.232305     -0.510262
H          -1.311749     -0.919935     -0.643451
H          -0.755389     -3.637780     -0.205149
H          -0.094168      5.155840      0.749032
H           0.052890      3.893769      1.948660
H           2.232282      4.164913      0.898648
H           1.583363      2.602866      0.432953
H           0.843999      3.562402     -1.760728
H           1.502709      5.129056     -1.302159
H           2.976666      3.844807     -2.844243
H           3.795124      4.246342     -1.344273
H           3.771900      2.008373     -0.561700
H           2.393774      1.564086     -1.547618
H           3.709871      1.550012     -3.576559
H           5.108119      2.241256     -2.776673
H           5.214197     -0.271883     -3.232686
H           5.619189      0.225331     -1.597234
H           3.493395     -0.478311     -0.727526
H          -3.004064      3.295571      0.259882
H           4.283625     -2.814358     -0.820306
H           4.941907     -2.526880     -2.445468
H          -3.004248     -4.061949     -1.184395
H          -2.203701     -4.192856     -2.761483
H          -2.724887     -1.746126     -3.133696
H          -3.480253     -1.722121     -1.532922
```

d5LOH-B3PW91

VOA-d5LOH-B3PW91-C58

__Requested operations__

Run with Gaussian 2016+B.01.

`freq=(VCD,ROA) CPHF=Rdfreq B3PW91/6-31+G(2d,p) SCRF=(Solvent=chloroform,Read) guess=read test`

__Relevant magnitudes__

Datum	Value
Charge	0
Multiplicity	1
Stoichiometry	C40H60O4
Number of Basis Functions	1400
Electronic Energy (Eh)	-1860.61513911
Sum of electronic and zero-point Energies (Eh)	-1859.689152
Sum of electronic and thermal Energies (Eh)	-1859.644355
Sum of electronic and enthalpy Energies (Eh)	-1859.643411
Sum of electronic and thermal Free Energies (Eh)	-1859.78121
Number of Imaginary Frequencies	0
Mean of alpha and beta Electrons	166

__Molecular Geometry in Cartesian Coordinates__

```xyz
C          -1.165913       -0.502260        1.506229
C          -0.675897       -1.920055        1.397220
C           0.570875       -2.095314        0.521161
C           1.813323       -1.399707        1.075602
C           3.056486       -1.617944        0.214377
C           4.306053       -0.933727        0.766975
C           5.551787       -1.151021       -0.091179
C           6.798276       -0.460101        0.460825
C           8.049458       -0.698862       -0.373864
O          -1.335981        0.102370        0.351752
O          -1.406008        0.034381        2.586754
C           8.649997       -2.133014       -0.294713
C           9.976190       -1.566997        0.253464
C           9.361315       -0.117894        0.215166
C          10.992301       -1.164481       -0.828242
```

C	10.388562	0.301136	-0.844394
C	12.303150	-0.619105	-0.247189
C	11.695987	0.845462	-0.251724
C	13.318168	-0.196233	-1.320041
C	12.718202	1.261355	-1.319695
C	14.641452	0.360665	-0.746703
C	14.048914	1.795988	-0.741701
H	9.252495	0.528533	1.092496
H	10.383995	-2.038414	1.152431
H	11.608875	1.490748	0.629490
H	12.701851	-1.129391	0.636742
H	10.002318	0.809474	-1.735118
H	11.071510	-1.819532	-1.702968
H	12.325907	1.764668	-2.208288
H	13.390180	-0.831301	-2.207881
H	-0.501433	-2.285672	2.412271
H	-1.503718	-2.502963	0.973382
H	0.765261	-3.171097	0.430011
H	0.357753	-1.730558	-0.490235
H	1.624439	-0.320647	1.166995
H	2.007317	-1.762032	2.095180
H	3.245629	-2.696915	0.117895
H	2.861088	-1.251681	-0.803658
H	4.115769	0.144940	0.864023
H	4.499235	-1.299742	1.785748
H	5.742323	-2.229642	-0.185493
H	5.359506	-0.786850	-1.110815
H	6.615505	0.622444	0.528385
H	6.980994	-0.802751	1.490638
H	7.864894	-0.358134	-1.401376
H	-1.682911	1.039408	0.491400
H	8.725340	-2.689627	-1.235153
H	8.125582	-2.763143	0.432270
H	15.489882	0.247942	-1.427904
H	14.932064	-0.046702	0.227654
H	13.966044	2.281319	0.236737
H	14.571289	2.480533	-1.416273
C	-2.498245	3.073829	1.717708
C	-2.981634	4.493893	1.838287
C	-3.742130	5.011516	0.619468
C	-5.117940	4.369558	0.439765
C	-5.873581	4.914506	-0.772850
C	-7.287721	4.352400	-0.949330
C	-7.347187	2.863045	-1.292470
C	-8.770053	2.355219	-1.525303
C	-8.847998	0.867128	-1.840673
O	-2.341312	2.465656	2.872830

```
O          -2.242199       2.542938       0.639111
C         -10.285888       0.299469      -2.005677
C         -10.048902      -0.693049      -0.848520
C          -8.606786      -0.095341      -0.645606
C          -9.432315      -2.035351      -1.276549
C          -7.982290      -1.438510      -1.044630
C          -9.222750      -3.002075      -0.103798
C          -7.776946      -2.397389       0.136529
C          -8.589053      -4.338313      -0.519786
C          -7.152147      -3.738506      -0.274866
C          -8.366863      -5.319778       0.653849
C          -6.953059      -4.725820       0.898317
H          -8.258415       0.361940       0.286238
H         -10.812306      -0.735948      -0.066163
H          -7.430264      -1.956434       1.077739
H         -10.017267      -3.044978       0.649584
H          -7.188855      -1.410589      -1.799877
H          -9.789186      -2.467432      -2.218106
H          -6.363752      -3.703222      -1.032514
H          -8.922532      -4.767407      -1.469219
H          -3.575769       4.578934       2.753821
H          -2.078188       5.096328       2.007199
H          -3.858001       6.097047       0.727442
H          -3.135851       4.849504      -0.279339
H          -4.999390       3.282900       0.348362
H          -5.717800       4.540628       1.345715
H          -5.937323       6.008055      -0.684671
H          -5.288463       4.717345      -1.682781
H          -7.867740       4.537668      -0.033300
H          -7.793059       4.916594      -1.745415
H          -6.745096       2.676348      -2.193736
H          -6.882991       2.274904      -0.490325
H          -9.385354       2.568057      -0.637954
H          -9.228385       2.916519      -2.353228
H          -8.190663       0.646888      -2.692249
H          -1.976583       1.534796       2.733989
H         -10.527616      -0.150274      -2.975011
H         -11.053696       1.046308      -1.775097
H          -8.344976      -6.368383       0.343161
H          -9.081161      -5.225061       1.478825
H          -6.787889      -4.256866       1.874322
H          -6.147581      -5.446053       0.727784
```

VOA-d5LOH-B3PW91-C223

__Requested operations__

Run with Gaussian 2016+B.01.

`freq=(VCD,ROA) CPHF=Rdfreq B3PW91/6-31+G(2d,p) SCRF=(Solvent=chloroform,Read) guess=read test`

__Relevant magnitudes__

Datum	Value
Charge	0
Multiplicity	1
Stoichiometry	C40H60O4
Number of Basis Functions	1400
Electronic Energy (Eh)	-1860.61357268
Sum of electronic and zero-point Energies (Eh)	-1859.687846
Sum of electronic and thermal Energies (Eh)	-1859.642936
Sum of electronic and enthalpy Energies (Eh)	-1859.641992
Sum of electronic and thermal Free Energies (Eh)	-1859.779852
Number of Imaginary Frequencies	0
Mean of alpha and beta Electrons	166

__Molecular Geometry in Cartesian Coordinates__

```xyz
C          1.079501      -3.749513      -1.272966
C          0.090123      -3.565599      -2.389266
C         -1.361752      -3.446748      -1.943633
C         -2.308703      -3.251292      -3.128500
C         -3.792221      -3.194077      -2.752323
C         -4.197798      -1.979790      -1.915573
C         -5.700833      -1.911275      -1.645593
C         -6.113634      -0.708227      -0.798193
C         -7.614848      -0.616877      -0.559152
O          2.325610      -3.837789      -1.688000
O          0.760407      -3.811664      -0.088556
C         -8.470767      -0.202171      -1.791692
C         -8.988117       1.039937      -1.037258
C         -8.092087       0.650111       0.197512
C        -10.252508       0.793782      -0.197403
C         -9.347081       0.429212       1.051653
C        -10.746758       2.052471       0.527041
C         -9.832137       1.695314       1.771857
C        -11.998700       1.812102       1.384319
C        -11.088905       1.463313       2.623360
```

C	-12.503648	3.072030	2.124263
C	-11.603667	2.731338	3.342984
H	-7.306345	1.288858	0.614657
H	-8.930884	1.998996	-1.560350
H	-9.061838	2.351121	2.192642
H	-10.703927	2.993641	-0.032476
H	-9.404644	-0.509677	1.613912
H	-11.016097	0.138301	-0.630762
H	-11.141369	0.522278	3.178798
H	-12.757781	1.141635	0.970756
H	0.406664	-2.678336	-2.953005
H	0.227909	-4.407523	-3.080294
H	-1.647903	-4.348811	-1.388636
H	-1.451988	-2.612814	-1.239321
H	-2.035307	-2.331339	-3.665668
H	-2.157921	-4.073755	-3.841367
H	-4.385651	-3.197294	-3.676934
H	-4.065261	-4.113814	-2.214850
H	-3.663874	-1.987521	-0.956005
H	-3.880574	-1.062644	-2.433557
H	-6.239320	-1.887097	-2.603821
H	-6.022014	-2.834055	-1.140930
H	-5.599386	-0.752614	0.173086
H	-5.768695	0.217510	-1.283353
H	-7.958054	-1.543770	-0.080602
H	2.949002	-3.958380	-0.905496
H	-9.238761	-0.910486	-2.120932
H	-7.852131	0.055900	-2.658452
H	-13.572177	3.038925	2.356066
H	-12.295355	4.021063	1.618546
H	-10.831169	3.466196	3.593984
H	-12.172485	2.514015	4.251687
C	3.690388	-4.186806	1.490778
C	4.684751	-4.397817	2.600447
C	6.117809	-3.999735	2.256616
C	6.324837	-2.489681	2.140167
C	7.766277	-2.106042	1.809195
C	7.983661	-0.597767	1.691270
C	9.428608	-0.227507	1.355437
C	9.733496	1.272950	1.297294
C	8.998894	2.055207	0.212335
O	2.446909	-4.080657	1.907240
O	4.005993	-4.150406	0.304027
C	9.476536	3.522663	0.022780
C	8.043541	4.043348	0.243025
C	7.565604	2.573174	0.533117
C	7.164699	4.077175	-1.020360

```
C      6.644093      2.616395     -0.691303
C      5.762603      4.638489     -0.755091
C      5.246503      3.182476     -0.398435
C      4.860401      4.647620     -1.999235
C      4.335337      3.207093     -1.633681
C      3.445964      5.216282     -1.744378
C      2.931898      3.800109     -1.368247
H      7.131591      2.252168      1.484232
H      7.919274      4.878435      0.938753
H      4.785848      2.896258      0.553546
H      5.695414      5.507212     -0.090587
H      6.694081      1.765120     -1.379620
H      7.651396      4.373483     -1.956258
H      4.389753      2.344783     -2.304687
H      5.336144      4.905938     -2.949856
H      4.320129     -3.875891      3.491402
H      4.632500     -5.469223      2.839117
H      6.776929     -4.397231      3.038275
H      6.412824     -4.488073      1.320568
H      5.657460     -2.086250      1.366841
H      6.028113     -2.010098      3.084183
H      8.436927     -2.510938      2.581168
H      8.061598     -2.589570      0.866878
H      7.307643     -0.198674      0.923748
H      7.693166     -0.114464      2.635514
H     10.092381     -0.688100      2.100750
H      9.698911     -0.681450      0.390693
H      9.530335      1.732904      2.276180
H     10.812939      1.395242      1.126963
H      9.022817      1.476401     -0.720859
H      1.820993     -3.984675      1.123097
H      9.941445      3.771856     -0.937775
H     10.158097      3.833139      0.823023
H      2.973799      5.618490     -2.645473
H      3.385743      5.978867     -0.960354
H      2.565672      3.676445     -0.343402
H      2.163919      3.423522     -2.050070
```

d5LOH-B3PW91-D3B(J)

VOA-d5LOH-B3PW91-D3BJ-C21

__Requested operations__

Run with Gaussian 2016+B.01.

`freq=(VCD,ROA) CPHF=Rdfreq B3PW91/6-31+G(2d,p) SCRF=(Solvent=chloroform,Read) test`

__Relevant magnitudes__

Datum	Value
Charge	0
Multiplicity	1
Stoichiometry	C40H60O4
Number of Basis Functions	1400
Electronic Energy (Eh)	-1860.58969211
Sum of electronic and zero-point Energies (Eh)	-1859.65962
Sum of electronic and thermal Energies (Eh)	-1859.617159
Sum of electronic and enthalpy Energies (Eh)	-1859.616215
Sum of electronic and thermal Free Energies (Eh)	-1859.738612
Number of Imaginary Frequencies	1
Mean of alpha and beta Electrons	166

__Molecular Geometry in Cartesian Coordinates__

```xyz
C          3.220945       2.663384      -0.621876
C          4.597119       2.176417      -0.972052
C          4.689954       0.784487      -1.604406
C          4.239251       0.725047      -3.064846
C          2.733018       0.856527      -3.293937
C          1.930586      -0.307184      -2.717962
C          0.441863      -0.266430      -3.053089
C         -0.296795       0.921871      -2.444516
C         -1.807410       0.812485      -2.571737
O          2.468168       1.776979      -0.016589
O          2.854472       3.818333      -0.841229
C         -2.579154       1.922111      -1.807376
C         -3.128465       0.876058      -0.815361
C         -2.416040      -0.255849      -1.638185
C         -4.496642       0.309304      -1.202256
C         -3.780660      -0.809466      -2.063729
```

C	-4.994303	-0.762647	-0.226697
C	-4.292678	-1.883034	-1.099808
C	-6.365233	-1.334121	-0.603501
C	-5.669085	-2.441071	-1.481194
C	-6.863423	-2.431442	0.362653
C	-6.188351	-3.522865	-0.509880
H	-1.717864	-0.975925	-1.207850
H	-2.949818	1.056107	0.245542
H	-3.554765	-2.598350	-0.722540
H	-4.804728	-0.588897	0.837467
H	-3.942044	-0.977950	-3.133456
H	-5.241208	1.015491	-1.584234
H	-5.860622	-2.591969	-2.546961
H	-7.108467	-0.626933	-0.981482
H	5.062983	2.933620	-1.607782
H	5.146667	2.173181	-0.021020
H	5.738718	0.473249	-1.548045
H	4.128560	0.067768	-0.998438
H	4.764325	1.507669	-3.628577
H	4.571744	-0.232134	-3.488069
H	2.368743	1.806437	-2.884690
H	2.548453	0.913738	-4.375258
H	2.359151	-1.246241	-3.094357
H	2.042668	-0.333354	-1.628100
H	0.304910	-0.262371	-4.143826
H	-0.022299	-1.193152	-2.691595
H	-0.042313	1.001745	-1.378825
H	0.035121	1.860751	-2.906880
H	-2.088055	0.695361	-3.626250
H	1.567701	2.175863	0.212180
H	-3.346829	2.454375	-2.378083
H	-1.913407	2.659065	-1.349442
H	-7.952586	-2.519591	0.398915
H	-6.493655	-2.339281	1.389132
H	-5.408623	-4.117813	-0.023051
H	-6.905090	-4.209295	-0.968823
C	-0.082656	3.952647	0.704562
C	-1.336177	4.501599	1.326357
C	-2.153735	3.467237	2.088965
C	-1.481310	2.990561	3.374184
C	-2.228990	1.862830	4.090887
C	-2.459226	0.602420	3.249170
C	-1.202446	0.079776	2.560807
C	-1.410439	-1.228248	1.804186
C	-0.191304	-1.608020	0.984830
O	0.716652	4.871145	0.213445
O	0.162889	2.750072	0.645453

```
C          -0.242738      -2.955903       0.220918
C           1.085297      -3.382586       0.867460
C           1.050424      -2.101598       1.776421
C           2.345657      -2.832066       0.169897
C           2.364734      -1.611879       1.180367
C           3.639600      -3.360949       0.782219
C           3.610775      -2.201885       1.859848
C           4.900896      -2.734818       0.159919
C           4.913913      -1.641468       1.293585
C           6.219600      -3.312262       0.709605
C           6.184823      -2.297639       1.882844
H           0.950401      -2.125963       2.865834
H           1.159053      -4.405870       1.245618
H           3.555207      -2.355069       2.942670
H           3.686815      -4.430435       1.013164
H           2.419460      -0.560698       0.886901
H           2.305505      -2.739833      -0.919578
H           4.956283      -0.565397       1.109319
H           4.853556      -2.511808      -0.909357
H          -1.047777       5.347132       1.961703
H          -1.918731       4.936881       0.504174
H          -3.134090       3.899015       2.322867
H          -2.339130       2.618610       1.424979
H          -0.463139       2.657779       3.143846
H          -1.377773       3.841402       4.060610
H          -1.657214       1.590991       4.988158
H          -3.198879       2.235424       4.447313
H          -2.879596      -0.179845       3.895288
H          -3.226182       0.798248       2.487642
H          -0.835167       0.832068       1.853225
H          -0.403189      -0.049071       3.304626
H          -1.652418      -2.040416       2.505113
H          -2.275418      -1.133119       1.135291
H           0.068435      -0.766019       0.331258
H           1.526564       4.437919      -0.210539
H          -0.260169      -2.911121      -0.873359
H          -1.082907      -3.574896       0.554071
H           7.066110      -3.147581       0.036813
H           6.194978      -4.374066       0.976154
H           6.044293      -2.731171       2.878334
H           7.053129      -1.634454       1.919955
```

d5LOH-M06-2X

VOA-d5LOH-M06-2X-C477

__Requested operations__

Run with Gaussian 2016+B.01.

`freq=(VCD,ROA) CPHF=Rdfreq M062X/6-31+G(2d,p) SCRF=(Solvent=chloroform,Read) test`

__Relevant magnitudes__

Datum	Value
Charge	0
Multiplicity	1
Stoichiometry	C40H60O4
Number of Basis Functions	1400
Electronic Energy (Eh)	-1860.44892416
Sum of electronic and zero-point Energies (Eh)	-1859.514007
Sum of electronic and thermal Energies (Eh)	-1859.471373
Sum of electronic and enthalpy Energies (Eh)	-1859.470429
Sum of electronic and thermal Free Energies (Eh)	-1859.591291
Number of Imaginary Frequencies	0
Mean of alpha and beta Electrons	166

__Molecular Geometry in Cartesian Coordinates__

```xyz
C          -3.989644       -1.364282        1.664324
C          -5.399737       -1.379895        1.133316
C          -5.698576       -2.608324        0.275998
C          -4.877440       -2.631088       -1.015840
C          -5.311851       -1.594445       -2.066894
C          -4.110222       -1.037339       -2.836175
C          -3.337451       -0.016012       -1.991269
C          -1.856697        0.126308       -2.339470
C          -1.026353       -1.097696       -1.963641
O          -3.540984       -0.158383        1.941937
O          -3.327166       -2.376889        1.835865
C          -1.031842       -1.417473       -0.443696
C           0.488188       -1.198947       -0.374645
C           0.498879       -0.830400       -1.905571
C           1.324519       -2.451419       -0.664910
C           1.385292       -2.045229       -2.193475
```

C	2.819738	-2.173292	-0.523458
C	2.879991	-1.723413	-2.041667
C	3.703423	-3.385182	-0.848122
C	3.802425	-2.906876	-2.344320
C	5.209648	-3.111685	-0.665263
C	5.296103	-2.572922	-2.118623
H	0.825891	0.136390	-2.303383
H	0.868127	-0.540270	0.411765
H	3.209065	-0.739365	-2.391825
H	3.123873	-1.551338	0.325259
H	1.099694	-2.694577	-3.026152
H	0.946415	-3.402515	-0.279056
H	3.553901	-3.529774	-3.205871
H	3.324465	-4.360652	-0.536671
H	-6.054892	-1.346572	2.012067
H	-5.564120	-0.443356	0.592663
H	-5.490237	-3.505383	0.865937
H	-6.768136	-2.618653	0.038937
H	-3.821272	-2.474332	-0.757435
H	-4.925884	-3.630334	-1.460630
H	-6.033510	-2.051590	-2.751581
H	-5.836334	-0.757163	-1.587081
H	-3.457408	-1.873530	-3.117197
H	-4.430125	-0.566413	-3.771998
H	-3.833945	0.958846	-2.073509
H	-3.413025	-0.290592	-0.929224
H	-1.732624	0.341870	-3.409925
H	-1.447780	0.988745	-1.797583
H	-1.284031	-1.953953	-2.596448
H	-2.605049	-0.211547	2.290397
H	-1.377820	-2.411494	-0.146875
H	-1.584823	-0.667489	0.132568
H	5.793134	-4.023150	-0.521075
H	5.461463	-2.408037	0.134295
H	5.533991	-1.509240	-2.215839
H	5.979421	-3.139414	-2.753620
C	-0.325070	-1.257351	2.828903
C	1.127316	-1.224318	3.217577
C	1.566644	0.113411	3.799495
C	3.069748	0.173574	4.081992
C	3.960247	0.051049	2.839981
C	3.630011	1.079788	1.760630
C	4.617263	1.079477	0.596208
C	4.326923	2.149759	-0.459289
C	2.886799	2.146472	-0.963345
O	-0.769769	-2.463866	2.551653
O	-1.025103	-0.257041	2.762234

```
C           2.645659        3.027275       -2.218332
C           1.601197        3.867014       -1.458386
C           1.870167        3.011715       -0.167299
C           0.177746        3.298847       -1.540192
C           0.441062        2.462385       -0.220624
C          -0.855385        4.150705       -0.794172
C          -0.591154        3.313212        0.523153
C          -2.273303        3.574172       -0.870007
C          -2.004322        2.719617        0.425534
C          -3.316987        4.402732       -0.087085
C          -3.077683        3.532872        1.176226
H           2.215800        3.429240        0.781696
H           1.696678        4.953626       -1.507835
H          -0.293120        3.734391        1.487852
H          -0.754291        5.237022       -0.878293
H           0.322788        1.379998       -0.135213
H          -0.128992        2.872730       -2.501128
H          -2.081494        1.629870        0.471975
H          -2.586579        3.155201       -1.829525
H           1.308292       -2.048744        3.915315
H           1.689232       -1.476896        2.308112
H           1.274765        0.917735        3.116183
H           1.015646        0.290863        4.728851
H           3.287573        1.129622        4.572821
H           3.336303       -0.612182        4.799580
H           5.005802        0.175967        3.147262
H           3.893050       -0.960991        2.417646
H           2.619527        0.895476        1.368420
H           3.600573        2.079528        2.217852
H           5.636043        1.211960        0.981189
H           4.595723        0.093933        0.110232
H           4.560730        3.147576       -0.060756
H           4.998055        1.994176       -1.314108
H           2.546795        1.108117       -1.075157
H          -1.731014       -2.419688        2.284593
H           2.277582        2.515355       -3.113424
H           3.538417        3.600541       -2.486743
H          -4.330603        4.318959       -0.483899
H          -3.069464        5.463725        0.009299
H          -2.721323        4.056820        2.067559
H          -3.948383        2.933603        1.449845
```

VOA-d5LOH-M06-2X-C21

__Requested operations__

Run with Gaussian 2016+B.01.

`freq=(VCD,ROA) CPHF=Rdfreq M062X/6-31+G(2d,p) SCRF=(Solvent=chloroform,Read) test`

__Relevant magnitudes__

Datum	Value
Charge	0
Multiplicity	1
Stoichiometry	C40H60O4
Number of Basis Functions	1400
Electronic Energy (Eh)	-1860.44833547
Sum of electronic and zero-point Energies (Eh)	-1859.514002
Sum of electronic and thermal Energies (Eh)	-1859.471223
Sum of electronic and enthalpy Energies (Eh)	-1859.470279
Sum of electronic and thermal Free Energies (Eh)	-1859.593035
Number of Imaginary Frequencies	0
Mean of alpha and beta Electrons	166

__Molecular Geometry in Cartesian Coordinates__

```xyz
C          3.205401       2.650261      -0.676505
C          4.592512       2.142438      -0.959703
C          4.682449       0.727532      -1.541237
C          4.247705       0.629388      -3.006233
C          2.740054       0.758824      -3.241303
C          1.944067      -0.394962      -2.632046
C          0.454362      -0.370274      -2.971217
C         -0.290332       0.826248      -2.381949
C         -1.800637       0.720615      -2.542531
O          2.412297       1.768621      -0.110407
O          2.860093       3.801652      -0.910225
C         -2.572287       1.839345      -1.790936
C         -3.109314       0.804129      -0.777398
C         -2.424844      -0.339138      -1.609803
C         -4.492383       0.257419      -1.141432
C         -3.806927      -0.861244      -2.026668
C         -4.988626      -0.818328      -0.168123
C         -4.324668      -1.939021      -1.069452
C         -6.376888      -1.358128      -0.528755
C         -5.719330      -2.459884      -1.440585
C         -6.871984      -2.464621       0.428978
C         -6.243387      -3.551680      -0.483640
```

H	-1.741202	-1.079521	-1.185352
H	-2.905862	0.987213	0.279137
H	-3.595893	-2.676141	-0.716069
H	-4.776551	-0.661235	0.894498
H	-3.984694	-1.012602	-3.095565
H	-5.233869	0.976243	-1.502998
H	-5.929128	-2.582289	-2.505252
H	-7.114164	-0.631976	-0.877635
H	5.084358	2.872497	-1.606614
H	5.105575	2.168352	0.010149
H	5.726164	0.406716	-1.458388
H	4.099651	0.038424	-0.920682
H	4.778827	1.395916	-3.584202
H	4.575510	-0.340417	-3.401120
H	2.374811	1.718892	-2.855932
H	2.554484	0.782911	-4.322930
H	2.380612	-1.339164	-2.985560
H	2.052524	-0.389482	-1.540055
H	0.320530	-0.381519	-4.061688
H	-0.006222	-1.292878	-2.593659
H	-0.060968	0.908765	-1.308319
H	0.054612	1.762317	-2.841307
H	-2.059820	0.599412	-3.601516
H	1.519295	2.182528	0.076155
H	-3.352771	2.348948	-2.363072
H	-1.905876	2.588853	-1.353399
H	-7.959589	-2.533181	0.492543
H	-6.468989	-2.399273	1.443568
H	-5.468275	-4.175774	-0.030015
H	-6.989324	-4.204161	-0.941677
C	-0.118096	4.049691	0.568388
C	-1.364329	4.611246	1.199770
C	-2.156637	3.570959	1.984664
C	-1.430531	3.107419	3.247507
C	-2.155244	1.995701	4.012980
C	-2.416808	0.722145	3.197961
C	-1.183889	0.200722	2.461219
C	-1.389887	-1.163306	1.805905
C	-0.175968	-1.594054	0.999123
O	0.716598	4.961825	0.123410
O	0.090722	2.849101	0.460582
C	-0.242255	-2.987578	0.322373
C	1.100755	-3.368531	0.967038
C	1.066326	-2.047232	1.816048
C	2.340795	-2.831795	0.219697
C	2.381141	-1.586432	1.198364
C	3.651956	-3.339549	0.810616

```
C          3.625543       -2.178524        1.884897
C          4.891043       -2.691100        0.159921
C          4.931364       -1.627351        1.319708
C          6.228142       -3.274179        0.651941
C          6.190311       -2.331573        1.883896
H          0.961096       -2.021912        2.904824
H          1.199091       -4.370814        1.389559
H          3.563812       -2.330001        2.966830
H          3.724198       -4.406289        1.043927
H          2.448927       -0.542196        0.878670
H          2.266506       -2.767727       -0.870419
H          4.999568       -0.548268        1.161282
H          4.800346       -2.442896       -0.901252
H         -1.068412        5.460645        1.824099
H         -1.958359        5.029179        0.377903
H         -3.129903        3.996057        2.253748
H         -2.352624        2.719210        1.325828
H         -0.425699        2.762988        2.977526
H         -1.295606        3.968274        3.914175
H         -1.550435        1.734535        4.890108
H         -3.109149        2.378856        4.396536
H         -2.794659       -0.055730        3.873436
H         -3.220155        0.901930        2.470811
H         -0.886716        0.919382        1.685475
H         -0.337922        0.143044        3.162082
H         -1.602819       -1.922202        2.572850
H         -2.270542       -1.131602        1.148407
H          0.088349       -0.794746        0.291885
H          1.508137        4.518977       -0.299573
H         -0.284227       -3.016748       -0.771767
H         -1.072049       -3.580482        0.721478
H          7.055996       -3.037322       -0.020271
H          6.235119       -4.349901        0.849261
H          6.018529       -2.828176        2.843117
H          7.066101       -1.687625        1.980687
```

VOA-d5LOH-M06-2X-C460

__Requested operations__

Run with Gaussian 2016+B.01.

`freq=(VCD,ROA) CPHF=Rdfreq M062X/6-31+G(2d,p) SCRF=(Solvent=chloroform,Read) test`

__Relevant magnitudes__

Datum	Value
Charge	0
Multiplicity	1
Stoichiometry	C40H60O4
Number of Basis Functions	1400
Electronic Energy (Eh)	-1860.44790879
Sum of electronic and zero-point Energies (Eh)	-1859.513533
Sum of electronic and thermal Energies (Eh)	-1859.470796
Sum of electronic and enthalpy Energies (Eh)	-1859.469852
Sum of electronic and thermal Free Energies (Eh)	-1859.590628
Number of Imaginary Frequencies	0
Mean of alpha and beta Electrons	166

__Molecular Geometry in Cartesian Coordinates__

```xyz
C          -4.284314       -2.623516        0.720292
C          -5.605321       -2.932612        0.070822
C          -6.363502       -1.729328       -0.487905
C          -5.893694       -1.208900       -1.852737
C          -4.524430       -0.516592       -1.859496
C          -3.325732       -1.448315       -2.058728
C          -2.000215       -0.767078       -1.728595
C          -0.803733       -1.714322       -1.722991
C           0.505766       -0.973855       -1.502088
O          -3.509188       -3.676309        0.848848
O          -3.967167       -1.508410        1.113824
C           0.963294       -0.109367       -2.710316
C           2.273811       -0.915366       -2.808383
C           1.792652       -1.822941       -1.615949
C           3.404612       -0.357925       -1.930794
C           2.939745       -1.301071       -0.747044
C           4.708046       -1.150308       -2.047981
C           4.230661       -2.117123       -0.888837
C           5.815108       -0.606494       -1.131364
C           5.368347       -1.606510       -0.000359
C           7.153364       -1.361894       -1.261465
C           6.689698       -2.386369       -0.191683
H           1.658580       -2.906545       -1.677587
H           2.561214       -1.292786       -3.791709
H           4.118560       -3.201157       -0.983971
H           5.007830       -1.473529       -3.049229
H           2.682735       -0.954094        0.259612
H           3.493117        0.732062       -1.868957
```

H	5.112743	-1.305662	1.018408
H	5.856789	0.478966	-1.010899
H	-5.431479	-3.695937	-0.694355
H	-6.197279	-3.422506	0.854245
H	-7.413460	-2.024003	-0.579590
H	-6.327947	-0.914189	0.243854
H	-5.896637	-2.033552	-2.579145
H	-6.650030	-0.495957	-2.198480
H	-4.504174	0.234469	-2.658759
H	-4.400563	0.033677	-0.917776
H	-3.420711	-2.352329	-1.445968
H	-3.311898	-1.805435	-3.097078
H	-1.821594	0.050730	-2.441942
H	-2.076056	-0.292904	-0.740642
H	-0.939150	-2.470206	-0.935361
H	-0.750765	-2.262296	-2.675419
H	0.450235	-0.420198	-0.554764
H	-2.649975	-3.431640	1.307831
H	1.081958	0.964837	-2.539173
H	0.310719	-0.251478	-3.577547
H	8.009897	-0.769964	-0.932702
H	7.367183	-1.759370	-2.257560
H	6.556655	-3.413221	-0.543551
H	7.323894	-2.404897	0.696422
C	-0.817820	-1.932023	2.125396
C	0.562613	-1.605139	2.613532
C	0.565531	-0.541346	3.717185
C	1.964530	-0.290813	4.284993
C	3.010930	0.097754	3.237193
C	2.614839	1.300567	2.383855
C	3.712972	1.707912	1.404363
C	3.341402	2.885277	0.501380
C	2.000095	2.719240	-0.205263
O	-1.546253	-0.875295	1.849517
O	-1.225698	-3.078602	1.985797
C	1.746263	3.706808	-1.372168
C	0.399574	4.109629	-0.755238
C	0.732026	3.257889	0.523549
C	-0.762194	3.149291	-1.075358
C	-0.506061	2.393890	0.294180
C	-2.099313	3.664549	-0.549509
C	-1.805834	2.997658	0.855664
C	-3.243857	2.664165	-0.781804
C	-3.013842	2.080016	0.661007
C	-4.632063	3.172274	-0.352755
C	-4.354003	2.715010	1.104023
H	0.892968	3.683598	1.518058

```
H          0.144227        5.171275       -0.738084
H         -1.730372        3.539881        1.803131
H         -2.325258        4.723858       -0.705604
H         -0.395513        1.310158        0.405217
H         -0.756347        2.674478       -2.062154
H         -2.883101        1.019593        0.876823
H         -3.180445        2.075010       -1.698959
H          1.034881       -2.533299        2.942929
H          1.117249       -1.240839        1.738795
H          0.143091        0.386176        3.317881
H         -0.097843       -0.863132        4.527294
H          1.893328        0.506384        5.034493
H          2.303811       -1.188899        4.815145
H          3.958495        0.312653        3.746210
H          3.213666       -0.758539        2.578571
H          1.701294        1.067217        1.818037
H          2.360099        2.147570        3.037200
H          4.629836        1.942666        1.959762
H          3.951470        0.845406        0.767939
H          3.324708        3.820548        1.078577
H          4.125170        3.007214       -0.258494
H          1.870628        1.668041       -0.496784
H         -2.472761       -1.147898        1.569092
H          1.719072        3.294222       -2.386327
H          2.467756        4.530096       -1.349218
H         -5.442111        2.601965       -0.815505
H         -4.819774        4.238081       -0.511978
H         -4.241249        3.521293        1.834495
H         -5.080622        1.996921        1.489631
```

VOA-d5LOH-M06-2X-C310

__Requested operations__

Run with Gaussian 2016+B.01.

`freq=(VCD,ROA) CPHF=Rdfreq M062X/6-31+G(2d,p) SCRF=(Solvent=chloroform,Read) test`

__Relevant magnitudes__

Datum	Value
Charge	0
Multiplicity	1

Stoichiometry	C40H60O4	
Number of Basis Functions	1400	
Electronic Energy (Eh)	-1860.44889909	
Sum of electronic and zero-point Energies (Eh)	-1859.513513	
Sum of electronic and thermal Energies (Eh)	-1859.470878	
Sum of electronic and enthalpy Energies (Eh)	-1859.469934	
Sum of electronic and thermal Free Energies (Eh)	-1859.590101	
Number of Imaginary Frequencies	0	
Mean of alpha and beta Electrons	166	

__Molecular Geometry in Cartesian Coordinates__

```xyz
C          -3.764750       -2.489621       -0.277508
C          -5.183077       -2.340921        0.195368
C          -5.535529       -0.853885        0.374904
C          -5.148843       -0.016039       -0.841629
C          -5.519601        1.458007       -0.707126
C          -5.203701        2.278827       -1.963455
C          -3.763981        2.144917       -2.471092
C          -2.697702        2.527152       -1.446376
C          -1.286023        2.307146       -1.967409
O          -2.862553       -2.076012        0.587362
O          -3.476933       -2.914244       -1.387703
C          -0.875248        0.811174       -2.084224
C           0.284609        1.038803       -1.096881
C          -0.162767        2.539954       -0.930015
C           1.598221        1.512053       -1.729825
C           1.154090        3.020456       -1.551181
C           2.679721        1.708210       -0.658435
C           2.265799        3.227726       -0.521733
C           4.036215        2.124630       -1.227109
C           3.607530        3.639062       -1.150572
C           5.098616        2.388115       -0.134303
C           4.721000        3.893735       -0.114459
H          -0.468081        2.988321        0.020289
H           0.388449        0.326097       -0.277425
H           1.991421        3.716762        0.418351
H           2.673752        0.991336        0.166521
H           1.079740        3.752325       -2.361219
H           1.906489        1.056464       -2.677026
H           3.557438        4.308220       -2.012148
H           4.371356        1.625784       -2.139544
H          -5.826032       -2.804484       -0.554480
H          -5.296991       -2.875057        1.142707
H          -6.612108       -0.777171        0.558947
H          -5.030387       -0.466103        1.266263
```

H	-4.063650	-0.098268	-0.997116
H	-5.617685	-0.435772	-1.743023
H	-6.590934	1.543314	-0.487801
H	-4.996115	1.880568	0.159583
H	-5.886869	1.973865	-2.765871
H	-5.416310	3.335444	-1.758944
H	-3.585532	1.115922	-2.810092
H	-3.642189	2.778399	-3.358427
H	-2.825159	3.578795	-1.153000
H	-2.817745	1.933698	-0.528925
H	-1.136972	2.882058	-2.889604
H	-1.949927	-2.151307	0.187718
H	-0.597313	0.440301	-3.076512
H	-1.648770	0.151175	-1.670651
H	6.124427	2.198508	-0.455771
H	4.923190	1.844432	0.799229
H	4.382457	4.299576	0.842973
H	5.520724	4.530249	-0.498597
C	-0.222652	-2.285183	-1.720095
C	1.144557	-2.120655	-2.317594
C	1.976403	-3.381865	-2.025184
C	3.320026	-3.409034	-2.757470
C	4.356507	-2.375952	-2.300008
C	4.715922	-2.492951	-0.807817
C	3.992306	-1.457609	0.052682
C	4.018584	-1.744394	1.549940
C	3.197030	-0.741287	2.350508
O	-1.152178	-2.623908	-2.585005
O	-0.439078	-2.162172	-0.521256
C	3.015330	-1.121070	3.844501
C	1.505181	-1.358429	3.617697
C	1.673323	-0.879921	2.127655
C	0.638573	-0.133867	3.924788
C	0.858378	0.384517	2.443747
C	-0.847382	-0.372316	3.619063
C	-0.632312	0.201864	2.161064
C	-1.744090	0.821900	3.951345
C	-1.470040	1.435589	2.528035
C	-3.221486	0.598302	3.546083
C	-2.958556	1.269665	2.170974
H	1.344091	-1.458495	1.259082
H	1.105976	-2.333823	3.903421
H	-0.909648	-0.322197	1.243116
H	-1.239831	-1.369132	3.842315
H	1.303763	1.348417	2.172899
H	0.874183	0.434601	4.829702
H	-1.008027	2.410317	2.356800

```
H          -1.560367        1.345361        4.892037
H           1.052113       -1.968903       -3.395951
H           1.604648       -1.234413       -1.871397
H           2.122155       -3.470059       -0.941427
H           1.395475       -4.258034       -2.334621
H           3.754896       -4.407079       -2.624209
H           3.137006       -3.291839       -3.832673
H           5.250301       -2.510529       -2.917287
H           3.996118       -1.358220       -2.507891
H           4.468946       -3.503366       -0.453425
H           5.796051       -2.378683       -0.664989
H           4.430257       -0.469500       -0.143947
H           2.944955       -1.387039       -0.267144
H           3.614096       -2.752096        1.730666
H           5.053206       -1.752537        1.919469
H           3.580063        0.272701        2.179184
H          -2.023805       -2.760217       -2.112445
H           3.241728       -0.331934        4.567226
H           3.572950       -2.019481        4.124420
H          -3.939192        1.139056        4.165633
H          -3.518319       -0.454377        3.505679
H          -3.143332        0.648510        1.288339
H          -3.486266        2.219397        2.052715
```

d5LOH-M06-2X-D3

VOA-d5LOH-M06-2X-D3-C477

__Requested operations__

Run with Gaussian 2016+B.01.

`freq=(VCD,ROA) CPHF=Rdfreq M062X/6-31+G(2d,p) SCRF=(Solvent=chloroform,Read) test`

__Relevant magnitudes__

Datum	Value
Charge	0
Multiplicity	1
Stoichiometry	C40H60O4
Number of Basis Functions	1400
Electronic Energy (Eh)	-1860.44892416
Sum of electronic and zero-point Energies (Eh)	-1859.514007
Sum of electronic and thermal Energies (Eh)	-1859.471373
Sum of electronic and enthalpy Energies (Eh)	-1859.470429
Sum of electronic and thermal Free Energies (Eh)	-1859.591291
Number of Imaginary Frequencies	0
Mean of alpha and beta Electrons	166

__Molecular Geometry in Cartesian Coordinates__

```xyz
C          -3.989644      -1.364282       1.664324
C          -5.399737      -1.379895       1.133316
C          -5.698576      -2.608324       0.275998
C          -4.877440      -2.631088      -1.015840
C          -5.311851      -1.594445      -2.066894
C          -4.110222      -1.037339      -2.836175
C          -3.337451      -0.016012      -1.991269
C          -1.856697       0.126308      -2.339470
C          -1.026353      -1.097696      -1.963641
O          -3.540984      -0.158383       1.941937
O          -3.327166      -2.376889       1.835865
C          -1.031842      -1.417473      -0.443696
C           0.488188      -1.198947      -0.374645
C           0.498879      -0.830400      -1.905571
C           1.324519      -2.451419      -0.664910
C           1.385292      -2.045229      -2.193475
```

C	2.819738	-2.173292	-0.523458
C	2.879991	-1.723413	-2.041667
C	3.703423	-3.385182	-0.848122
C	3.802425	-2.906876	-2.344320
C	5.209648	-3.111685	-0.665263
C	5.296103	-2.572922	-2.118623
H	0.825891	0.136390	-2.303383
H	0.868127	-0.540270	0.411765
H	3.209065	-0.739365	-2.391825
H	3.123873	-1.551338	0.325259
H	1.099694	-2.694577	-3.026152
H	0.946415	-3.402515	-0.279056
H	3.553901	-3.529774	-3.205871
H	3.324465	-4.360652	-0.536671
H	-6.054892	-1.346572	2.012067
H	-5.564120	-0.443356	0.592663
H	-5.490237	-3.505383	0.865937
H	-6.768136	-2.618653	0.038937
H	-3.821272	-2.474332	-0.757435
H	-4.925884	-3.630334	-1.460630
H	-6.033510	-2.051590	-2.751581
H	-5.836334	-0.757163	-1.587081
H	-3.457408	-1.873530	-3.117197
H	-4.430125	-0.566413	-3.771998
H	-3.833945	0.958846	-2.073509
H	-3.413025	-0.290592	-0.929224
H	-1.732624	0.341870	-3.409925
H	-1.447780	0.988745	-1.797583
H	-1.284031	-1.953953	-2.596448
H	-2.605049	-0.211547	2.290397
H	-1.377820	-2.411494	-0.146875
H	-1.584823	-0.667489	0.132568
H	5.793134	-4.023150	-0.521075
H	5.461463	-2.408037	0.134295
H	5.533991	-1.509240	-2.215839
H	5.979421	-3.139414	-2.753620
C	-0.325070	-1.257351	2.828903
C	1.127316	-1.224318	3.217577
C	1.566644	0.113411	3.799495
C	3.069748	0.173574	4.081992
C	3.960247	0.051049	2.839981
C	3.630011	1.079788	1.760630
C	4.617263	1.079477	0.596208
C	4.326923	2.149759	-0.459289
C	2.886799	2.146472	-0.963345
O	-0.769769	-2.463866	2.551653
O	-1.025103	-0.257041	2.762234

```
C           2.645659        3.027275       -2.218332
C           1.601197        3.867014       -1.458386
C           1.870167        3.011715       -0.167299
C           0.177746        3.298847       -1.540192
C           0.441062        2.462385       -0.220624
C          -0.855385        4.150705       -0.794172
C          -0.591154        3.313212        0.523153
C          -2.273303        3.574172       -0.870007
C          -2.004322        2.719617        0.425534
C          -3.316987        4.402732       -0.087085
C          -3.077683        3.532872        1.176226
H           2.215800        3.429240        0.781696
H           1.696678        4.953626       -1.507835
H          -0.293120        3.734391        1.487852
H          -0.754291        5.237022       -0.878293
H           0.322788        1.379998       -0.135213
H          -0.128992        2.872730       -2.501128
H          -2.081494        1.629870        0.471975
H          -2.586579        3.155201       -1.829525
H           1.308292       -2.048744        3.915315
H           1.689232       -1.476896        2.308112
H           1.274765        0.917735        3.116183
H           1.015646        0.290863        4.728851
H           3.287573        1.129622        4.572821
H           3.336303       -0.612182        4.799580
H           5.005802        0.175967        3.147262
H           3.893050       -0.960991        2.417646
H           2.619527        0.895476        1.368420
H           3.600573        2.079528        2.217852
H           5.636043        1.211960        0.981189
H           4.595723        0.093933        0.110232
H           4.560730        3.147576       -0.060756
H           4.998055        1.994176       -1.314108
H           2.546795        1.108117       -1.075157
H          -1.731014       -2.419688        2.284593
H           2.277582        2.515355       -3.113424
H           3.538417        3.600541       -2.486743
H          -4.330603        4.318959       -0.483899
H          -3.069464        5.463725        0.009299
H          -2.721323        4.056820        2.067559
H          -3.948383        2.933603        1.449845
```

VOA-d5LOH-M06-2X-D3-C359

__Requested operations__

Run with Gaussian 2016+B.01.

`freq=(VCD,ROA) CPHF=Rdfreq M062X/6-31+G(2d,p) SCRF=(Solvent=chloroform,Read) test`

__Relevant magnitudes__

Datum	Value
Charge	0
Multiplicity	1
Stoichiometry	C40H60O4
Number of Basis Functions	1400
Electronic Energy (Eh)	-1860.44661732
Sum of electronic and zero-point Energies (Eh)	-1859.511289
Sum of electronic and thermal Energies (Eh)	-1859.468708
Sum of electronic and enthalpy Energies (Eh)	-1859.467764
Sum of electronic and thermal Free Energies (Eh)	-1859.588715
Number of Imaginary Frequencies	0
Mean of alpha and beta Electrons	166

__Molecular Geometry in Cartesian Coordinates__

```xyz
C          -3.435670       -2.485409        1.134903
C          -4.927447       -2.538216        1.310812
C          -5.728837       -1.555623        0.449028
C          -5.769656       -1.930257       -1.036228
C          -4.468707       -1.693741       -1.809719
C          -4.155387       -0.211994       -2.020340
C          -2.842974        0.035592       -2.766712
C          -1.598305       -0.172505       -1.905545
C          -0.312272        0.143213       -2.656975
O          -2.933408       -1.271176        1.143740
O          -2.745670       -3.491906        1.030490
C           0.089700       -0.963893       -3.670513
C           1.284583       -1.366045       -2.775122
C           0.952363       -0.146745       -1.828030
C           2.593317       -0.697321       -3.201581
C           2.230874        0.557121       -2.307053
C           3.775018       -1.015961       -2.278306
C           3.431201        0.257620       -1.403316
C           5.082575       -0.350155       -2.719673
C           4.725355        0.931347       -1.877110
C           6.261979       -0.631391       -1.760962
C           5.930142        0.653599       -0.955117
```

H	0.805954	-0.205355	-0.746207
H	1.330581	-2.409685	-2.454171
H	3.253901	0.248522	-0.323320
H	3.846084	-2.040139	-1.897753
H	2.128775	1.581335	-2.680731
H	2.803832	-0.658964	-4.274490
H	4.628621	1.936480	-2.293398
H	5.299201	-0.340824	-3.789838
H	-5.243530	-3.568649	1.134481
H	-5.101140	-2.314843	2.370331
H	-6.751644	-1.546400	0.837238
H	-5.333150	-0.543706	0.587428
H	-6.058897	-2.985264	-1.119116
H	-6.566681	-1.352999	-1.520995
H	-3.628331	-2.193709	-1.313133
H	-4.555673	-2.170408	-2.794036
H	-4.986301	0.234869	-2.581090
H	-4.112649	0.308913	-1.054926
H	-2.795828	-0.614949	-3.651281
H	-2.830585	1.064782	-3.147102
H	-1.660740	0.461108	-1.009107
H	-1.548937	-1.210529	-1.541878
H	-0.363925	1.160136	-3.068282
H	-1.933667	-1.319096	1.099705
H	0.382306	-0.617902	-4.665847
H	-0.671837	-1.740847	-3.783050
H	7.239416	-0.596081	-2.245425
H	6.181742	-1.574036	-1.211799
H	5.678168	0.514635	0.100754
H	6.712011	1.412037	-1.027684
C	0.381547	-2.390849	0.927525
C	1.872931	-2.319218	0.756084
C	2.660733	-3.248268	1.682759
C	2.562764	-2.864733	3.161664
C	3.039717	-1.430242	3.465372
C	1.890673	-0.458214	3.744625
C	2.340342	0.980312	4.010150
C	2.887763	1.730911	2.790494
C	1.888008	1.868684	1.650957
O	-0.120270	-3.601276	0.840679
O	-0.309885	-1.396396	1.106633
C	2.272537	2.877035	0.534375
C	0.928286	3.601958	0.715660
C	0.604244	2.689298	1.951920
C	-0.243103	2.956559	-0.049565
C	-0.621622	2.119357	1.240794
C	-1.541854	3.754721	0.085123

```
C          -1.893495        2.961322        1.408667
C          -2.732034        3.088975       -0.620296
C          -3.099396        2.326649        0.709035
C          -4.046898        3.889324       -0.513594
C          -4.395325        3.160867        0.811890
H           0.486773        3.063407        2.972672
H           0.938349        4.693760        0.741781
H          -2.062765        3.419109        2.387989
H          -1.467785        4.843854        0.007028
H          -0.740139        1.032277        1.261414
H          -0.014688        2.522594       -1.029762
H          -3.195753        1.241815        0.792085
H          -2.520192        2.599765       -1.573511
H           2.074895       -2.579989       -0.288697
H           2.167143       -1.277384        0.895505
H           2.321990       -4.278202        1.536340
H           3.711236       -3.208693        1.372704
H           1.524076       -2.982342        3.500676
H           3.150566       -3.587669        3.735114
H           3.702331       -1.434135        4.338431
H           3.650743       -1.064253        2.628248
H           1.169003       -0.478416        2.918157
H           1.346952       -0.824637        4.624312
H           1.489464        1.545090        4.409714
H           3.102864        0.976768        4.799599
H           3.192631        2.736800        3.109405
H           3.796670        1.244108        2.409854
H           1.628899        0.879113        1.253235
H          -1.117882       -3.557861        0.925557
H           2.466510        2.474124       -0.463601
H           3.123871        3.496539        0.834530
H          -4.750944        3.663369       -1.317578
H          -3.923925        4.974526       -0.454278
H          -4.460349        3.791243        1.703304
H          -5.305790        2.560817        0.745675
```

VOA-d5LOH-M06-2X-D3-C310

__Requested operations__

Run with Gaussian 2016+B.01.

`freq=(VCD,ROA) CPHF=Rdfreq M062X/6-31+G(2d,p) SCRF=(Solvent=chloroform,Read) test`

__Relevant magnitudes__

Datum	Value
Charge	0
Multiplicity	1
Stoichiometry	C40H60O4
Number of Basis Functions	1400
Electronic Energy (Eh)	-1860.44889909
Sum of electronic and zero-point Energies (Eh)	-1859.513513
Sum of electronic and thermal Energies (Eh)	-1859.470878
Sum of electronic and enthalpy Energies (Eh)	-1859.469934
Sum of electronic and thermal Free Energies (Eh)	-1859.590101
Number of Imaginary Frequencies	0
Mean of alpha and beta Electrons	166

__Molecular Geometry in Cartesian Coordinates__

```xyz
C          -3.764750       -2.489621       -0.277508
C          -5.183077       -2.340921        0.195368
C          -5.535529       -0.853885        0.374904
C          -5.148843       -0.016039       -0.841629
C          -5.519601        1.458007       -0.707126
C          -5.203701        2.278827       -1.963455
C          -3.763981        2.144917       -2.471092
C          -2.697702        2.527152       -1.446376
C          -1.286023        2.307146       -1.967409
O          -2.862553       -2.076012        0.587362
O          -3.476933       -2.914244       -1.387703
C          -0.875248        0.811174       -2.084224
C           0.284609        1.038803       -1.096881
C          -0.162767        2.539954       -0.930015
C           1.598221        1.512053       -1.729825
C           1.154090        3.020456       -1.551181
C           2.679721        1.708210       -0.658435
C           2.265799        3.227726       -0.521733
C           4.036215        2.124630       -1.227109
C           3.607530        3.639062       -1.150572
C           5.098616        2.388115       -0.134303
C           4.721000        3.893735       -0.114459
H          -0.468081        2.988321        0.020289
H           0.388449        0.326097       -0.277425
H           1.991421        3.716762        0.418351
H           2.673752        0.991336        0.166521
H           1.079740        3.752325       -2.361219
H           1.906489        1.056464       -2.677026
```

H	3.557438	4.308220	-2.012148
H	4.371356	1.625784	-2.139544
H	-5.826032	-2.804484	-0.554480
H	-5.296991	-2.875057	1.142707
H	-6.612108	-0.777171	0.558947
H	-5.030387	-0.466103	1.266263
H	-4.063650	-0.098268	-0.997116
H	-5.617685	-0.435772	-1.743023
H	-6.590934	1.543314	-0.487801
H	-4.996115	1.880568	0.159583
H	-5.886869	1.973865	-2.765871
H	-5.416310	3.335444	-1.758944
H	-3.585532	1.115922	-2.810092
H	-3.642189	2.778399	-3.358427
H	-2.825159	3.578795	-1.153000
H	-2.817745	1.933698	-0.528925
H	-1.136972	2.882058	-2.889604
H	-1.949927	-2.151307	0.187718
H	-0.597313	0.440301	-3.076512
H	-1.648770	0.151175	-1.670651
H	6.124427	2.198508	-0.455771
H	4.923190	1.844432	0.799229
H	4.382457	4.299576	0.842973
H	5.520724	4.530249	-0.498597
C	-0.222652	-2.285183	-1.720095
C	1.144557	-2.120655	-2.317594
C	1.976403	-3.381865	-2.025184
C	3.320026	-3.409034	-2.757470
C	4.356507	-2.375952	-2.300008
C	4.715922	-2.492951	-0.807817
C	3.992306	-1.457609	0.052682
C	4.018584	-1.744394	1.549940
C	3.197030	-0.741287	2.350508
O	-1.152178	-2.623908	-2.585005
O	-0.439078	-2.162172	-0.521256
C	3.015330	-1.121070	3.844501
C	1.505181	-1.358429	3.617697
C	1.673323	-0.879921	2.127655
C	0.638573	-0.133867	3.924788
C	0.858378	0.384517	2.443747
C	-0.847382	-0.372316	3.619063
C	-0.632312	0.201864	2.161064
C	-1.744090	0.821900	3.951345
C	-1.470040	1.435589	2.528035
C	-3.221486	0.598302	3.546083
C	-2.958556	1.269665	2.170974
H	1.344091	-1.458495	1.259082

```
H          1.105976       -2.333823        3.903421
H         -0.909648       -0.322197        1.243116
H         -1.239831       -1.369132        3.842315
H          1.303763        1.348417        2.172899
H          0.874183        0.434601        4.829702
H         -1.008027        2.410317        2.356800
H         -1.560367        1.345361        4.892037
H          1.052113       -1.968903       -3.395951
H          1.604648       -1.234413       -1.871397
H          2.122155       -3.470059       -0.941427
H          1.395475       -4.258034       -2.334621
H          3.754896       -4.407079       -2.624209
H          3.137006       -3.291839       -3.832673
H          5.250301       -2.510529       -2.917287
H          3.996118       -1.358220       -2.507891
H          4.468946       -3.503366       -0.453425
H          5.796051       -2.378683       -0.664989
H          4.430257       -0.469500       -0.143947
H          2.944955       -1.387039       -0.267144
H          3.614096       -2.752096        1.730666
H          5.053206       -1.752537        1.919469
H          3.580063        0.272701        2.179184
H         -2.023805       -2.760217       -2.112445
H          3.241728       -0.331934        4.567226
H          3.572950       -2.019481        4.124420
H         -3.939192        1.139056        4.165633
H         -3.518319       -0.454377        3.505679
H         -3.143332        0.648510        1.288339
H         -3.486266        2.219397        2.052715
```

VOA-d5LOH-M06-2X-D3-C460

__Requested operations__

Run with Gaussian 2016+B.01.

`freq=(VCD,ROA) CPHF=Rdfreq M062X/6-31+G(2d,p) SCRF=(Solvent=chloroform,Read) test`

__Relevant magnitudes__

Datum	Value
Charge	0
Multiplicity	1

Stoichiometry	C40H60O4
Number of Basis Functions	1400
Electronic Energy (Eh)	-1860.44790879
Sum of electronic and zero-point Energies (Eh)	-1859.513533
Sum of electronic and thermal Energies (Eh)	-1859.470796
Sum of electronic and enthalpy Energies (Eh)	-1859.469852
Sum of electronic and thermal Free Energies (Eh)	-1859.590628
Number of Imaginary Frequencies	0
Mean of alpha and beta Electrons	166

__Molecular Geometry in Cartesian Coordinates__

```xyz
C          -4.284314       -2.623516        0.720292
C          -5.605321       -2.932612        0.070822
C          -6.363502       -1.729328       -0.487905
C          -5.893694       -1.208900       -1.852737
C          -4.524430       -0.516592       -1.859496
C          -3.325732       -1.448315       -2.058728
C          -2.000215       -0.767078       -1.728595
C          -0.803733       -1.714322       -1.722991
C           0.505766       -0.973855       -1.502088
O          -3.509188       -3.676309        0.848848
O          -3.967167       -1.508410        1.113824
C           0.963294       -0.109367       -2.710316
C           2.273811       -0.915366       -2.808383
C           1.792652       -1.822941       -1.615949
C           3.404612       -0.357925       -1.930794
C           2.939745       -1.301071       -0.747044
C           4.708046       -1.150308       -2.047981
C           4.230661       -2.117123       -0.888837
C           5.815108       -0.606494       -1.131364
C           5.368347       -1.606510       -0.000359
C           7.153364       -1.361894       -1.261465
C           6.689698       -2.386369       -0.191683
H           1.658580       -2.906545       -1.677587
H           2.561214       -1.292786       -3.791709
H           4.118560       -3.201157       -0.983971
H           5.007830       -1.473529       -3.049229
H           2.682735       -0.954094        0.259612
H           3.493117        0.732062       -1.868957
H           5.112743       -1.305662        1.018408
H           5.856789        0.478966       -1.010899
H          -5.431479       -3.695937       -0.694355
H          -6.197279       -3.422506        0.854245
H          -7.413460       -2.024003       -0.579590
H          -6.327947       -0.914189        0.243854
```

H	-5.896637	-2.033552	-2.579145
H	-6.650030	-0.495957	-2.198480
H	-4.504174	0.234469	-2.658759
H	-4.400563	0.033677	-0.917776
H	-3.420711	-2.352329	-1.445968
H	-3.311898	-1.805435	-3.097078
H	-1.821594	0.050730	-2.441942
H	-2.076056	-0.292904	-0.740642
H	-0.939150	-2.470206	-0.935361
H	-0.750765	-2.262296	-2.675419
H	0.450235	-0.420198	-0.554764
H	-2.649975	-3.431640	1.307831
H	1.081958	0.964837	-2.539173
H	0.310719	-0.251478	-3.577547
H	8.009897	-0.769964	-0.932702
H	7.367183	-1.759370	-2.257560
H	6.556655	-3.413221	-0.543551
H	7.323894	-2.404897	0.696422
C	-0.817820	-1.932023	2.125396
C	0.562613	-1.605139	2.613532
C	0.565531	-0.541346	3.717185
C	1.964530	-0.290813	4.284993
C	3.010930	0.097754	3.237193
C	2.614839	1.300567	2.383855
C	3.712972	1.707912	1.404363
C	3.341402	2.885277	0.501380
C	2.000095	2.719240	-0.205263
O	-1.546253	-0.875295	1.849517
O	-1.225698	-3.078602	1.985797
C	1.746263	3.706808	-1.372168
C	0.399574	4.109629	-0.755238
C	0.732026	3.257889	0.523549
C	-0.762194	3.149291	-1.075358
C	-0.506061	2.393890	0.294180
C	-2.099313	3.664549	-0.549509
C	-1.805834	2.997658	0.855664
C	-3.243857	2.664165	-0.781804
C	-3.013842	2.080016	0.661007
C	-4.632063	3.172274	-0.352755
C	-4.354003	2.715010	1.104023
H	0.892968	3.683598	1.518058
H	0.144227	5.171275	-0.738084
H	-1.730372	3.539881	1.803131
H	-2.325258	4.723858	-0.705604
H	-0.395513	1.310158	0.405217
H	-0.756347	2.674478	-2.062154
H	-2.883101	1.019593	0.876823

```
H          -3.180445        2.075010       -1.698959
H           1.034881       -2.533299        2.942929
H           1.117249       -1.240839        1.738795
H           0.143091        0.386176        3.317881
H          -0.097843       -0.863132        4.527294
H           1.893328        0.506384        5.034493
H           2.303811       -1.188899        4.815145
H           3.958495        0.312653        3.746210
H           3.213666       -0.758539        2.578571
H           1.701294        1.067217        1.818037
H           2.360099        2.147570        3.037200
H           4.629836        1.942666        1.959762
H           3.951470        0.845406        0.767939
H           3.324708        3.820548        1.078577
H           4.125170        3.007214       -0.258494
H           1.870628        1.668041       -0.496784
H          -2.472761       -1.147898        1.569092
H           1.719072        3.294222       -2.386327
H           2.467756        4.530096       -1.349218
H          -5.442111        2.601965       -0.815505
H          -4.819774        4.238081       -0.511978
H          -4.241249        3.521293        1.834495
H          -5.080622        1.996921        1.489631
```

VOA-d5LOH-M06-2X-D3-C21

__Requested operations__

Run with Gaussian 2016+B.01.

`freq=(VCD,ROA) CPHF=Rdfreq M062X/6-31+G(2d,p) SCRF=(Solvent=chloroform,Read) test`

__Relevant magnitudes__

Datum	Value
Charge	0
Multiplicity	1
Stoichiometry	C40H60O4
Number of Basis Functions	1400
Electronic Energy (Eh)	-1860.44833547
Sum of electronic and zero-point Energies (Eh)	-1859.514002
Sum of electronic and thermal Energies (Eh)	-1859.471223
Sum of electronic and enthalpy Energies (Eh)	-1859.470279

Sum of electronic and thermal Free Energies (Eh)	-1859.593035
Number of Imaginary Frequencies	0
Mean of alpha and beta Electrons	166

__Molecular Geometry in Cartesian Coordinates__

```xyz
C          3.205401        2.650261       -0.676505
C          4.592512        2.142438       -0.959703
C          4.682449        0.727532       -1.541237
C          4.247705        0.629388       -3.006233
C          2.740054        0.758824       -3.241303
C          1.944067       -0.394962       -2.632046
C          0.454362       -0.370274       -2.971217
C         -0.290332        0.826248       -2.381949
C         -1.800637        0.720615       -2.542531
O          2.412297        1.768621       -0.110407
O          2.860093        3.801652       -0.910225
C         -2.572287        1.839345       -1.790936
C         -3.109314        0.804129       -0.777398
C         -2.424844       -0.339138       -1.609803
C         -4.492383        0.257419       -1.141432
C         -3.806927       -0.861244       -2.026668
C         -4.988626       -0.818328       -0.168123
C         -4.324668       -1.939021       -1.069452
C         -6.376888       -1.358128       -0.528755
C         -5.719330       -2.459884       -1.440585
C         -6.871984       -2.464621        0.428978
C         -6.243387       -3.551680       -0.483640
H         -1.741202       -1.079521       -1.185352
H         -2.905862        0.987213        0.279137
H         -3.595893       -2.676141       -0.716069
H         -4.776551       -0.661235        0.894498
H         -3.984694       -1.012602       -3.095565
H         -5.233869        0.976243       -1.502998
H         -5.929128       -2.582289       -2.505252
H         -7.114164       -0.631976       -0.877635
H          5.084358        2.872497       -1.606614
H          5.105575        2.168352        0.010149
H          5.726164        0.406716       -1.458388
H          4.099651        0.038424       -0.920682
H          4.778827        1.395916       -3.584202
H          4.575510       -0.340417       -3.401120
H          2.374811        1.718892       -2.855932
H          2.554484        0.782911       -4.322930
H          2.380612       -1.339164       -2.985560
H          2.052524       -0.389482       -1.540055
```

H	0.320530	-0.381519	-4.061688
H	-0.006222	-1.292878	-2.593659
H	-0.060968	0.908765	-1.308319
H	0.054612	1.762317	-2.841307
H	-2.059820	0.599412	-3.601516
H	1.519295	2.182528	0.076155
H	-3.352771	2.348948	-2.363072
H	-1.905876	2.588853	-1.353399
H	-7.959589	-2.533181	0.492543
H	-6.468989	-2.399273	1.443568
H	-5.468275	-4.175774	-0.030015
H	-6.989324	-4.204161	-0.941677
C	-0.118096	4.049691	0.568388
C	-1.364329	4.611246	1.199770
C	-2.156637	3.570959	1.984664
C	-1.430531	3.107419	3.247507
C	-2.155244	1.995701	4.012980
C	-2.416808	0.722145	3.197961
C	-1.183889	0.200722	2.461219
C	-1.389887	-1.163306	1.805905
C	-0.175968	-1.594054	0.999123
O	0.716598	4.961825	0.123410
O	0.090722	2.849101	0.460582
C	-0.242255	-2.987578	0.322373
C	1.100755	-3.368531	0.967038
C	1.066326	-2.047232	1.816048
C	2.340795	-2.831795	0.219697
C	2.381141	-1.586432	1.198364
C	3.651956	-3.339549	0.810616
C	3.625543	-2.178524	1.884897
C	4.891043	-2.691100	0.159921
C	4.931364	-1.627351	1.319708
C	6.228142	-3.274179	0.651941
C	6.190311	-2.331573	1.883896
H	0.961096	-2.021912	2.904824
H	1.199091	-4.370814	1.389559
H	3.563812	-2.330001	2.966830
H	3.724198	-4.406289	1.043927
H	2.448927	-0.542196	0.878670
H	2.266506	-2.767727	-0.870419
H	4.999568	-0.548268	1.161282
H	4.800346	-2.442896	-0.901252
H	-1.068412	5.460645	1.824099
H	-1.958359	5.029179	0.377903
H	-3.129903	3.996057	2.253748
H	-2.352624	2.719210	1.325828
H	-0.425699	2.762988	2.977526

```
H          -1.295606        3.968274        3.914175
H          -1.550435        1.734535        4.890108
H          -3.109149        2.378856        4.396536
H          -2.794659       -0.055730        3.873436
H          -3.220155        0.901930        2.470811
H          -0.886716        0.919382        1.685475
H          -0.337922        0.143044        3.162082
H          -1.602819       -1.922202        2.572850
H          -2.270542       -1.131602        1.148407
H           0.088349       -0.794746        0.291885
H           1.508137        4.518977       -0.299573
H          -0.284227       -3.016748       -0.771767
H          -1.072049       -3.580482        0.721478
H           7.055996       -3.037322       -0.020271
H           6.235119       -4.349901        0.849261
H           6.018529       -2.828176        2.843117
H           7.066101       -1.687625        1.980687
```
